# Design, Synthesis, Biological Evaluation, and Molecular Dynamics Studies of Novel Lapatinib Derivatives

**DOI:** 10.3390/ph16010043

**Published:** 2022-12-28

**Authors:** Ahmed Elkamhawy, Seohyun Son, Hwa Young Lee, Mahmoud H. El-Maghrabey, Mohamed A. El Hamd, Saud O. Alshammari, Abeer A. Abdelhameed, Qamar A. Alshammari, Ahmed Abdeen, Samah F. Ibrahim, Wael A. Mahdi, Sultan Alshehri, Radwan Alnajjar, Won Jun Choi, Ahmed A. Al-Karmalawy, Kyeong Lee

**Affiliations:** 1 BK21 FOUR Team and Integrated Research Institute for Drug Development, College of Pharmacy, Dongguk University-Seoul, Goyang 10326, Republic of Korea; 2 Department of Pharmaceutical Organic Chemistry, Faculty of Pharmacy, Mansoura University, Mansoura 35516, Egypt; 3 Department of Pharmaceutical Analytical Chemistry, Faculty of Pharmacy, Mansoura University, Mansoura 35516, Egypt; 4 Department of Pharmaceutical Sciences, College of Pharmacy, Shaqra University, Shaqra 11961, Saudi Arabia; 5 Department of Pharmaceutical Analytical Chemistry, Faculty of Pharmacy, South Valley University, Qena 83523, Egypt; 6 Department of Plant Chemistry and Natural Products, Faculty of Pharmacy, Northern Border University, Arar 91431, Saudi Arabia; 7 Department of Pharmacology, Faculty of Medicine, Benha University, Benha 13518, Egypt; 8 Department of Pharmacology and Toxicology, Faculty of Pharmacy, Northern Border University, Arar 91431, Saudi Arabia; 9 Department of Forensic Medicine and Toxicology, Faculty of Veterinary Medicine, Benha University, Toukh 13736, Egypt; 10 Department of Clinical Sciences, College of Medicine, Princess Nourah bint Abdulrahman University, P.O. Box 84428, Riyadh 11671, Saudi Arabia; 11 Department of Pharmaceutics, College of Pharmacy, King Saud University, Riyadh 11451, Saudi Arabia; 12 Department of Chemistry, Faculty of Science, University of Benghazi, Benghazi 16063, Libya; 13 PharmD, Faculty of Pharmacy, Libyan International Medical University, Benghazi 16063, Libya; 14 Department of Chemistry, University of Cape Town, Rondebosch 7701, South Africa; 15 Pharmaceutical Chemistry Department, Faculty of Pharmacy, Ahram Canadian University, 6th of October City, Giza 12566, Egypt

**Keywords:** nitro group containing drugs, antiproliferative activity, EGFR/HER2 dual inhibition, synthesis, kinase panel, lapatinib, kinase assay, molecular docking, spectroscopic characterization

## Abstract

Co-expression of the epidermal growth factor receptor (EGFR, also known as ErbB1) and human epidermal growth factor receptor 2 (HER2) has been identified as a diagnostic or prognostic sign in various tumors. Despite the fact that lapatinib (EGFR/HER2 dual inhibitor) has shown to be successful, many patients do not respond to it or develop resistance for a variety of reasons that are still unclear. As a result, new approaches and inhibitory small molecules are still needed for EGFR/HER2 inhibition. Herein, novel lapatinib derivatives possessing 4-anilinoquinazoline and imidazole scaffolds (**6a**–**l**) were developed and screened as EGFR/HER2 dual inhibitors. In vitro and in silico investigations revealed that compound **6j** has a high affinity for the ATP-binding regions of EGFR and HER2. All of the designed candidates were predicted to not penetrate the BBB, raising the expectation for the absence of CNS side effects. At 10 µM, derivatives possessing 3-chloro-4-(pyridin-2-ylmethoxy)aniline moiety (**6i**–**l**) demonstrated outstanding ranges of percentage inhibition against EGFR (97.65–99.03%) and HER2 (87.16–96.73%). Compound **6j** showed nanomolar IC_50_ values over both kinases (1.8 nM over EGFR and 87.8 nM over HER2). Over EGFR, compound **6j** was found to be 50-fold more potent than staurosporine and 6-fold more potent than lapatinib. A kinase selectivity panel of compound **6j** showed poor to weak inhibitory activity over CDK2/cyclin A, c-MET, FGFR1, KDR/VEGFR2, and P38a/MAPK14, respectively. Structure–activity relationship (SAR) that were obtained with different substitutions were justified. Additionally, molecular docking and molecular dynamics studies revealed insights into the binding mode of the target compounds. Thus, compound **6j** was identified as a highly effective and dual EGFR/HER2 inhibitor worthy of further investigation.

## 1. Introduction

Protein tyrosine kinases have a fundamental role in signal transduction pathways that manage numerous cellular functions such as proliferation, differentiation, migration, and angiogenesis [1]. Accordingly, kinase inhibitors targeting the elevated pathways are promising candidates against cancer [2]. Indeed, overexpression of the epidermal growth factor receptor (EGFR or ErbB1) and human epidermal growth factor receptor 2 (HER2 or ErbB2) belonging to the ErbB family of receptor tyrosine kinases (RTKs) is commonly detected in several solid tumors [3,4,5,6]. In addition, EGFR and HER2 have been validated as rational targets for cancer-related treatment [7]. The process of epidermal growth factor (EGF) stimulation triggers the ErbB receptors to form either homo- or heterodimers with the other ErbB family receptors. This stimulates downstream signaling and promotes tumor cell growth [8]. Co-expression of EGFR and HER2 protein tyrosine kinases has been found in different tumors including ovarian, colon, breast, and prostate cancers [9,10,11,12]. HER2 gene amplification and receptor overexpression is identified in 20–25% of human breast cancers [13]. Thus, dual targeting of EGFR/HER2 was found to be more effective rather than only EGFR inhibition for the treatment of breast cancer [14]. Small molecules that inhibit EGFR/HER2 can prevent the process of tyrosine kinase phosphorylation and accordingly, suppress the upregulated intracellular signals in cancer cells. This results in the loss of the tumor regulatory function. Numerous ATP-competitive EGFR/HER2 RTK dual inhibitory small molecules possessing various chemical structures have been reported for the treatment of cancer [15,16].

**Figure 1:**
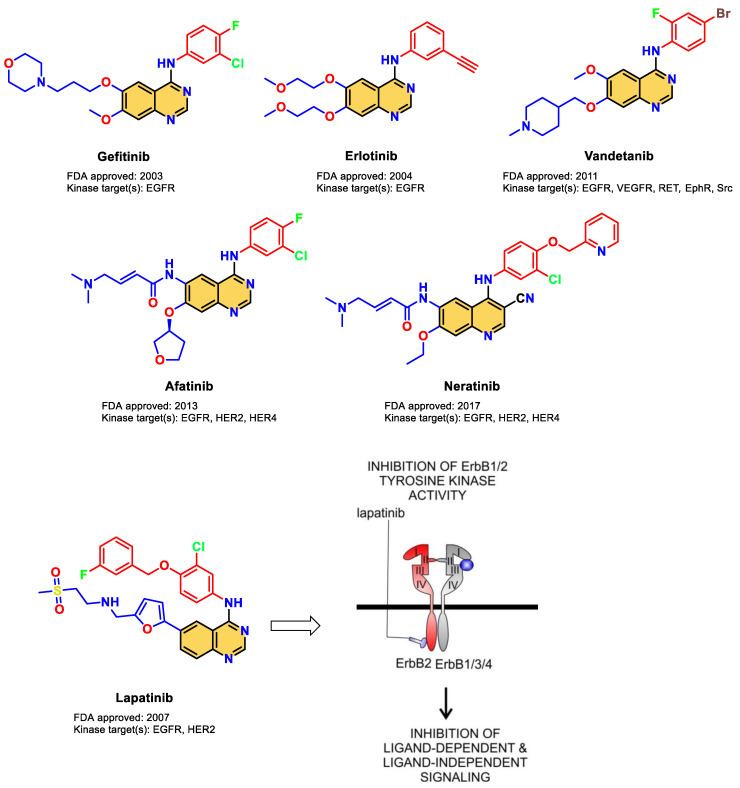
FDA-approved EGFR kinase inhibitors and the mechanism of action of Lapatinib [33].

As illustrated in Figure 1, 4-anilinoquinazoline is a leading chemical scaffold of EGFR/HER2 dual inhibitors, which is exemplified by lapatinib (FDA-approved therapy for HER2 overexpression metastatic breast cancer) [14]. It has been extensively researched in the literature on how lapatinib binds to the catalytic domain of EGFR/HER2 kinases [7,17,18]. Typically, the hinge region (found in the ATP binding site) is hydrogen bound to the quinazoline ring. The aniline group on the quinazoline scaffold (at C4 position) is angled deeply to fit in a nearby back pocket and create hydrophobic interactions. The structural modification of the C4 aniline group was the focus of the structure–activity relationship (SAR) research of the quinazoline moiety due to its role in kinase selectivity [19,20]. Previous research has shown that the kinase inhibitor selectivity is mostly influenced by the size and functioning of this back pocket, while the solubilizing moieties at the quinazoline core’s C6 and C7 improve the physical characteristics in order to achieve a positive pharmacokinetic profile. Additionally, various dual inhibitors were synthesized to bind to Cys773 in EGFR and Cys805 in HER2 via covalent or hydrogen bonds [21,22,23,24,25]. Despite the fact that lapatinib therapy has been shown to be successful, many patients do not respond to it or develop resistance for a variety of reasons that are still unclear [26,27,28,29]. As a result, novel methods and inhibitory small molecules are still required for EGFR/HER2 inhibition.

Tumor cells have a unique hypoxic microenvironment, which makes them a desirable and effective treatment target. Since the 1960s, derivatives of 2-nitroimidazole have been established as hypoxia-activated radio-sensitization and chemotherapeutic drugs [30]. Nitroreductase reduces the 2-nitroimidazole moiety in the presence of hypoxia to produce reactive radicals, which could deplete tumor-specific antioxidants such as glutathione (GSH), making tumors more radiotherapy-sensitive [31]. Furthermore, reactive radicals accumulate in cells and have lethal consequences due to their irreversible attachment to the protein and nucleic acids of those cells [32].

**Figure 2:**
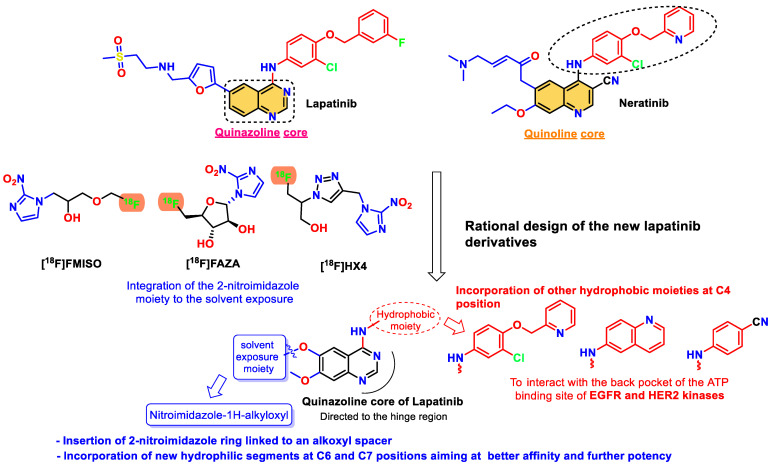
Design of the new lapatinib derivatives **6a**–**l**.

Up to now, numerous 2-nitroimidazole containing small molecules have been reported to have potential antitumor activities, with some of them advanced into clinical trials for cancer, such as [^18^F]FMISO, [^18^F]FAZA, and [^18^F] HX4 (Figure 2) [34,35,36]. Accordingly, to selectively inhibit EGFR and HER2, as shown in Figure 2, we concentrate our efforts in this research on designing and synthesizing new lapatinib derivatives bearing 6-(nitroimidazole-1*H*-alkyloxyl) moiety linked to the most common 4-anilinoquinazoline core.

As demonstrated in Figure 2, the rational design of the new candidates comes with three aniline patterns at C-4 of the quinazoline core to be directed into the deep hydrophobic pocket into the binding site, including the 3-chloro-4-(pyridin-2-ylmethoxy)aniline moiety of neratinib (2020 FDA-approved EGFR/HER2 dual inhibitor), quinolin-6-amine, and 4-aminobenzonitrile. To attain higher affinity and potency of the newly synthesized small molecules (**6a**–**l**), C6 and C7 positions were subjected to a variety of different polar/solubilizing functional groups. Indeed, gefitinib methoxy moiety was presented in all new derivatives to enhance their solubilizing properties. Furthermore, to investigate the effect of the nitroimidazole-1*H*-alkyloxyl moiety at the C6 position of the quinazoline core, the integration of various alkoxy spacers with different lengths was applied. The substitution patterns in the new series were planned to enhance the potency as well as selectivity via the integration of new moieties at C6 of the substituted 4-anilinoquinazolines and/or by changing the C4 aniline moieties. The new small molecules were assessed over EGFR and HER2 kinases in a single dose percent inhibition mode. The most active compound was further assessed for its IC_50_ values over both isoforms. A small kinase panel was then applied to check the selectivity of the most potent compound. In addition, detailed simulation studies were performed to understand the binding affinities and alignment of the new compounds at the ATP binding site of both kinases.

## 2. Materials and Methods

### 2.1. Chemical Reagents, Purification, and Instrumentation

The general protocols employed in the synthetic procedures, structure elucidation of the new chemical structures, and purity of the newly synthesized lapatinib derivatives were performed as reported earlier, with some modifications [37,38,39,40]. In brief, all acquired solvents and reagents were utilized without additional purification. Dimethyl sulfoxide (DMSO) was used as a solvent for NMR analysis. ^1^H NMR spectra were acquired using Bruker 400 MHz spectrometer, with chemical shifts being determined in parts per million (ppm) and coupling constants in Hz. ^13^C NMR spectra were acquired using Varian 100 MHz spectrometer (Varian Medical Systems, Inc., Palo Alto, CA, USA). The G2 QTOF mass spectrometer (Waters Corporation, Milford, MA, USA) was employed to produce the mass spectra, high-resolution mass spectrometry (HRMS, ESI-MS). Reaction monitoring was performed using TLC on 0.25 mm silica plates (E. Merck; silica gel 60 F_254_). To verify the purity of the target compounds, high-performance liquid chromatography (HPLC); System: Waters Corp. 2695 with PAD 996, λ = 281 nm; Column: Welch Xtimate C_18_ 150 mm × 4.6 mm × 5 μm; Condition: mobile phase A: 0.05% TFA in water, mobile phase B: CH_3_CN; Gradient: 90% of Mobile phase B in 30 min. The melting points (M.p.) were assessed with Thermo Scientific 9200 (Rise 10 °C per minute).

### 2.2. Synthesis of 1-(n-Bromoalkyl)-2-nitro-1H-imidazoles *(**2a**–**d**)*

To a dry round-bottom flask containing 2-nitroimidazole (**1**, 1.0 g, 8.84 mmol), the appropriate 1,n-dibromoalkane (17.7 mmol, 2.0 equiv.) was added in the presence of potassium carbonate (K_2_CO_3_, 2.4 g, 17.7 mmol, 2.0 equiv.) and dimethylformamide solvent (DMF, 15 mL). The reaction mixture was stirred at 60 °C for 4 h. The mixture was then partitioned using ethyl acetate (EA) and water. The organic layer was separated, evaporated, dried over anhydrous magnesium sulfate, and further purified using flash column chromatography (SiO_2_, *n*-hexane:EA = 5:1) to obtain the target 1-(n-bromoalkyl)-2-nitro-1*H*-imidazoles in suitable yields.

#### 2.2.1. 1-(3-Bromopropyl)-2-nitro-1H-imidazole (**2a**)

Light green solid. Yield: 51.7% (1.07 g, 4.57 mmol). ^1^H NMR (400 MHz, DMSO-*d_6_*) *δ* 7.66 (s, 1H), 7.17 (s, 1H), 4.48 (t, *J* = 6.0 Hz, 2H), 3.51 (t, *J* = 6.0 Hz, 2H), 2.36–2.30 (m, 2H). Reported [32,36].

#### 2.2.2. 1-(4-Bromobutyl)-2-nitro-1H-imidazole (**2b**)

Yellow oil. Yield: 54.7% (1.20 g, 4.84 mmol). ^1^H NMR (400 MHz, DMSO-*d_6_*) *δ* 7.68 (s, 1H), 7.17 (s, 1H), 4.40 (t, *J* = 8.0 Hz, 2H), 3.53 (t, *J* = 8.0 Hz, 2H), 1.92–1.85 (m, 2H), 1.81–1.74 (m, 2H). Reported [36,41].

#### 2.2.3. 1-(5-Bromopentyl)-2-nitro-1H-imidazole (**2c**)

Yellow oil. Yield: 62.1% (1.44 g, 5.49 mmol). ^1^H NMR (400 MHz, DMSO-*d_6_*) *δ* 7.69 (d, *J* = 4.0 Hz, 1H), 7.17 (d, *J* = 4.0 Hz, 1H), 4.37 (t, *J* = 8.0 Hz, 2H), 3.51 (t, *J* = 6.0 Hz, 2H), 1.85–1.74 (m, 4H), 1.41–1.34 (m, 2H). Reported [36,41].

#### 2.2.4. 1-(6-Bromohexyl)-2-nitro-1H-imidazole (**2d**)

Yellow oil. Yield: 63.5% (1.55 g, 5.61 mmol). ^1^H NMR (400 MHz, DMSO-*d_6_*) *δ* 7.68 (d, *J* = 1.2 Hz, 1H), 7.16 (d, *J* = 0.8 Hz, 1H), 4.35 (t, *J* = 8.0 Hz, 2H), 3.50 (t, *J* = 8.0 Hz, 2H), 1.81–1.72 (m, 4H), 1.43–1.36 (m, 2H), 1.31–1.24 (m, 2H). Reported [36,41].

### 2.3. Synthesis of Intermediate Acetates ***4a**–**c***

To a dry round-bottom flask containing 4-chloro-7-methoxyquinazolin-6-yl acetate (**3**, 2.0 g, 7.9 mmol), the appropriate aniline reagent (9.5 mmol, 1.2 equiv.) was added in the presence of isopropyl alcohol solvent (*i*-PrOH, 30 mL). The reaction mixture was refluxed for 4 h. After completion of the reaction, the mixture was then filtered using *i*-PrOH, and dried to obtain the desired intermediate without further purification.

#### 2.3.1. 7-Methoxy-4-(quinolin-6-ylamino)quinazolin-6-yl acetate (**4a**) 

Yellow solid. Yield: 93.5% (2.67 g, 7.41 mmol). ^1^H NMR (400 MHz, DMSO-*d_6_*) *δ* 11.26 (s, 1H), 8.98 (d, *J* = 4.0 Hz, 1H), 8.89 (s, 1H), 8.70 (s, 1H), 8.59 (d, *J* = 8.1 Hz, 1H), 8.50 (s, 1H), 8.20 (dd, *J* = 23.3, 9.0 Hz, 2H), 7.69 (dd, *J* = 8.3, 4.5 Hz, 1H), 7.48 (s, 1H), 4.00 (s, 3H), 2.38 (s, 3H). Reported [42].

#### 2.3.2. 4-((4-Cyanophenyl)amino)-7-methoxyquinazolin-6-yl acetate (**4b**)

Ivory solid. Yield: 99% (2.65 g, 7.92 mmol). ^1^H NMR (400 MHz, DMSO-*d_6_*) *δ* 11.07 (s, 1H), 8.92 (s, 1H), 8.65 (s, 1H), 8.04 (d, *J* = 8.0 Hz, 2H), 7.91 (d, *J* = 8.0 Hz, 2H), 7.47 (s, 1H), 3.99 (s, 3H), 2.37 (s, 3H). Reported [42].

#### 2.3.3. 4-((3-Chloro-4-(pyridin-2-ylmethoxy)phenyl)amino)-7-methoxyquinazolin-6-yl acetate (**4c**)

Light yellow solid. Yield: 80.5% (2.87 g, 6.38 mmol). ^1^H NMR (400 MHz, DMSO-*d_6_*) *δ* 11.04 (s, 1H), 8.86 (s, 1H), 8.60 (d, *J* = 4.0 Hz, 1H), 8.56 (s, 1H), 7.90–7.88 (m, 2H), 7.61–7.57 (m, 2H), 7.41–7.38 (m, 2H), 7.32 (d, *J* = 8.0 Hz, 1H), 5.32 (s, 2H), 3.99 (s, 3H), 2.37 (s, 3H).

### 2.4. Synthesis of Pre-Final Intermediates ***5a**–**c***

To a dry round-bottom flask containing the appropriate acetate intermediate (**4**, 3–8 mmol), the addition of an excessive amount of aqueous ammonia solution (28%) was carried out in methanol solvent (CH_3_OH, 150 mL). The reaction mixture was stirred at room temperature for 4 h. The mixture was partially evaporated from the excess solvent, filtered with cold methanol, and dried to obtain the desired intermediate without further purification.

#### 2.4.1. 7-Methoxy-4-(quinolin-6-ylamino)quinazolin-6-ol (**5a**)

Brownish-yellow solid. Yield: 61.5% (1.36 g, 4.27 mmol). ^1^H NMR (400 MHz, DMSO-*d_6_*) *δ* 9.65 (s, 1H), 8.77 (d, *J* = 4.0 Hz, 1H), 8.56 (s, 1H), 8.51 (s, 1H), 8.29 (d, *J* = 8.0 Hz, 1H), 8.18 (d, *J* = 8.0 Hz, 1H), 7.98 (d, *J* = 8.0 Hz, 1H), 7.87 (s, 1H), 7.47 (q, *J* = 4.0 Hz, 1H), 7.22 (s, 1H), 3.97 (s, 3H)). ^13^C NMR (100 MHz, DMSO-*d_6_*) *δ* 156.54, 154.37, 152.53, 149.22, 147.22, 146.75, 145.07, 138.43, 135.76, 129.29, 128.70, 126.21, 122.06, 117.35, 110.27, 107.64, 105.91, 56.39. HRMS (ESI) *m/z* calculated for C_18_H_15_N_4_O_2_ [M+H]^+^: 319.1195, Found: 319.1181. Reported [42].

#### 2.4.2. 4-((6-Hydroxy-7-methoxyquinazolin-4-yl)amino)benzonitrile (**5b**)

Ivory solid. Yield: 84.2% (1.95 g, 6.67 mmol). ^1^H NMR (400 MHz, DMSO-*d_6_*) *δ* 9.71 (s, 1H), 8.53 (s, 1H), 8.14 (d, *J* = 8.0 Hz, 2H), 7.81–7.77 (m, 3H), 7.23 (s, 1H), 3.97 (s, 3H). ^13^C NMR (100 MHz, DMSO-*d_6_*) *δ* 156.00, 154.63, 152.13, 147.47, 147.05, 144.96, 133.21, 121.07, 119.86, 110.48, 107.63, 105.73, 104.12, 56.43. HRMS (ESI) *m/z* calculated for C_16_H_13_N_4_O_2_ [M+H]^+^: 293.1039, Found: 293.1035. Reported [42].

#### 2.4.3. 4-((3-Chloro-4-(pyridin-2-ylmethoxy)phenyl)amino)-7-methoxyquinazolin-6-ol (**5c**)

Ivory solid. Yield: 86.1% (1.12 g, 2.75 mmol). ^1^H NMR (400 MHz, DMSO-*d_6_*) *δ* 9.59 (s, 1H), 9.32 (s, 1H), 8.56 (d, *J* = 8.0 Hz, 1H), 8.40 (s, 1H), 8.04 (d, *J* = 4.0 Hz, 1H), 7.86 (td, *J* = 8.0, 1.6 Hz, 1H), 7.74 (s, 1H), 7.70 (dd, *J* = 9.2, 2.4 Hz, 1H), 7.56 (d, *J* = 8.0 Hz, 1H), 7.36–7.33 (m, 1H), 7.22 (d, *J* = 12.0 Hz, 1H), 7.17 (s, 1H), 5.26 (s, 2H), 3.95 (s, 3H). ^13^C NMR (100 MHz, DMSO-*d_6_*) *δ* 156.90, 156.48, 154.23, 152.53, 149.60, 149.56, 147.05, 146.40, 137.53, 134.37, 123.85, 123.47, 122.07, 121.82, 121.25, 114.60, 109.91, 107.53, 105.81, 71.62, 56.35. HRMS (ESI) *m/z* calculated for C_21_H_18_ClN_4_O_3_ [M+H]^+^: 409.1067, Found: 409.1060.

### 2.5. Synthesis of the Target Lapatinib Derivatives ***6a**–**l***

To a dry round-bottom flask containing the appropriate pre-final intermediate (**5**, 0.31 mmol) in DMF (5 mL), potassium carbonate (86.8 mg, 0.63 mmol, 2.0 equiv.) and the appropriate bromoalkyl imidazole (0.37 mmol, 1.2 equiv.) were added at room temperature. The resulting mixture was stirred for 4 h at 80 °C. After completion of the reaction, the mixture was extracted with EA/water, the organic layer was dried over MgSO_4_ and concentrated. The crude was purified by column chromatography (5% MeOH/DCM, gradient elution) to obtain the final target lapatinib derivative.

#### 2.5.1. 7-Methoxy-6-(3-(2-nitro-1H-imidazol-1-yl)propoxy)-N-(quinolin-6-yl)quinazolin-4-amine (**6a**)

Yellow solid (73.5 mg, 0.16 mmol). M.p.: 249–250 °C. HPLC purity: 98.30% (retention time (RT = 6.286 min). ^1^H NMR (400 MHz, DMSO-*d_6_*) *δ* 9.73 (s, 1H), 8.79 (dd, *J* = 4.2, 1.4 Hz, 1H), 8.54 (s, 1H), 8.45 (d, *J* = 4.0 Hz, 1H), 8.32 (d, *J* = 8.0 Hz, 1H), 8.15 (dd, *J* = 9.0, 2.2 Hz, 1H), 8.02 (d, *J* = 12.0 Hz, 1H), 7.91 (s, 1H), 7.68 (s, 1H), 7.51–7.47 (m, 1H), 7.22 (s, 1H), 7.18 (d, *J* = 0.4 Hz, 1H), 4.63 (t, *J* = 8.0 Hz, 2H), 4.22 (t, *J* = 6.0 Hz, 2H), 3.93 (s, 3H), 2.43–2.36 (m, 2H). ^13^C NMR (100 MHz, DMSO-*d_6_*) *δ* 156.70, 154.96, 153.34, 149.45, 148.37, 147.66, 145.25, 145.20, 138.03, 135.74, 129.36, 128.67, 128.35, 128.24, 126.56, 122.12, 118.14, 109.38, 107.79, 103.49, 66.55, 56.34, 47.45, 29.62. HRMS (ESI) *m/z* calculated for C_24_H_22_N_7_O_4_ [M+H]^+^: 472.1733, Found: 472.1733.

#### 2.5.2. 7-Methoxy-6-(4-(2-nitro-1H-imidazol-1-yl)butoxy)-N-(quinolin-6-yl)quinazolin-4-amine (**6b**) 

Yellow solid (125.8 mg, 0.26 mmol). M.p.: 201–202 °C. HPLC purity: 99.41% (RT = 6.956 min). ^1^H NMR (400 MHz, DMSO-*d_6_*) *δ* 9.74 (s, 1H), 8.79 (dd, *J* = 4.0, 1.6 Hz, 1H), 8.53 (s, 1H), 8.45 (d, *J* = 4.0 Hz, 1H), 8.32 (d, *J* = 8.0 Hz, 1H), 8.16 (dd, *J* = 8.8, 2.0 Hz, 1H), 8.02 (d, *J* = 8.0 Hz, 1H), 7.91 (s, 1H), 7.76 (s, 1H), 7.50 (dd, *J* = 8.0, 4.0 Hz, 1H), 7.21 (d, *J* = 4.0 Hz, 1H), 4.51 (t, *J* = 8.0 Hz, 2H), 4.21 (t, *J* = 6.0 Hz, 2H), 3.93 (s, 3H), 2.05–1.98 (m, 2H), 1.87–1.80 (m, 2H). ^13^C NMR (100 MHz, DMSO-*d_6_*) *δ* 156.68, 154.94, 153.25, 149.44, 148.63, 147.52, 145.26, 145.07, 138.05, 135.73, 129.34, 128.67, 128.37, 128.32, 126.61, 122.11, 118.17, 109.44, 107.79, 103.28, 68.95, 56.35, 49.71, 27.15, 25.89. HRMS (ESI) *m/z* calculated for C_25_H_24_N_7_O_4_ [M+H]^+^: 486.1890, Found: 486.1890.

#### 2.5.3. 7-Methoxy-6-((5-(2-nitro-1H-imidazol-1-yl)pentyl)oxy)-N-(quinolin-6-yl)quinazolin-4-amine (**6c**)

Yellow solid (107 mg, 0.21 mmol). M.p.: 194–195 °C. HPLC purity: 99.55% (RT = 7.544 min). ^1^H NMR (400 MHz, DMSO-*d_6_*) *δ* 9.74 (s, 1H), 8.79 (dd, *J* = 4.2, 1.4 Hz, 1H), 8.53 (s, 1H), 8.45 (d, *J* = 4.0 Hz, 1H), 8.32 (d, *J* = 8.0 Hz, 1H), 8.16 (dd, *J* = 9.2, 2.4 Hz, 1H), 8.02 (d, *J* = 12.0 Hz, 1H), 7.89 (s, 1H), 7.72 (s, 1H), 7.49 (dd, *J* = 8.4, 4.0 Hz, 1H), 7.20 (d, *J* = 12.0 Hz, 1H), 4.43 (t, *J* = 8.0 Hz, 2H), 4.16 (t, *J* = 8.0 Hz, 2H), 3.93 (s, 3H), 1.92–1.84 (m, 4H), 1.52–1.45 (m, 2H). ^13^C NMR (100 MHz, DMSO-*d_6_*) *δ* 156.66, 154.92, 153.18, 149.43, 148.77, 147.46, 145.26, 145.02, 138.07, 135.73, 129.33, 128.67, 128.35, 128.30, 126.64, 122.11, 118.18, 109.46, 107.74, 103.04, 69.05, 56.31, 49.76, 30.01, 28.58, 23.01. HRMS (ESI) *m/z* calculated for C_26_H_26_N_7_O_4_ [M+H]^+^: 500.2046, Found: 500.2046.

#### 2.5.4. 7-Methoxy-6-((6-(2-nitro-1H-imidazol-1-yl)hexyl)oxy)-N-(quinolin-6-yl)quinazolin-4-amine (**6d**)

Yellow solid (111 mg, 0.22 mmol). M.p.: 176–177 °C. HPLC purity: 97.14% (RT = 8.404 min). ^1^H NMR (400 MHz, DMSO-*d_6_*) *δ* 9.74 (s, 1H), 8.79 (dd, *J* = 4.2, 1.4 Hz, 1H), 8.53 (s, 1H), 8.45 (d, *J* = 4.0 Hz, 1H), 8.32 (d, *J* = 8.0 Hz, 1H), 8.17 (dd, *J* = 9.4, 2.2 Hz, 1H), 8.02 (d, *J* = 12.0 Hz, 1H), 7.89 (s, 1H), 7.69 (s, 1H), 7.49 (dd, *J* = 8.4, 4.4 Hz, 1H), 7.21 (s, 1H), 7.16 (d, *J* = 0.8 Hz, 1H), 4.39 (t, *J* = 8.0 Hz, 2H), 4.15 (t, *J* = 8.0 Hz, 2H), 3.93 (s, 3H), 1.87–1.78 (m, 4H), 1.55–1.48 (m, 2H), 1.43–1.35 (m, 2H). ^13^C NMR (100 MHz, DMSO-*d_6_*) *δ* 156.65, 154.92, 153.17, 149.43, 148.82, 147.45, 145.25, 144.99, 138.08, 135.73, 129.32, 128.67, 128.29, 128.25, 126.64, 122.10, 118.17, 109.46, 107.74, 102.99, 69.12, 56.31, 49.81, 30.18, 28.94, 26.06, 25.57. HRMS (ESI) *m/z* calculated for C_27_H_28_N_7_O_4_ [M+H]^+^: 514.2203, Found: 514.2203.

#### 2.5.5. 4-((7-Methoxy-6-(3-(2-nitro-1H-imidazol-1-yl)propoxy)quinazolin-4-yl)amino)benzonitrile (**6e**)

Yellow solid (66.2 mg, 0.15 mmol). M.p.: 245–246 °C. HPLC purity: 99.52% (RT = 9.219 min). ^1^H NMR (400 MHz, DMSO-*d_6_*) *δ* 9.74 (s, 1H), 8.57 (s, 1H), 8.08 (d, *J* = 8.0 Hz, 2H), 7.83 (d, *J* = 1.2 Hz, 2H), 7.81 (s, 1H), 7.66 (d, *J* = 0.8 Hz, 1H), 7.23 (s, 1H), 7.17 (d, *J* = 0.8 Hz, 1H), 4.61 (t, *J* = 6.0 Hz, 2H), 4.20 (t, *J* = 6.0 Hz, 2H), 3.93 (s, 3H), 2.48–2.35 (m, 2H). ^13^C NMR (100 MHz, DMSO-*d_6_*) *δ* 156.16, 155.22, 152.95, 148.56, 147.97, 145.20, 144.60, 133.26, 128.34, 128.23, 121.61, 119.74, 109.61, 107.79, 104.60, 103.36, 66.59, 56.40, 47.42, 29.59. HRMS (ESI) *m/z* calculated for C_22_H_20_N_7_O_4_ [M+H]^+^: 446.1577, Found: 446.1577.

#### 2.5.6. 4-((7-Methoxy-6-(4-(2-nitro-1H-imidazol-1-yl)butoxy)quinazolin-4-yl)amino)benzonitrile (**6f**)

Yellow solid (95.9 mg, 0.21 mmol). M.p.: 200–201 °C. HPLC purity: 99.00% (RT = 10.005 min). ^1^H NMR (400 MHz, DMSO-*d_6_*) *δ* 9.75 (s, 1H), 8.56 (s, 1H), 8.09 (d, *J* = 8.0 Hz, 1H), 7.84 (d, *J* = 4.0 Hz, 2H), 7.81 (s, 1H), 7.75 (d, *J* = 0.4 Hz, 1H), 7.23 (s, 1H), 7.19 (d, *J* = 0.8 Hz, 1H), 4.50 (t, *J* = 8.0 Hz, 2H), 4.19 (t, *J* = 6.0 Hz, 2H), 3.93 (s, 3H), 2.04–1.97 (m, 2H), 1.86–1.79 (m, 2H). ^13^C NMR (100 MHz, DMSO-*d_6_*) *δ* 156.12, 155.18, 152.85, 148.82, 147.83, 145.06, 144.61, 133.23, 128.35, 128.31, 121.62, 119.75, 109.66, 107.77, 104.58, 103.15, 68.99, 56.40, 49.70, 27.15, 25.87. HRMS (ESI) *m/z* calculated for C_23_H_22_N_7_O_4_ [M+H]^+^: 460.1733, Found: 460.1731.

#### 2.5.7. 4-((7-Methoxy-6-((5-(2-nitro-1H-imidazol-1-yl)pentyl)oxy)quinazolin-4-yl)amino)benzonitrile (**6g**)

Yellow solid (131.5 mg, 0.28 mmol). M.p.: 196–197 °C. HPLC purity: 98.40% (RT = 10.615 min). ^1^H NMR (400 MHz, DMSO-*d_6_*) *δ* 9.74 (s, 1H), 8.56 (s, 1H), 8.09 (d, *J* = 8.0 Hz, 2H), 7.83 (d, *J* = 4.0 Hz, 2H), 7.81 (s, 1H), 7.71 (d, *J* = 0.8 Hz, 1H), 7.22 (s, 1H), 7.18 (d, *J* = 0.4 Hz, 1H), 4.41 (t, *J* = 6.0 Hz, 2H), 4.14 (t, *J* = 6.0 Hz, 2H), 3.92 (s, 3H), 1.91–1.83 (m, 4H), 1.51–1.43 (m, 2H). ^13^C NMR (100 MHz, DMSO-*d_6_*) *δ* 156.11, 155.18, 152.80, 148.96, 147.78, 145.00, 144.63, 133.23, 128.35, 128.30, 121.64, 119.75, 109.68, 107.74, 104.57, 102.92, 69.08, 56.37, 49.74, 29.99, 28.55, 23.00. HRMS (ESI) *m/z* calculated for C_24_H_24_N_7_O_4_ [M+H]^+^: 474.1890, Found: 474.1890.

#### 2.5.8. 4-((7-Methoxy-6-((6-(2-nitro-1H-imidazol-1-yl)hexyl)oxy)quinazolin-4-yl)amino)benzonitrile (**6h**)

Yellow solid (115.4 mg, 0.24 mmol). M.p.: 191–192 °C. HPLC purity: 97.08% (RT = 11.432 min). ^1^H NMR (400 MHz, DMSO-*d_6_*) *δ* 9.75 (s, 1H), 8.56 (s, 1H), 8.09 (d, *J* = 8.0 Hz, 1H), 7.83 (s, 2H), 7.80 (s, 1H), 7.69 (d, *J* = 0.4 Hz, 1H), 7.22 (s, 1H), 7.16 (d, *J* = 0.8 Hz, 1H), 4.38 (t, *J* = 8.0 Hz, 2H), 4.13 (t, *J* = 8.0 Hz, 2H), 3.93 (s, 3H) 1.86–1.77 (m, 4H), 1.54–1.46 (m, 2H), 1.41–1.34 (m, 2H). ^13^C NMR (100 MHz, DMSO-*d_6_*) *δ* 156.10, 155.19, 152.79, 149.02, 147.77, 144.98, 144.64, 133.23, 128.28, 128.24, 121.65, 119.75, 109.69, 107.74, 104.57, 102.88, 69.16, 56.38, 49.80, 30.17, 28.91, 26.05, 25.56. HRMS (ESI) *m/z* calculated for C_25_H_26_N_7_O_4_ [M+H]^+^: 488.2046, Found: 488.2046.

#### 2.5.9. N-(3-Chloro-4-(pyridin-2-ylmethoxy)phenyl)-7-methoxy-6-(3-(2-nitro-1H-imidazol-1-yl)propoxy)quinazolin-4-amine (**6i**)

Yellow solid (76.6 mg, 0.14 mmol). M.p.: 184–185 °C. HPLC purity: 95.43% (RT = 9.322 min). ^1^H NMR (400 MHz, DMSO-*d_6_*) *δ* 9.39 (s, 1H), 8.59 (d, *J* = 4.0 Hz, 1H), 8.44 (s, 1H), 7.93 (d, *J* = 2.4 Hz, 1H), 7.87 (td, *J* = 7.6, 1.6 Hz, 1H), 7.78 (s, 1H), 7.66 (d, *J* = 0.4 Hz, 1H), 7.64 (d, *J* = 2.4 Hz, 1H), 7.57 (d, *J* = 8.0 Hz, 1H), 7.37–7.34 (m, 1H), 7.26 (d, *J* = 12.0 Hz, 1H), 7.17 (s, 2H), 5.27 (s, 2H), 4.61 (t, *J* = 8.0 Hz, 2H), 4.17 (t, *J* = 6.0 Hz, 2H), 3.91 (s, 3H), 2.48–2.34 (m, 2H). ^13^C NMR (100 MHz, DMSO-*d_6_*) *δ* 156.85, 156.65, 154.80, 153.37, 149.92, 149.55, 148.21, 147.44, 145.19, 137.52, 133.92, 128.33, 128.23, 124.40, 123.47, 122.58, 121.84, 121.32, 114.62, 109.06, 107.75, 103.39, 71.65, 66.48, 56.28, 47.44, 29.61. HRMS (ESI) *m/z* calculated for C_27_H_25_ClN_7_O_5_ [M+H]^+^: 562.1606, Found: 562.1606.

#### 2.5.10. N-(3-Chloro-4-(pyridin-2-ylmethoxy)phenyl)-7-methoxy-6-(4-(2-nitro-1H-imidazol-1-yl)butoxy)quinazolin-4-amine (**6j**)

Yellow solid (117.9 mg, 0.20 mmol). M.p.: 172–173 °C. HPLC purity: 98.85% (RT = 10.068 min). ^1^H NMR (400 MHz, DMSO-*d_6_*) *δ* 9.40 (s, 1H), 8.59 (d, *J* = 4.0 Hz, 1H), 8.43 (s, 1H), 7.94 (d, *J* = 4.0 Hz, 1H), 7.87 (td, *J* = 7.8, 2.0 Hz, 1H), 7.78 (s, 1H), 7.75 (d, *J* = 0.4 Hz, 1H), 7.66 (dd, *J* = 9.2, 2.4 Hz, 1H), 7.57 (d, *J* = 8.0 Hz, 1H), 7.37–7.34 (m, 1H), 7.26 (d, *J* = 8.0 Hz, 1H), 7.19 (d, *J* = 0.8 Hz, 1H), 7.17 (s, 1H), 5.27 (s, 2H), 4.50 (t, *J* = 6.0 Hz, 2H), 4.16 (t, *J* = 6.0 Hz, 2H), 3.91 (s, 3H), 2.03–1.96 (m, 2H), 1.85–1.78 (m, 2H). ^13^C NMR (100 MHz, DMSO-*d_6_*) *δ* 156.85, 156.64, 154.79, 153.28, 149.92, 149.56, 148.47, 147.30, 145.06, 137.52, 133.93, 128.35, 128.31, 124.43, 123.48, 122.63, 121.84, 121.31, 114.61, 109.13, 107.75, 103.18, 71.65, 68.90, 56.30, 49.70, 27.15, 25.88. HRMS (ESI) *m/z* calculated for C_28_H_27_ClN_7_O_5_ [M+H]^+^: 576.1762, Found: 576.1762.

#### 2.5.11. N-(3-Chloro-4-(pyridin-2-ylmethoxy)phenyl)-7-methoxy-6-((5-(2-nitro-1H-imidazol-1-yl)pentyl)oxy)quinazolin-4-amine (**6k**)

Yellow solid (110.2 mg, 0.19 mmol). M.p.: 158–159 °C. HPLC purity: 98.51% (RT = 10.634 min). ^1^H NMR (400 MHz, DMSO-*d_6_*) *δ* 9.40 (s, 1H), 8.59 (d, *J* = 4.0 Hz, 1H), 8.43 (s, 1H), 7.93 (d, *J* = 2.8 Hz, 1H), 7.87 (td, *J* = 7.6, 1.6 Hz, 1H), 7.77 (s, 1H), 7.71 (s, 1H), 7.66 (dd, *J* = 9.0, 2.6 Hz, 1H), 7.57 (d, *J* = 8.0 Hz, 1H), 7.37–7.34 (m, 1H), 7.26 (d, *J* = 12.0 Hz, 1H), 7.18 (d, *J* = 0.8 Hz, 1H), 7.16 (s, 1H), 5.27 (s, 2H), 4.42 (t, *J* = 6.0 Hz, 2H), 4.11 (t, *J* = 6.0 Hz, 2H), 3.91 (s, 3H), 1.91–1.82 (m, 4H), 1.51–1.43 (m, 2H). ^13^C NMR (100 MHz, DMSO-*d_6_*) *δ* 156.85, 156.63, 154.77, 153.22, 149.92, 149.56, 148.61, 147.24, 144.98, 137.53, 133.94, 128.35, 128.29, 124.45, 123.48, 122.65, 121.84, 121.31, 114.61, 109.14, 107.71, 102.94, 71.65, 68.99, 56.26, 49.75, 30.00, 28.57, 23.00. HRMS (ESI) *m/z* calculated for C_29_H_29_ClN_7_O_5_ [M+H]^+^: 590.1919, Found: 590.1921.

#### 2.5.12. N-(3-Chloro-4-(pyridin-2-ylmethoxy)phenyl)-7-methoxy-6-((6-(2-nitro-1H-imidazol-1-yl)hexyl)oxy)quinazolin-4-amine (**6l**)

Yellow solid (117.6 mg, 0.19 mmol). M.p.: 191–192 °C. HPLC purity: 97.79% (RT = 11.314 min). ^1^H NMR (400 MHz, DMSO-*d_6_*) *δ* 9.40 (s, 1H), 8.58 (d, *J* = 6.0 Hz, 1H), 8.42 (s, 1H), 7.94 (d, *J* = 6.0 Hz, 1H), 7.87 (td, *J* = 7.8, 1.2 Hz, 1H), 7.77 (s, 1H), 7.69 (s, 1H), 7.66 (dd, *J* = 9.0, 2.6 Hz, 1H), 7.57 (d, *J* = 8.0 Hz, 1H), 7.37–7.34 (m, 1H), 7.26 (d, *J* = 12.0 Hz, 1H), 7.16 (s, 1H), 5.27 (s, 2H), 4.38 (t, *J* = 8.0 Hz, 2H), 4.11 (t, *J* = 6.0 Hz, 2H), 3.91 (s, 3H), 1.85–1.77 (m, 4H), 1.53–1.46 (m, 2H), 1.41–1.34 (m, 2H). ^13^C NMR (100 MHz, DMSO-*d_6_*) *δ* 156.85, 156.62, 154.78, 153.21, 149.92, 149.56, 148.67, 147.23, 144.99, 137.52, 133.96, 128.28, 128.24, 124.45, 123.48, 122.66, 121.84, 121.31, 114.61, 109.16, 107.70, 102.90, 71.65, 69.07, 56.27, 49.80, 30.17, 28.92, 26.05, 25.56. HRMS (ESI) *m/z* calculated for C_30_H_31_ClN_7_O_5_ [M+H]^+^: 604.2075, Found: 604.2075.

### 2.6. In Vitro Kinase Assays

The in vitro kinase inhibitory assays were carried out at Reaction Biology Corp using Kinase HotSpot^SM^ technology in order to evaluate the inhibitory activities of the newly synthesized lapatinib derivatives, as previously carried out [17,43,44].

### 2.7. Molecular Docking Studies

**Figure 3:**
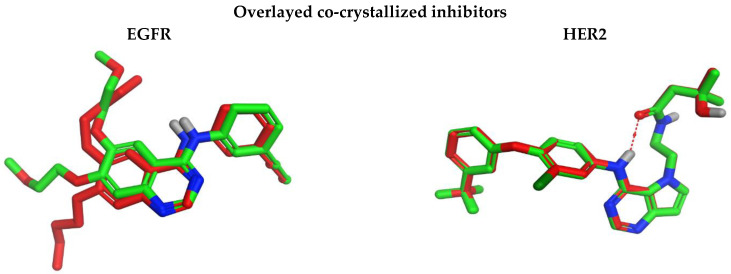
Overlaying of the native (green) and re-docked (red) co-crystallized ligands of EGFR and HER2 binding pockets.

The MOE 2019.0102 [45,46] was used to perform two different molecular docking studies for the newly designed candidates (**6a**–**l**) against both EGFR (PDB ID: 1M17 [47]) and HER2 (PDB ID: 3RCD [18]) receptors to investigate their dual inhibitory activities. The resolution values for both 1M17 and 3RCD are 2.60 and 3.21, respectively, indicating greatly acceptable and accurate X-ray structures of the two protein receptors. The co-crystallized inhibitor of 1M17 contains a quinazoline ring, and that of 3RCD contains a pyrrolopyrimidine ring which is greatly similar and isosteric to the main nucleus of our new target derivatives (quinazoline moiety). The co-crystallized inhibitors (AQ4 and 03P, respectively) were inserted as reference standards. First, to validate the used force field during the two applied docking processes, we carried out a separate redocking process for each co-crystallized inhibitor within its binding pocket. The valid performance was confirmed in each case by obtaining low root mean square deviation (RMSD) values (0.90 and 0.08 Å, respectively). Besides, the same binding mode was observed by overlaying both the native (green) and redocked (red) co-crystallized ligand in each binding pocket [48], Figure 3.

Then, to prepare the designed compounds (**6a**–**l**) for the docking processes, they were sketched using ChemDraw Professional program, introduced individually into the working window of the MOE, 3D hydrogenated, and energy minimized as previously described [49,50]. The prepared derivatives (**6a**–**l**) were inserted into two different databases besides the co-crystallized inhibitor (AQ4 or 03P) for EGFR and HER2 docking processes, respectively. Moreover, the X-ray structures of the target proteins were retrieved from the Protein Data Bank (IDs: 1M17 [47] and 3RCD [18]) for EGFR and HER2, respectively. Each protein was corrected, 3D hydrogenated, and energy minimized, as described before [51,52]. Finally, two general docking processes were carried out for EGFR and HER2 receptors using the appropriate database in each case. The program specifications and the full methodology for the general docking were applied, as stated earlier [53,54].

### 2.8. Molecular Dynamics (MD) Simulations and MM-GBSA Study

The MD simulations were performed using the Desmond package of Schrödinger LLC [55]. The most active compounds of the EGFR and HER2 receptors (**6j** and **6k**) were subjected to MD simulations runs for 200 ns compared to the co-crystallized inhibitor complex in each case (AQ4-1M17 and 03P-3RCD, respectively). Furthermore, the Molecular Mechanics Generalized Born Surface Area (MM-GBSA) energies for the aforementioned complexes were calculated through the thermal_mmgbsa.py python script of Schrödinger. Notably, the MD methodologies are described in detail in the Supplementary Information (SI 1 and SI 2).

## 3. Results and Discussion

### 3.1. Chemical Synthesis

**Scheme 1:**
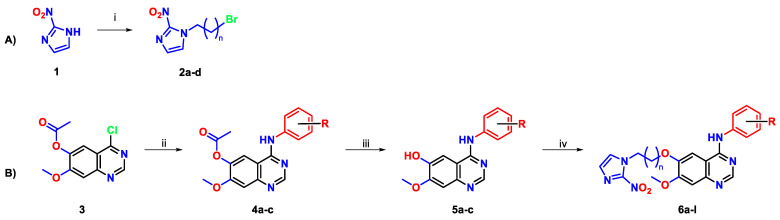
Reagents and conditions: (i) appropriate 1,n-dibromoalkane, K_2_CO_3_, DMF, 60 °C, 4 h; (ii) appropriate aniline reagent, isopropyl alcohol, reflux, 4 h; (iii) aqueous ammonia solution (28%), methanol, rt, 4 h; (iv) appropriate 1-(n-bromoalkyl)-2-nitro-1*H*-imidazole, K_2_CO_3_, DMF, 80 °C, 4 h.

As shown in Scheme 1, a new series of lapatinib derivatives **3a**–**l** was synthesized. First, to synthesize the 1-(n-bromoalkyl)-2-nitro-1*H*-imidazoles (**2a**–**d**, Scheme 1A), the commercially available 2-nitroimidazole (**1**) was used. The incorporation of the bromoalkyl chain into the 2-nitroimidazole was accomplished in DMF solvent using a variety of commercially available 1,n-dibromoalkanes, and K_2_CO_3_ as a catalytic inorganic base at 60 °C. Second, as illustrated in Scheme 1B, the commercially available 4-chloro-7-methoxyquinazolin-6-yl acetate (**3**) was refluxed in isopropyl alcohol separately, with three different aniline moieties that substituted the chloro group on the C4 position of the quinazoline chemical scaffold: quinolin-6-amine, 4-aminobenzonitrile, and 3-chloro-4-(pyridin-2-ylmethoxy)aniline to form intermediates **4a**–**c**. Intermediates **5a**–**c** were then formed via a hydrolysis reaction of the ester group of compounds **4a**–**c**. The hydrolysis reaction was performed using an aqueous ammonia solution (28%) in methanol solvent at room temperature. The free phenolic group in intermediates **5a**–**c** was then reacted with the previously synthesized 1-(n-bromoalkyl)-2-nitro-1*H*-imidazoles (**2a**–**d**) in DMF solvent in the presence of potassium carbonate at 80 °C to yield the target lapatinib derivatives **6a**–**l** (Table 1).

### 3.2. Structure Elucidation of the Newly Synthesized Lapatinib Derivatives ***6a**–**l***

Using various spectroscopic methods, such as ^1^H NMR, ^13^C NMR, and HRMS, the newly synthesized chemical structures of lapatinib derivatives bearing 6-(nitroimidazole-1*H*-alkyloxyl) moiety (**6a**–**l**) were identified. The ^1^H NMR spectra of the final compounds were confirmed by the presence of a singlet peak (3H) at around 3.93 ppm attributable to the protons of the methoxy group (–OCH_3_) at the C7 position of the quinazoline core. In addition, the proton at the C2 position of the quinazoline scaffold was identified as a singlet peak (1H) at a range of 8.42–8.57 ppm. In addition, all target compounds’ purity was determined by HPLC analysis, and the purities were found to be greater than 96%. As detailed in the supplementary material, the ^1^H NMR spectrum of the quinoline-based derivative **6a** exhibited the C2 position proton at 8.54 ppm as a singlet peak (1H). Additionally, the aliphatic carbon connected to the oxygen atom at the C6 position of the quinazoline ring was confirmed clearly in the ^13^C NMR spectrum at 66.55 ppm, which proved the formation of the O-alkylation. The same aliphatic carbon in the target compounds **6b**–**d** was also detected in the range of 68.95–69.12 ppm. The extended carbons in compound **6b**–**d** connected to the imidazole ring were founded with lower chemical shifts. The characteristic carbon of the nitrile group (–CN) in derivatives **6e**–**h** was detected in the range of 119.74–119.75 ppm in the ^13^C NMR spectra. The carbon bearing the nitrile group was found at around 104.60 ppm. Compound **6i** possessing 3-chloro-4-(pyridine-2-ylmethoxy)phenyl) moiety was characterized by two aliphatic protons at 5.27 ppm in the ^1^H NMR spectrum. In the meantime, its aliphatic carbon was detected at 71.65 ppm in the ^13^C NMR spectrum. The chemical structure of compounds **6i**–**l** was also confirmed via the various protons and carbons of the aliphatic linker bearing the imidazole ring in ^1^H NMR (range of 2.00–5.00 ppm) and ^13^C NMR spectra (range of 20.00–80.00 ppm), respectively. These findings supported the formation of the synthesized derivatives **6a**–**l**.

### 3.3. In Silico Druggability Studies of the Newly Synthesized Lapatinib Derivatives ***6a**–**l***

The SwissADME online server [56] was used to investigate the physicochemical properties, pharmacokinetics parameters, and lead likeness of the newly examined candidates (**6a**–**l**), Table 2. Analyzing the molecular physicochemical properties, it was clear that only compounds **6a** and **6b** were moderately soluble. However, compounds **6c**–**j** were poorly soluble, and compounds **6k** and **6l** were insoluble in H_2_O. Therefore, drug formulation studies are recommended for the newly studied derivatives. However, the prediction of the pharmacokinetic properties showed that all the synthesized compounds showed low GI absorption, which may be attributed to their low lipophilicity. Again, formulation studies are highly recommended for oral route testing, or an alternative route of administration may be investigated. Moreover, all of the designed candidates do not penetrate the BBB raising the expectation for the absence of CNS side effects. On the other hand, both compounds **6a** and **6b** are good substrates for P-glycoprotein and may be subjected to its efflux mechanism. Furthermore, all compounds exhibit good inhibiting power against CYP3A4, CYP2C9, and CYP2C19 metabolizing enzymes. However, only compounds **6a**–**e** are good inhibitors for CYP1A2, and compounds **6a**–**i** are good inhibitors for CYP2D6 metabolizing enzymes. Besides, compounds **6a**–**h** obeys Lipinski’s rule of five, suggesting them as promising drug candidates.

### 3.4. Biological Evaluaiton

#### 3.4.1. EGFR and HER2 Kinase Assay of Compounds **6a**–**l**

To evaluate the newly synthesized lapatinib derivatives **6a**–**l** as EGFR/HER2 potential dual inhibitors, they were preliminary evaluated over both targets using ‘HotSpot^SM^’ assay via applying a single dose concentration of 10 µM of each final compound at 10 µM concentration of ATP. Data were acquired as % of remaining kinase activity compared to vehicle (DMSO) reactions. The kinase inhibition results of all final compounds over both target enzymes (EGFR and HER2) were calculated and reported in Table 3. Regarding EGFR inhibitory activity, the biological results showed promising inhibitory activities of all the twelve compounds on EGFR kinase. The structure–activity relationship (SAR) of derivatives **6a**–**l** is proposed as follows: Derivatives with a quinoline moiety (**6a**–**d**) displayed an outstanding inhibitory range of 98.79–99.34%. On the other hand, incorporation of the benzonitrile moiety in derivatives **6e**–**h** was found to negatively affect the activity against EGFR, with a % inhibition range of 67.27–94.67%. All derivatives possessing the hydrophobic moiety of neratinib (3-chloro-4-(pyridin-2-ylmethoxy)aniline, **6i**–**l**) demonstrated a superior range of EGFR % inhibition (97.65–99.03%). Regarding the inhibitory activity against HER2 kinase, it was noted that most compounds exerted lower inhibitory activities over HER2. However, a similar SAR pattern (with different ranges of activities) was detected in the case of HER2 kinase. While compounds **6a**–**d** demonstrated high inhibitory activities ranging from 74.72–89.99%, derivatives **6e**–**h** showed a low to moderate inhibitory activity range (8.78–47.34%). The best inhibitory activity range was detected in compounds **6i**–**l**, with a range of 87.16–96.73%. Based on these findings, compound **6j**, with the most promising dual % inhibition over both kinases (99.03% over EGFR and 96.73% over HER2), was selected for further evaluation.

#### 3.4.2. Dose-Dependent Evaluation of Compound **6j** over EGFR and HER2

A dose-dependent assay was performed to evaluate the IC_50_ values of compound **6j** over EGFR and HER2 using a 10-dose assay of 3-fold serial dilution starting at 10 µM. As demonstrated in Table 4, compound **6j** showed outstanding nanomolar IC_50_ values over both kinases (1.8 nM over EGFR and 87.8 nM over HER2). The standard used in this assay (staurosporine) displayed an IC_50_ value of 88.1 nM over EGFR and 35.5 nM over HER2. Over EGFR, compound **6j** was found to be 50-fold more potent than staurosporine over EGFR and almost 6-fold higher potency than lapatinib.

#### 3.4.3. Kinase Selectivity Panel of Compound **6j**

To assess the preliminary selectivity profile of compound **6j**, an in vitro assay using a single-dose concentration of 10 µM was carried out against a small panel of cancer-associated kinases, including CDK2/cyclin A, c-MET, FGFR1, KDR/VEGFR2, and P38a/MAPK14. The outcomes of the selectivity study are demonstrated in Table 5. The results exhibited poor to weak inhibitory activity against the tested kinases with –0.83, 19.27, 13.87, 10.32, and –33.31% inhibition over CDK2/cyclin A, c-MET, FGFR1, KDR/VEGFR2, and P38a/MAPK14, respectively. These findings strongly revealed a remarkable selectivity of compound **6j**, taking into consideration its dual nanomolar potency over EGFR and HER2.

#### 3.4.4. Molecular Docking

Two different molecular docking studies were performed to investigate the dual inhibitory activities of the novel (**6a**–**l**) candidates against both EGFR (PDB ID: 1M17 [47]) and HER2 (PDB ID: 3RCD [18]) targets using the MOE 2019.0102 program [45,46]. The co-crystallized inhibitor of EGFR (4-anilinoquinazoline, AQ4) and that of HER2 (pyrrolo [3,2-*d*]pyrimidine, 03P) were used as reference standards. Herein, the two most active compounds biologically (**6j** and **6k**) were selected for further investigations.

Regarding the EGFR (PDB ID: 1M17 [47]) binding site (Table 6), it was observed that the co-crystallized AQ4 inhibitor became stabilized inside its binding pocket through the formation of four H-bonds with MET769, GLN767, CYS773, and LYS692. Besides, it formed three H_2_O (10)-bridged H-bonds with THR766, CYS751, and THR830. Notably, the docked co-crystallized AQ4 ligand showed a binding score of −7.90 kcal/mol and an RMSD value of 1.62 Å. On the other hand, compound **6j** (score = −9.00 kcal/mol and RMSD = 1.73 Å) formed two H-bonds with the two crucial amino acids (MET769 and GLN767) at 3.38 and 3.54 Å, respectively. In addition, it bound LEU694 with a pi-H bond at 4.33 Å. However, compound **6k** (score = −8.52 kcal/mol and RMSD = 1.69 Å) formed the same three H_2_O (10)-bridged H-bonds with THR766, CYS751, and THR830 crucial amino acids at 2.93 Å.

Concerning the HER2 (PDB ID: 3RCD [18]) binding site (Table 7), it was clear that the co-crystallized 03P inhibitor occupied its binding pocket with the formation of three H-bonds with MET801, LEU726, and LYS753. In addition, the docked co-crystallized 03P ligand achieved a binding score of −11.66 kcal/mol and an RMSD value of 1.22 Å. Moreover, compound **6j** (score = −11.31 kcal/mol and RMSD = 1.52 Å) formed two H-bonds with the crucial LYS753 amino acid at 3.08 and 3.09 Å. Furthermore, compound **6k** (score = −11.05 kcal/mol and RMSD = 1.55 Å) formed only one H-bond with the crucial MET801 amino acid at 3.65 Å.

Collectively, the superior binding scores and the closely similar binding modes of both **6j** and **6k** derivatives compared to AQ4 and 03P inhibitors confirm the superior antagonistic activities.

#### 3.4.5. Molecular Dynamics (MD) Simulations

Molecular dynamic simulations were implemented to mimic the behavior of the frontier compounds in a cell-like environment. Compounds **6j** and **6k** were selected, and their behavior was studied inside the active site of both the EGFR tyrosine kinase domain (1M17) and HER2 Kinase Domain (3RCD).

**Figure 4:**
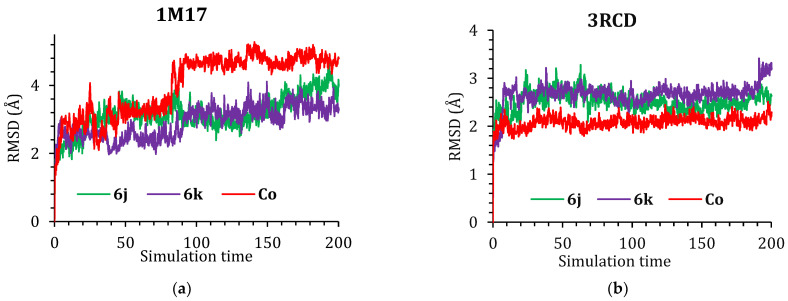
The RMSD of **6j**, **6k**, and Co-crystal ligand in complex with (**a**) 1M17 and (**b**) 3RCD.

The protein conformational change was monitored via the change in the Cα atom of the protein backbone position in Å, and it was plotted as a function of simulation time in Figure 4. The RMSD of ligands-1M117 complexes was 3.5 Å for complex **6j**-1M17 till around 170 ns of the simulation time, at which it rose to 4.00 Å, while in the case of complex **6k**-1M17, the complex was more stable and held an RMSD of 2.5 Å from the beginning of the simulation till 90 ns, where the RMSD rose to 3.5 Å and hold steady toward the end of the simulations time. In the case of the co-crystal ligand complex (AQ4-1M17), the RMSD was at around start at 3.5 Å till 90 ns in which it fluctuates to 5.00 Å; this fluctuation is coming from the *C*- and *N*-terminal as can be seen in Figure S1. It is worth mentioning that the 1M17 crystal structure has a tail that was removed prior to MD simulation, as this tail is not related to the active site, which might result in exhibited high fluctuation (Figure 4a).

In the case of the 3RCD complexes, the RMSD of the three complexes was within acceptable limits, with the co-crystal complex (03P-3RCD) being the most stable. All complexes showed an RMSD of less than 3.00 Å; the co-crystal showed an RMSD of 2.00 Å, where the **6j** and **6k** complex’s RMSD was around 3.00 Å, as can be seen in Figure 4b.

**Figure 5:**
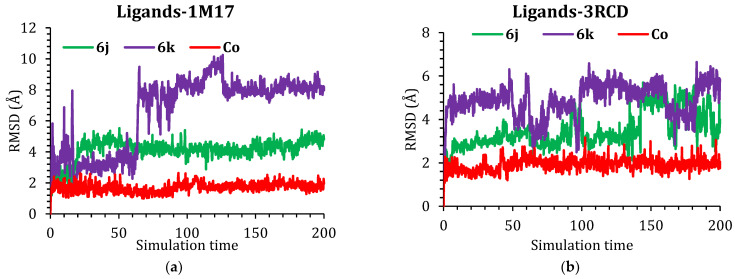
Plots of RMSD for ligand atoms (Å) with respect to the initial structure vs. simulation time (ns) for all the complexes.

The ligand’s RMSDs were also plotted as a function of time with respect to their initial position inside the active site. In the case of the 1M17 protein, compound **6j** showed a fluctuation till around 30 ns, where it moved by 4.00 Å with respect to its initial position; it held this new position toward the end of the simulation time. Compound **6k**, on the other hand, was less stable inside the active site of the 1M17; the compound fluctuated till around 130 ns; at around 140 ns, **6k** reached equilibrium and held a new position which is 9.00 Å far from its original position. The co-crystal showed the most stability with an RMDS of 2.00 Å from the beginning to the end of the simulations. Figure 5a presents the RMSD of ligands inside the active site of 1M17.

In the case of the 3RCD, compound **6j** showed stable behavior inside the active site till around 140 ns of the simulation time with RMSD 3.00 Å; next, the compound was moved by around 5.00 Å with regards to its original position and kept fluctuating till the end of the simulation time. Compound **6k**, on the other hand, showed a fluctuation from the beginning to the end of the simulation in the range of 4.00–5.00 Å with respect to its original position; it was not able to hold position inside the active and continuously changed orientation. The co-crystal ligand was stable inside the active site of the 3CRD and fluctuated in the range of 2.00 Å during the simulation. In addition, Figure 5b presents the RMSD of ligands inside the active site of 3RCD.

**Figure 6:**
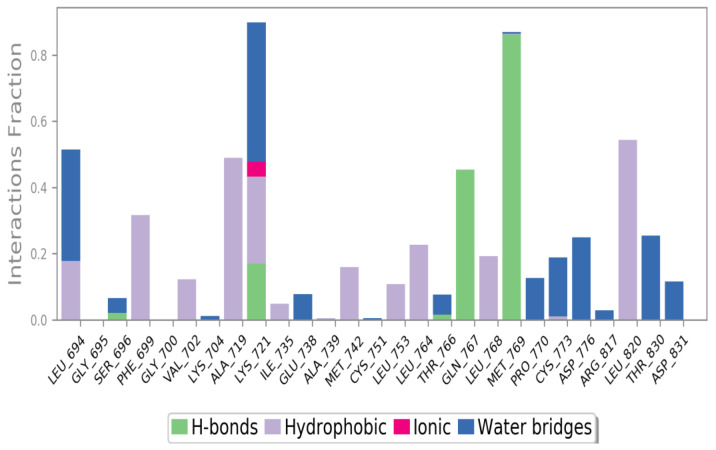
The histogram of **6j**-1M17 contact throughout the trajectory.

**Figure 7:**
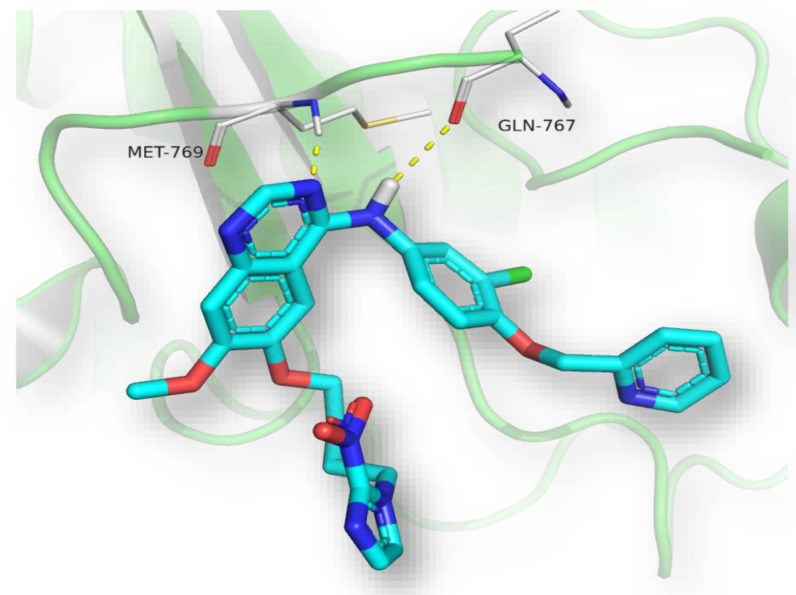
Compound **6j** H-bond interactions with hinge residue in 1M17.

The interactions of **6j** inside the active site of both proteins will be discussed in detail, and the interactions of this ligand with protein residue are plotted using the simulation interaction diagram panel. Compound **6j** (Figure 6) was able to form three H-bond interactions with residues MET769 (85%), GLN767 (50%) with hinge residues (Figure 7), and ALA721 (18%). In addition, compound **6j** was able to interact hydrophobically with LEU820 (50%), ALA719 (50%), and PHE699 (30%), along with H_2_O-bridged H-bond with residues LYS721(40%) and LEU694 (30%). It is worth mentioning that residues with less than 20% interactions were not discussed.

**Figure 8:**
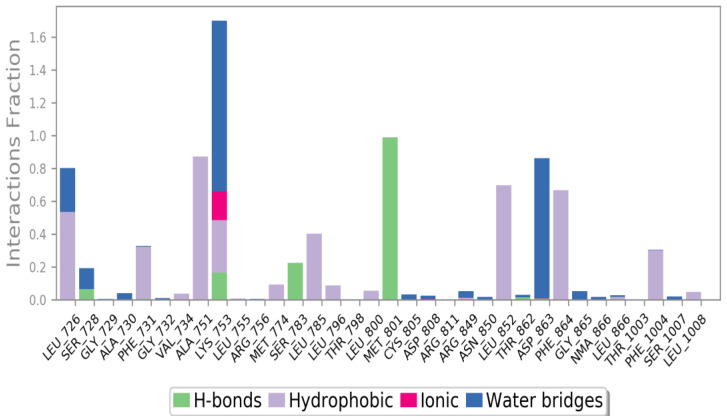
The histogram of **6j**-3RCD contact throughout the trajectory.

**Figure 9:**
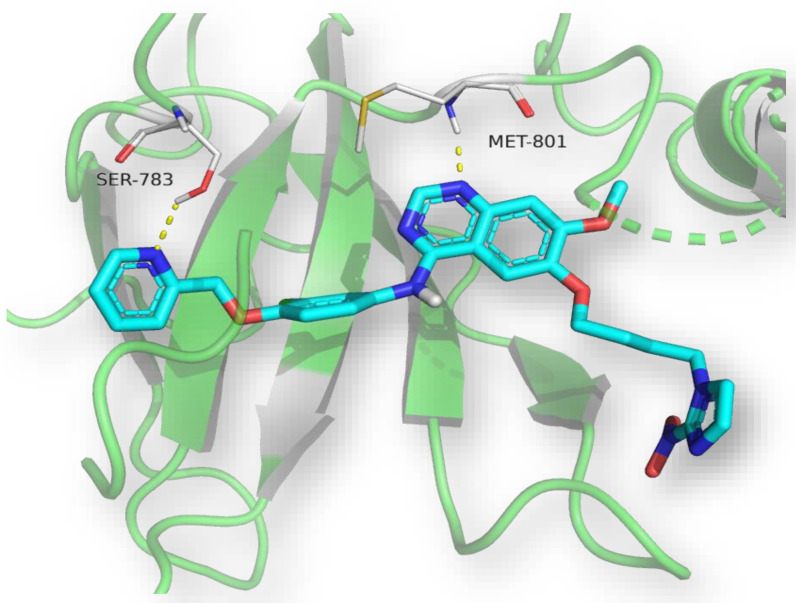
Compound **6j** H-bond interactions with 3RCD residues.

In the case of 3RCD (Figure 8), compound **6j** showed a strong H-bond whit MET801 (100%) interactions followed by SER783 (30%) and LYS753 (18%). Additionally, strong hydrophobic interactions with ALA751 (85%), LEU852 (80%), PHE864 (75%), and LEU726 (55%) were observed. Furthermore, H_2_O-bridged H-bond interactions with LYS753 (85%) and ASP863 (80%), the hydrogen bond interactions are shown in Figure 9. Briefly, MD simulations revealed that compound **6j** might work via inhibition of 1M17 or 3RCD or maybe both. The conformation of the protein changes little to best fit the compound inside the active site, with the hinge playing a critical role in this conformational change (Figure S2).

#### 3.4.6. MM-GBSA Study

The average MM-GBSA binding energy over the last 50 ns was generated using the thermal_mmgbsa.py python script provided by Schrödinger, which also generates Coulomb energy, Covalent binding energy, Van der Waals energy, Lipophilic energy, Generalized Born electrostatic solvation energy, Hydrogen-bonding energy. All the obtained data are shown in Table 8.

From the MM-GBSA calculations, the most favored binding was exerted by **6j** in both protein active sites (Table 8); this came from the fact that compound **6j** was able to orient itself inside the active site to fit the best. Therefore, MM-GBSA results reveal that compound **6j** showed the highest binding energy in both systems, with around 4 kcal/mol and 27 kcal/mol difference compared to co-crystal in the case of 1M17 and 3RCD, respectively.

## 4. Conclusions

As a step toward the development of novel small molecules with EGFR/HER2 inhibition properties, lapatinib derivatives (**6a**–**l**) were synthesized and biologically screened against both kinases. At a single dose concentration of 10 µM, compounds **6i**–**l**, which have the 3-chloro-4-(pyridin-2-ylmethoxy)aniline moiety, exhibited exceptional ranges of % inhibition over EGFR (97.65–99.03%) and HER2 (87.16–96.73%). Lapatinib derivative **6j** demonstrated an IC_50_ value of 1.8 nM over EGFR and 87.8 nM over HER2. Regarding potency over EGFR, compound **6j** showed 50-fold higher potency than staurosporine and 6-fold compared to lapatinib. A small kinase selectivity panel of compound **6j** revealed almost no inhibitory activity over CDK2/cyclin A, c-MET, FGFR1, KDR/VEGFR2, and P38a/MAPK14, respectively. The molecular docking and molecular dynamics shed light on the binding modes of the target small molecules. As a result, compound **6j** was recognized as a very effective dual EGFR/HER2 inhibitor deserving of additional research.

## Figures and Tables

**Table 1:** Chemical structures and yields of the newly synthesized lapatinib derivatives **6a**–**l**.

Comp.	Chemical Structure	Isolated Yield (%)
**6a**	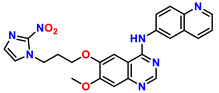	50.3
**6b**	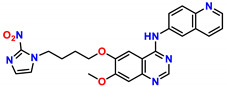	83.6
**6c**	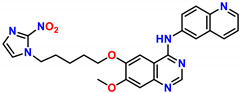	69.1
**6d**	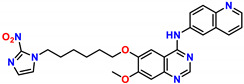	70.0
**6e**	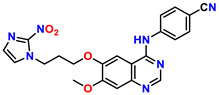	43.7
**6f**	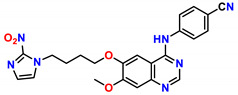	61.4
**6g**	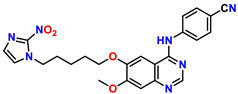	81.7
**6h**	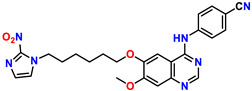	69.9
**6i**	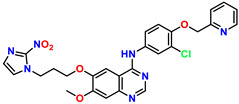	55.9
**6j**	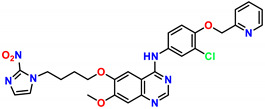	83.9
**6k**	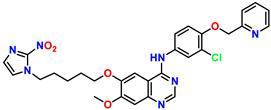	76.5
**6l**	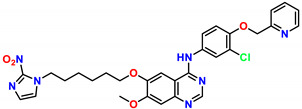	79.8

**Table 2:** Physicochemical properties, pharmacokinetics, and lead likeness studies of the designed candidates **6a**–**l**.

		6a	6b	6c	6d	6e	6f	6g	6h	6i	6j	6k	6l
**Physicochemical properties**	**Molar Refractivity**	132.37	137.18	141.89	146.79	121.78	126.59	131.40	136.21	150.85	155.66	160.47	165.27
	**TPSA (Å^2^)**	132.80				143.70				142.03			
	**Log *P* *o/w* (WLOGP)**	4.50	4.89	5.28	5.67	3.83	4.22	4.61	5.00	5.43	5.82	6.21	6.60
	**Consensus Log *P* *o/w***	2.82	3.16	3.47	3.81	2.36	2.74	3.00	3.23	3.55	3.86	4.24	4.60
	**Water solubility**	Moderately Soluble		Poorly Soluble								Insoluble	
**Pharmacokinetics**	**GI absorption**	Low											
	**BBB permeant**	No											
	**P-gp substrate**	Yes		No									
	**CYP1A2 inhibitor**	Yes					No						
	**CYP3A4 inhibitor**	Yes											
	**CYP2C9 inhibitor**	Yes											
	**CYP2C19 inhibitor**	Yes											
	**CYP2D6 inhibitor**	Yes									No		
**Lead likeness**	**Drug likeness (Lipinski)**	Yes								No			

**Table 3:** % Inhibition values of the synthesized lapatinib derivatives **6a**–**l** over EGFR and HER2 kinases at 10 µM.

Cpd	% Kinase Inhibition at 10 µM	
	EGFR	HER2
**6a**	98.79	74.72
**6b**	99.34	78.04
**6c**	99.32	89.99
**6d**	99.09	79.68
**6e**	67.27	8.78
**6f**	76.55	11.69
**6g**	91.80	43.06
**6h**	94.67	47.34
**6i**	97.65	87.16
**6j**	99.03	96.73
**6k**	98.64	91.16
**6l**	98.58	89.05

**Table 4:** IC_50_ values (µM) exhibited by compound **6j** and staurosporine on EGFR and HER2.

Cpd	Kinase Assay	
	EGFR IC_50_ (nM)	HER2 IC_50_ (nM)
**6j**	1.8	87.8
**Staurosporine**	88.1	35.5
**Lapatinib**	10.0	9.0

**Table 5:** % Inhibition values of the most active compound **6j** over CDK2, c-Met, FGFR1, VEGFR2, and P38a at 10 µM concentration.

Cpd	% Kinase Inhibition at 10 µM				
	CDK2/cyclin A	c-MET	FGFR1	KDR/VEGFR2	P38a/MAPK14
**6j**	–0.83	19.27	13.87	10.32	–33.31

**Table 6:** 3D binding interactions and positioning of the co-crystallized AQ4 inhibitor, **6j**, and **6k** candidates within the EGFR (PDB ID: 1M17) binding pocket.

3D Interactions	3D Pocket Positioning
Co-Crystallized AQ4 Inhibitor	
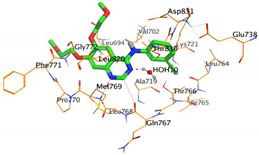	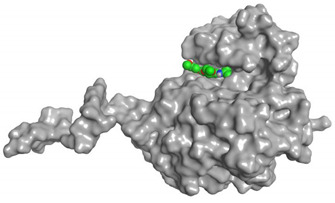
**6j**	
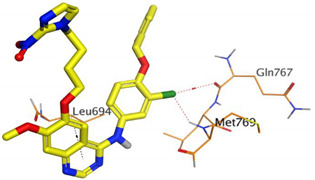	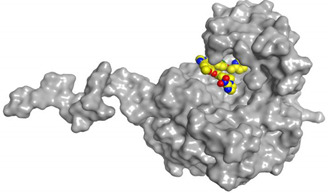
**6k**	
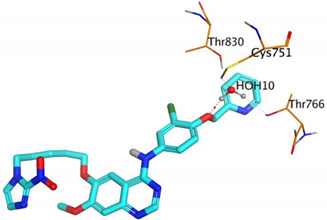	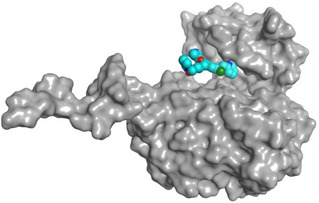

**Table 7:** 3D binding interactions and positioning of the co-crystallized AQ4 inhibitor, **6j**, and **6k** candidates within the HER2 (PDB ID: 3RCD) binding pocket.

3D Interactions	3D Pocket Positioning
Co-Crystallized 03P Inhibitor	
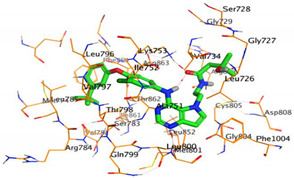	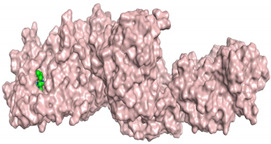
**6j**	
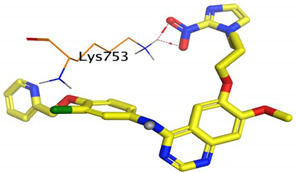	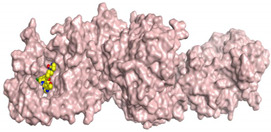
**6k**	
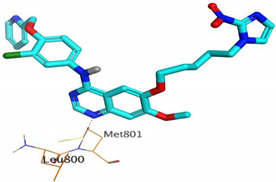	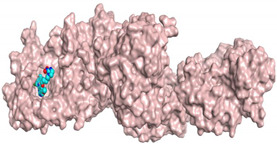

**Table 8:** Prime MM-GBSA energies for Ligands binding at the active sites of both EGFR (PDB ID: 1M17) and HER2 (PDB ID: 3RCD) target receptors.

	ΔG Binding	Coulomb	Covalent	H-Bond	Lipo	Solv_GB	vdW
**6j-1m17**	−67.76	−3.38	0.40	−1.09	−18.50	14.09	−58.88
**6k-1m17**	−57.51	2.03	2.48	−0.09	−18.07	11.43	−54.62
**AQ4-1M17**	−63.27	−15.75	1.91	−0.57	−18.90	25.98	−55.93
**6j-3RCD**	−102.57	−6.71	5.25	−0.97	−34.49	15.59	−79.99
**6k-3RCD**	−93.50	−4.02	4.26	−0.63	−31.98	18.14	−77.68
**03P-3RCD**	−75.81	−10.36	0.63	−0.84	−21.37	24.94	−68.51

Coulomb: Coulomb energy; Covalent: Covalent binding energy; vdW: Van der Waals energy; Lipo: Lipophilic energy; Solv_GB: Generalized Born electrostatic solvation energy; H-bond: Hydrogen-bonding energy.

## Data Availability

Data is contained within the article and supplementary material.

## Supplementary Materials

The following are available online at https://www.mdpi.com/article/10.3390/ph16010043/s1, the MD methodologies (SI 1 and SI 2), ^1^H NMR, ^13^C NMR, purity, and HRMS data of the lapatinib derivatives (**6a**–**l**). References [55,57,58,59,60,61,62] are cited in the supplementary materials. Figure S1: ^1^H NMR and ^13^C NMR spectrum of compound **6a**, Figure S2: HRMS chart of compound **6a**, Figure S3: HPLC purity chart of compound **6a**, Figure S4: ^1^H NMR and ^13^C NMR spectrum of compound **6b**, Figure S5: HRMS chart of compound **6b**, Figure S6: HPLC purity chart of compound **6b**, Figure S7: ^1^H NMR and ^13^C NMR spectrum of compound **6c**, Figure S8: HRMS chart of compound **6c**, Figure S9: HPLC purity chart of compound **6c**, Figure S10: ^1^H NMR and ^13^C NMR spectrum of compound **6d**, Figure S11: HRMS chart of compound **6d**, Figure S12: HPLC purity chart of compound **6d**, Figure S13: ^1^H NMR and ^13^C NMR spectrum of compound **6e**, Figure S14: HRMS chart of compound **6e**, Figure S15: HPLC purity chart of compound **6e**, Figure S16: ^1^H NMR and ^13^C NMR spectrum of compound **6f**, Figure S17: HRMS chart of compound **6f**, Figure S18: HPLC purity chart of compound **6f**, Figure S19: ^1^H NMR and ^13^C NMR spectrum of compound **6g**, Figure S20: HRMS chart of compound **6g**, Figure S21: HPLC purity chart of compound **6g**, Figure S22: ^1^H NMR and ^13^C NMR spectrum of compound **6h**, Figure S23: HRMS chart of compound **6h**, Figure S24: HPLC purity chart of compound **6h**, Figure S25: ^1^H NMR and ^13^C NMR spectrum of compound **6i**, Figure S26: HRMS chart of compound **6i**, Figure S27: HPLC purity chart of compound **6i**, Figure S28: ^1^H NMR and ^13^C NMR spectrum of compound **6j**, Figure S29: HRMS chart of compound **6j**, Figure S30: HPLC purity chart of compound **6j**, Figure S31: ^1^H NMR and ^13^C NMR spectrum of compound **6k**, Figure S32: HRMS chart of compound **6k**, Figure S33: HPLC purity chart of compound **6k**, Figure S34: ^1^H NMR and ^13^C NMR spectrum of compound **6l**, Figure S35: HRMS chart of compound **6l**, Figure S36: HPLC purity chart of compound **6l**.
