# Supplementary material for: Design, Synthesis, Biological Evaluation, and Molecular Dynamics Studies of Novel Lapatinib Derivatives
Source: Pharmaceuticals (Basel). 2022 Dec 28;16(1):43. doi: 10.3390/ph16010043 (PMC9862743; doi:10.3390/ph16010043)
Supplement: Supplementary file 1 [file pharmaceuticals-16-00043-s001.zip › pharmaceuticals-2056307-supplementary.pdf]

Supplementary material

Design, Synthesis, Biological Evaluation and Molecular Dynamics Studies of Novel Lapatinib Derivatives

SI 1. Molecular dynamics simulations

The MD simulations were carried out using Desmond simulation package of Schrödinger LLC [1]. The NPT ensemble with the temperature 300 K and a pressure 1 bar was applied in all runs. The simulation length was 200 ns with a relaxation time 1 ps for the ligands. The OPLS3 force field parameters were used in all simulations [2]. The cutoff radius in Coulomb interactions was 9.0 Å. The orthorhombic periodic box boundaries were set 10 Å away from the protein atoms. The water molecules were explicitly described using the transferable intermolecular potential with three points (TIP3P) model [3,4]. Salt concentration set to 0.15 M NaCl and was built using the System Builder utility of Desmond [5]. The Martyna−Tuckerman−Klein chain coupling scheme with a coupling constant of 2.0 ps was used for the pressure control and the Nosé−Hoover chain coupling scheme for the temperature control [6,7]. Nonbonded forces were calculated using a RESPA integrator where the short-range forces were updated every step and the long-range forces were updated every three steps. The trajectories were saved at 20 ns intervals for analysis. The behavior and interactions between the ligands and protein were analyzed using the Simulation Interaction Diagram tool implemented in Desmond MD package. The stability of MD simulations was monitored by looking on the RMSD of the ligand and protein atom positions in time.

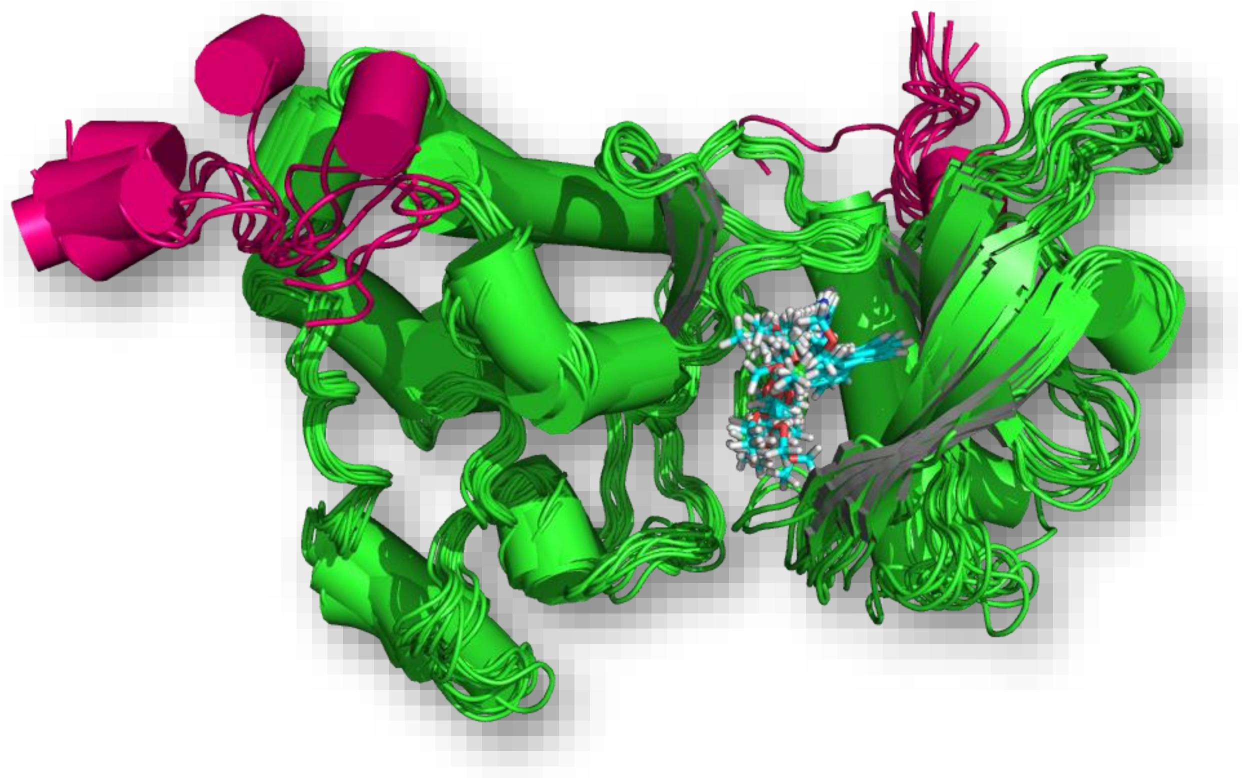

Figure SI 1. Snapshots of Co-1M17 displaying the *C*- and *N*-terminal fluctuations in red.

SI 2. MD trajectory analysis and prime MM-GBSA calculations

Simulation interactions diagram panel of Maestro software was used to monitoring interactions contribution in the ligand-protein stability. The molecular mechanics generalized born/solvent accessibility (MM – GBSA) was performed to calculate the ligand binding free energies and ligand strain energies for docked compounds over the last 50 ns with thermal_mmgbsa.py python script provided by Schrodinger which takes a Desmond trajectory file, splits it into individual snapshots, runs the MM-GBSA calculations on each frame, and outputs the average computed binding energy.

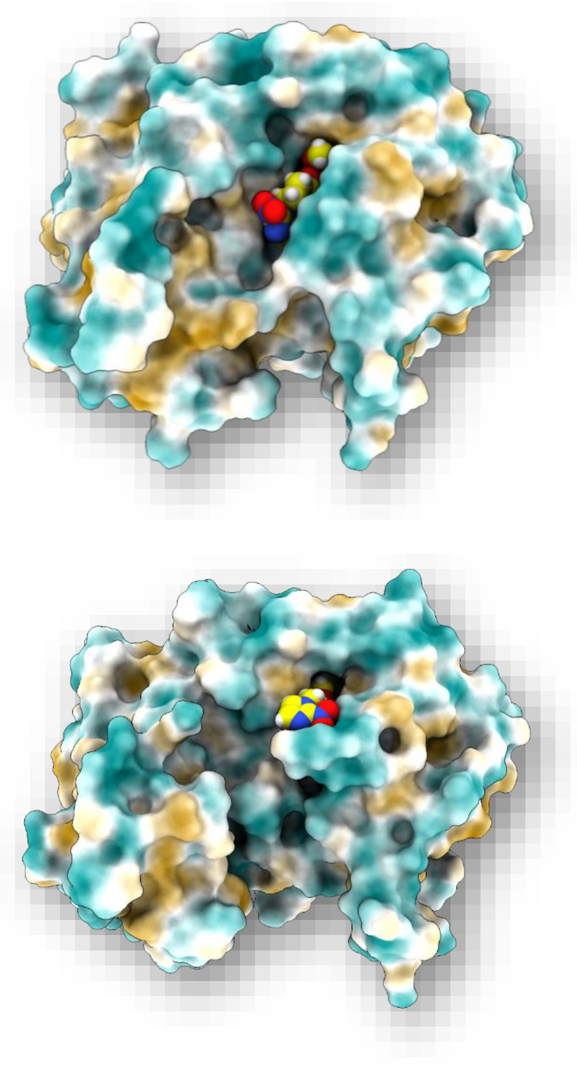

Figure SI 2. The position of **6j** inside the active site 3RCD.

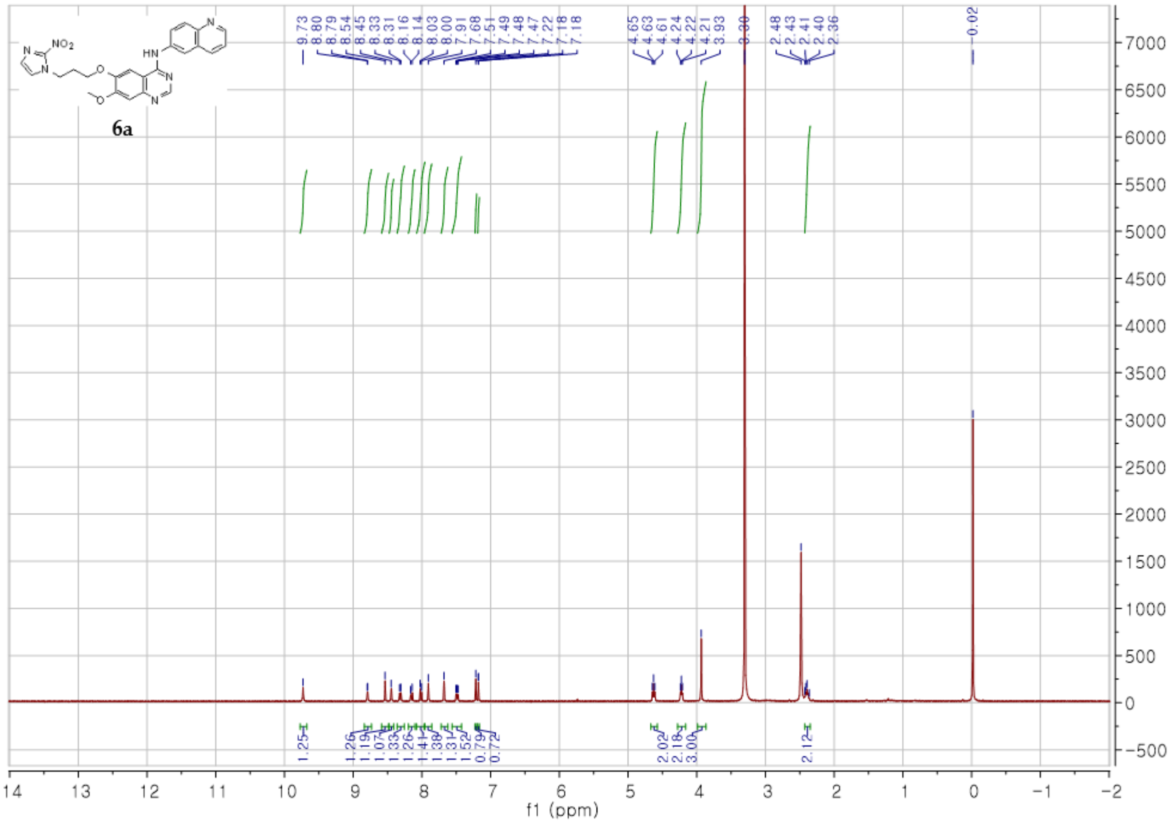

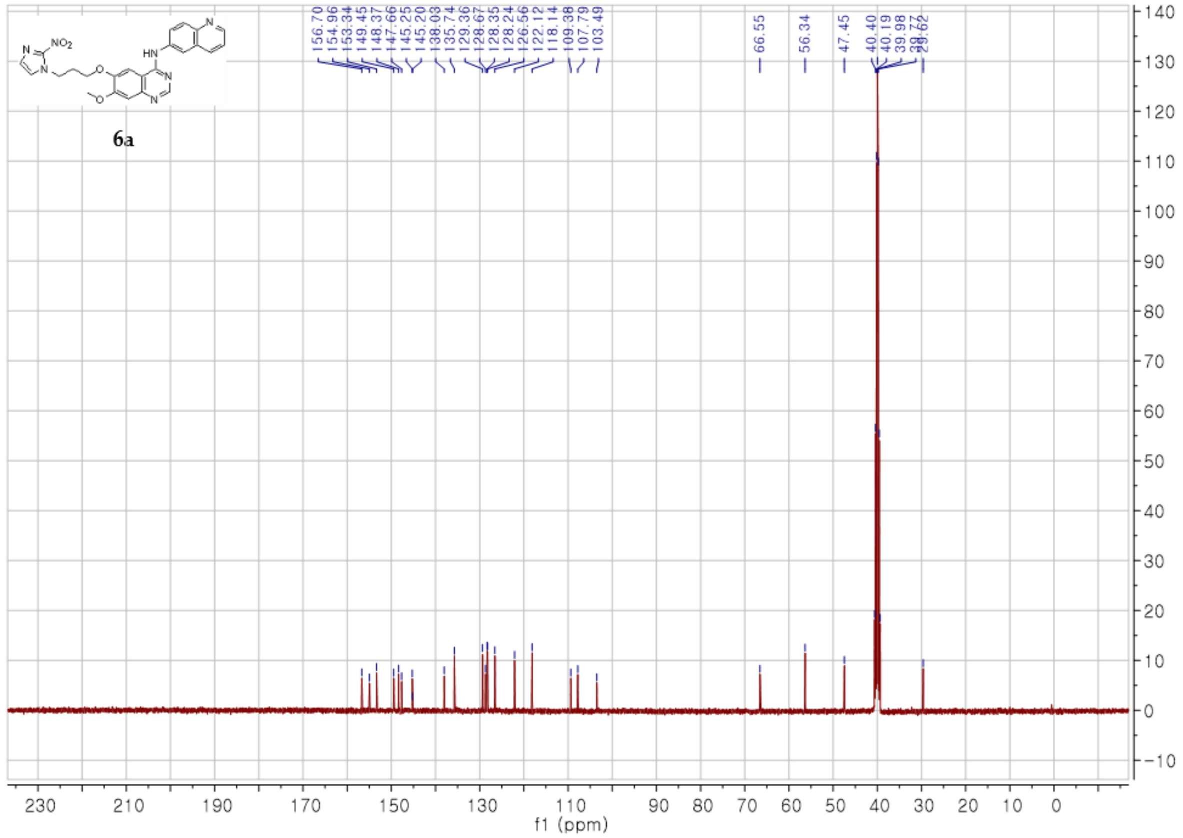

Figure S1. ^{1}H NMR and ^{13}C NMR spectrum of compound **6a**

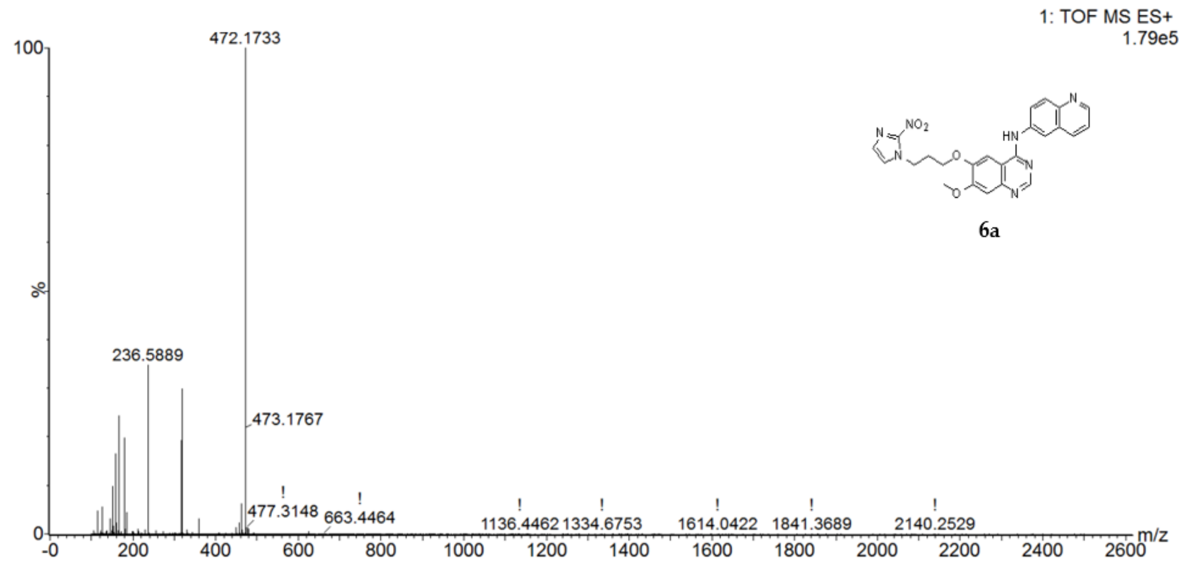

Figure S2. HRMS chart of compound **6a**

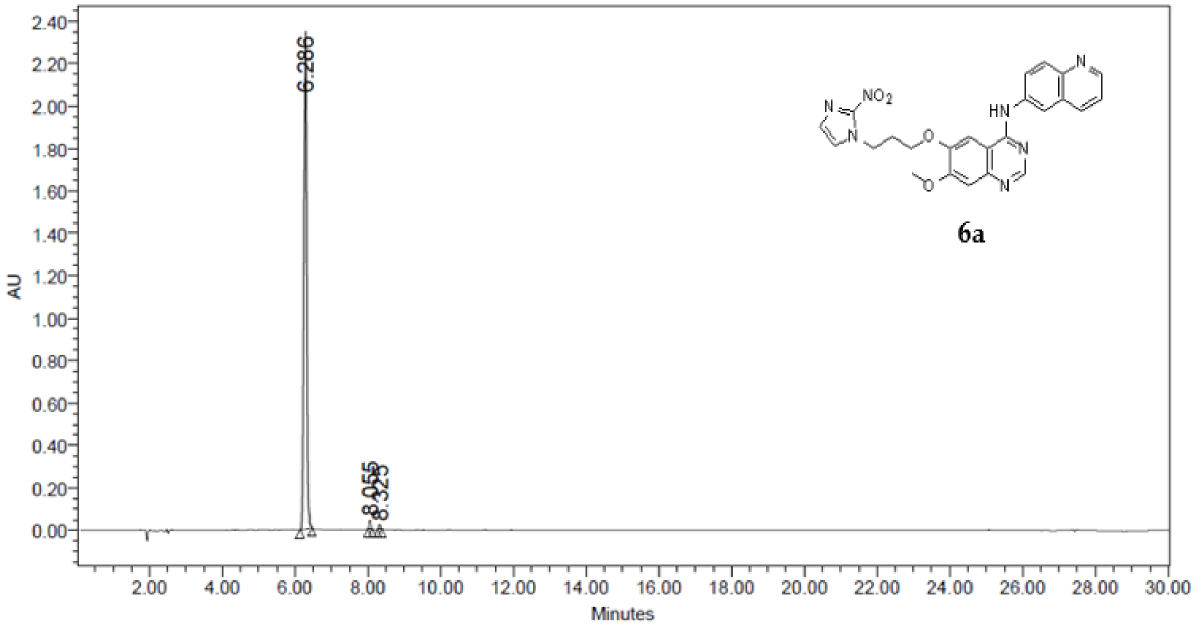

	RT	Area	% Area	Height
1	6.286	12926598	98.30	2324515
2	8.055	143560	1.09	37861
3	8.325	80112	0.61	20826

Figure S3. HPLC purity chart of compound **6a**

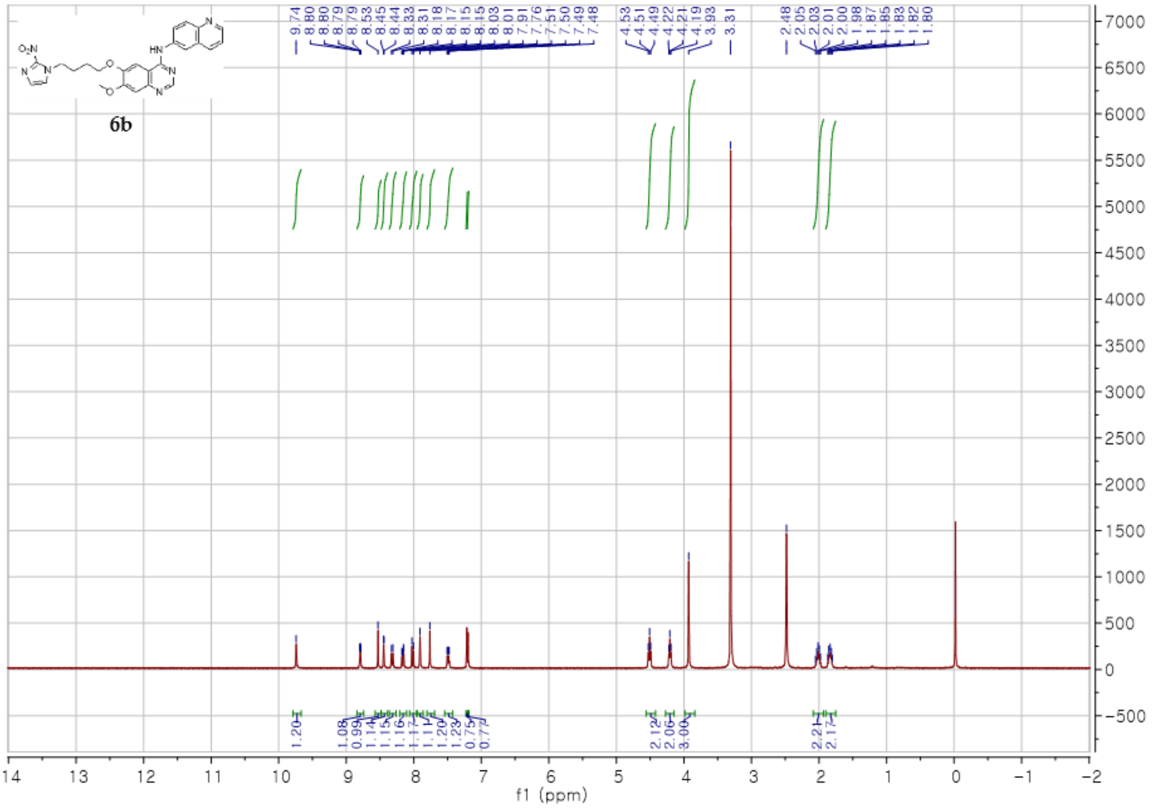

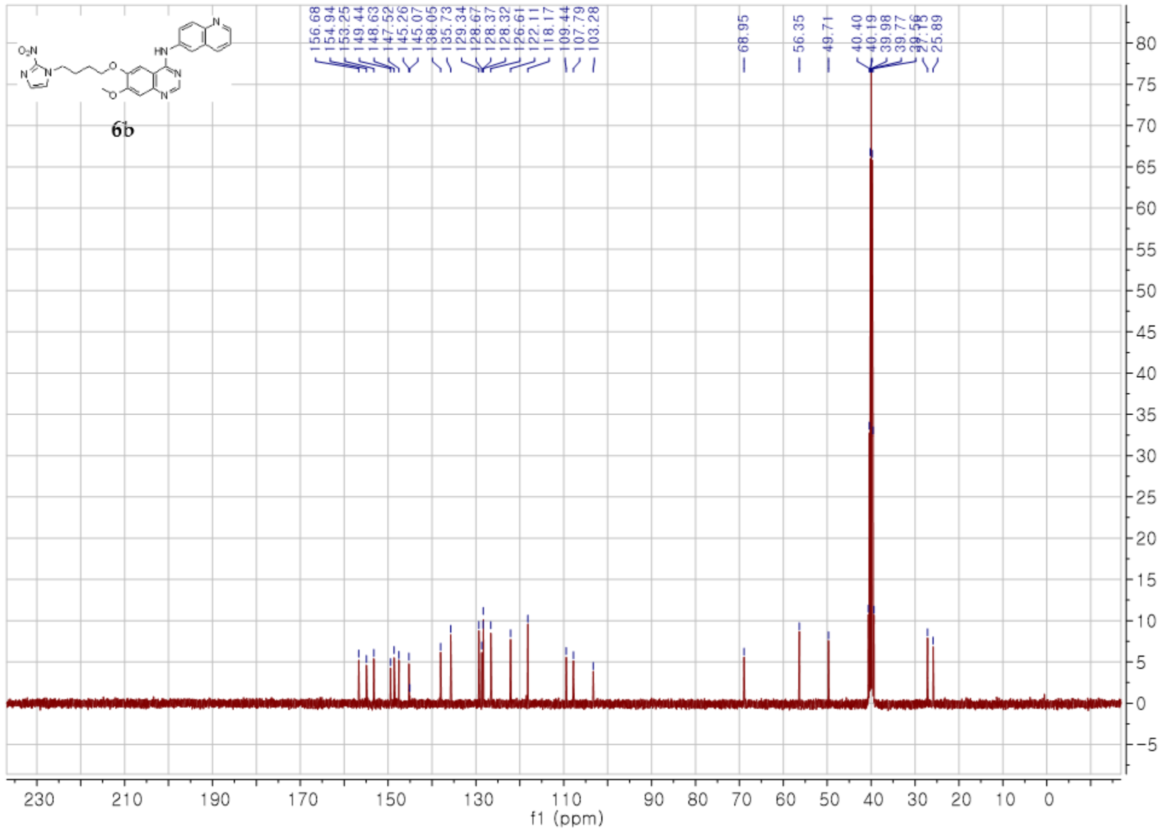

Figure S4. ^{1}H NMR and ^{13}C NMR spectrum of compound **6b**

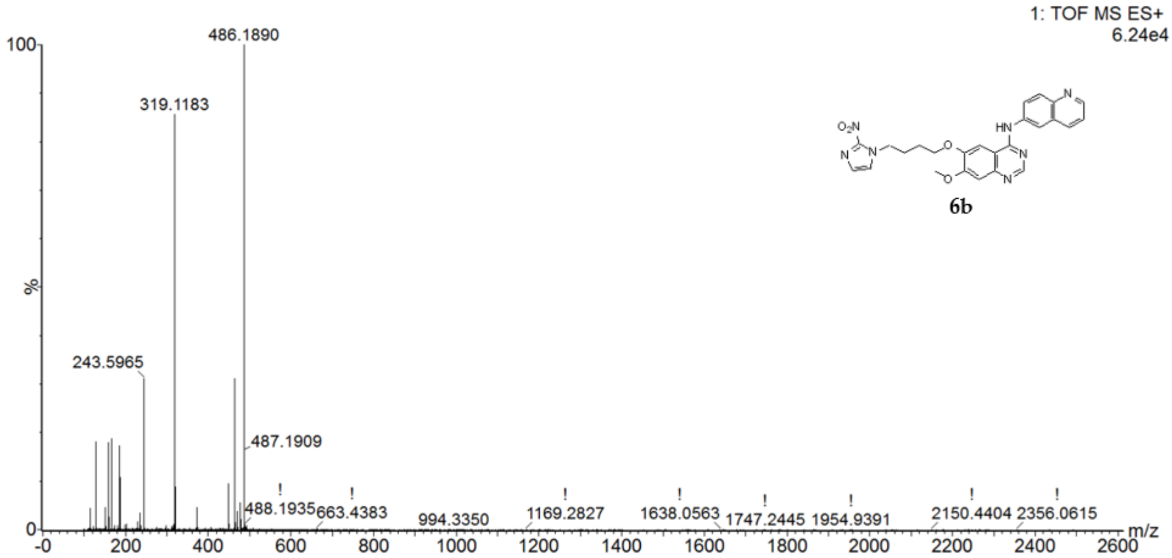

Figure S5. HRMS chart of compound **6b**

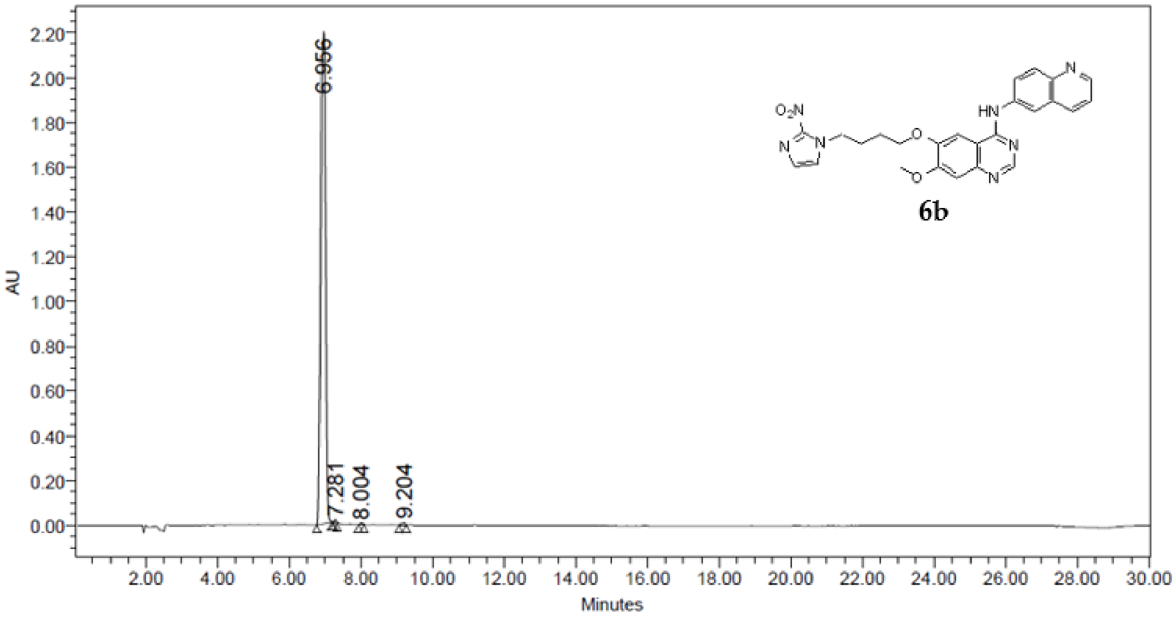

	RT	Area	% Area	Height
1	6.956	20875908	99.41	2229635
2	7.281	36643	0.17	10673
3	8.004	24590	0.12	4211
4	9.204	63719	0.30	9107

Figure S6. HPLC purity chart of compound **6b**

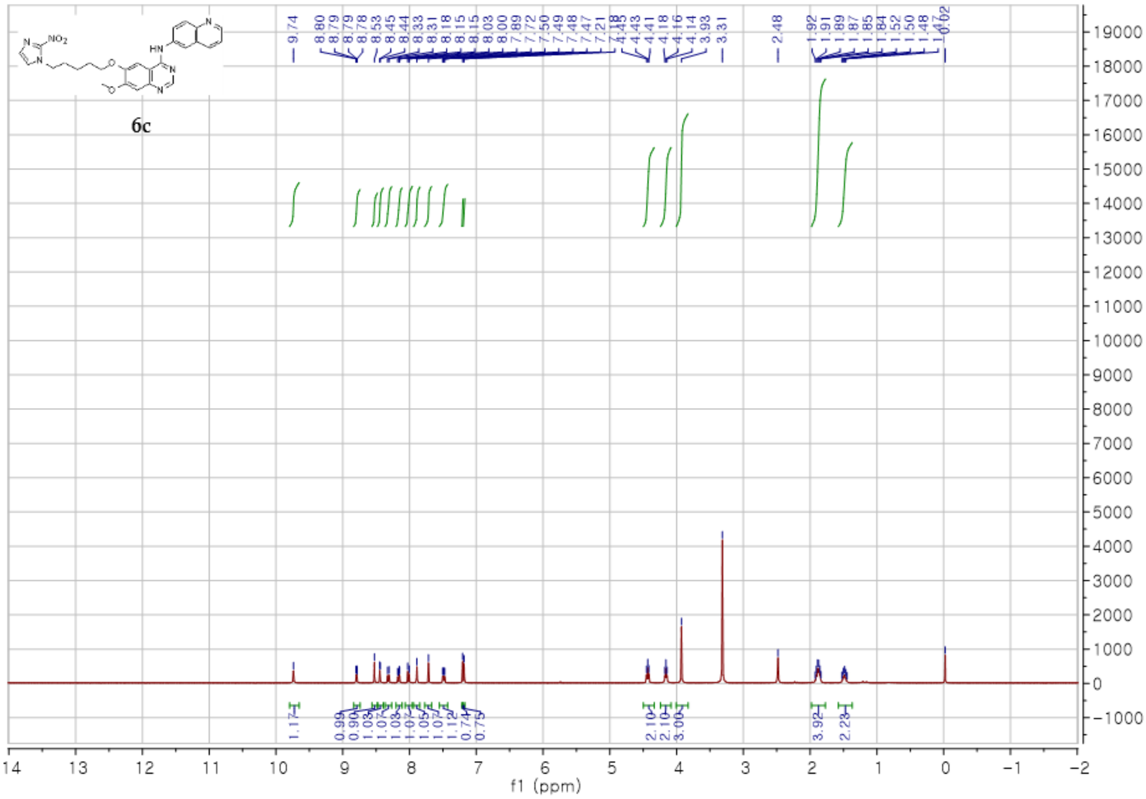

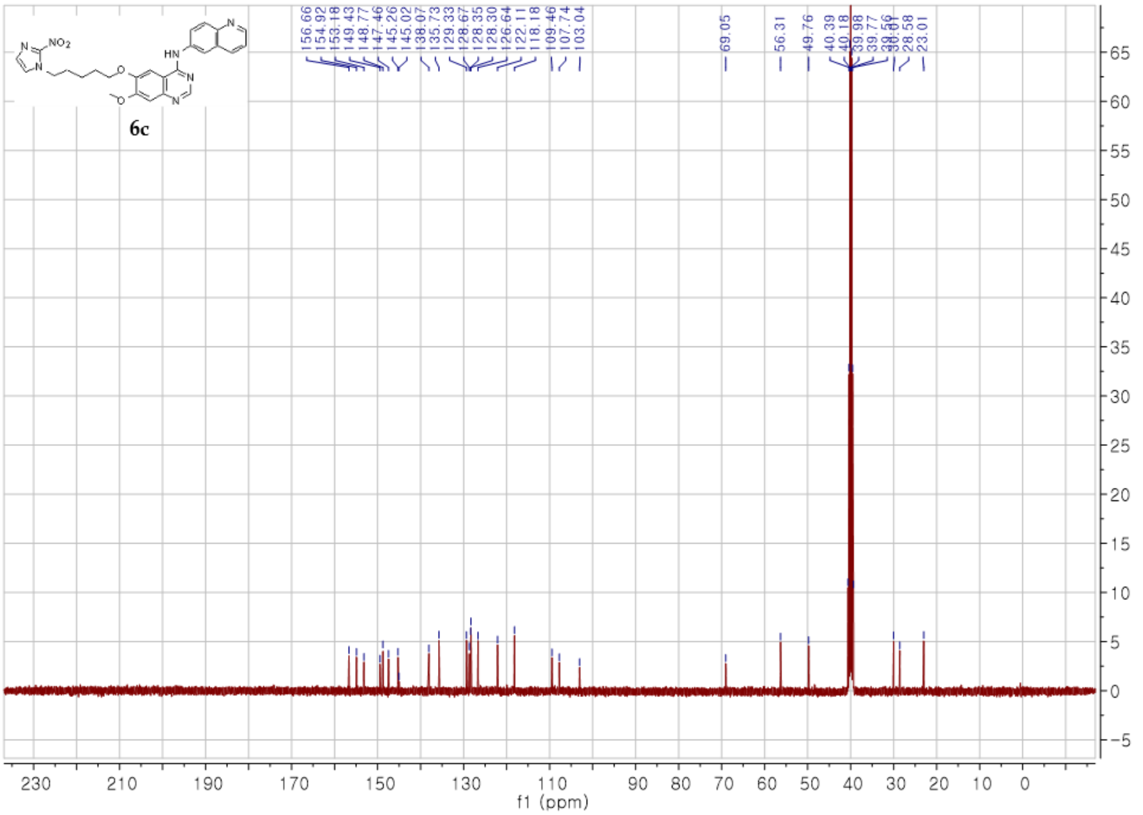

Figure S7. ^{1}H NMR and ^{13}C NMR spectrum of compound **6c**

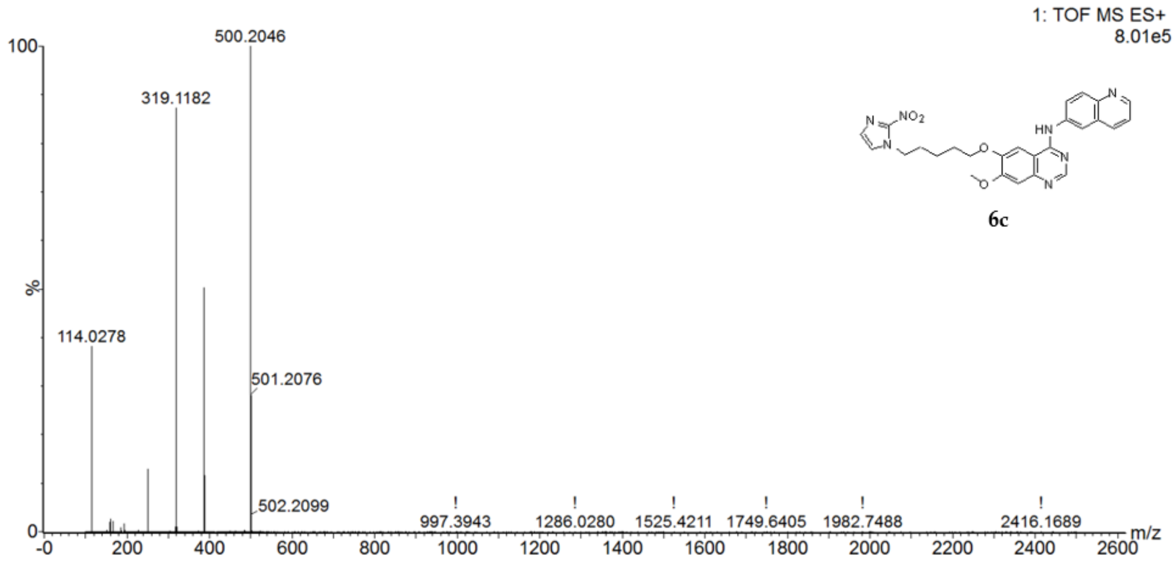

Figure S8. HRMS chart of compound **6c**

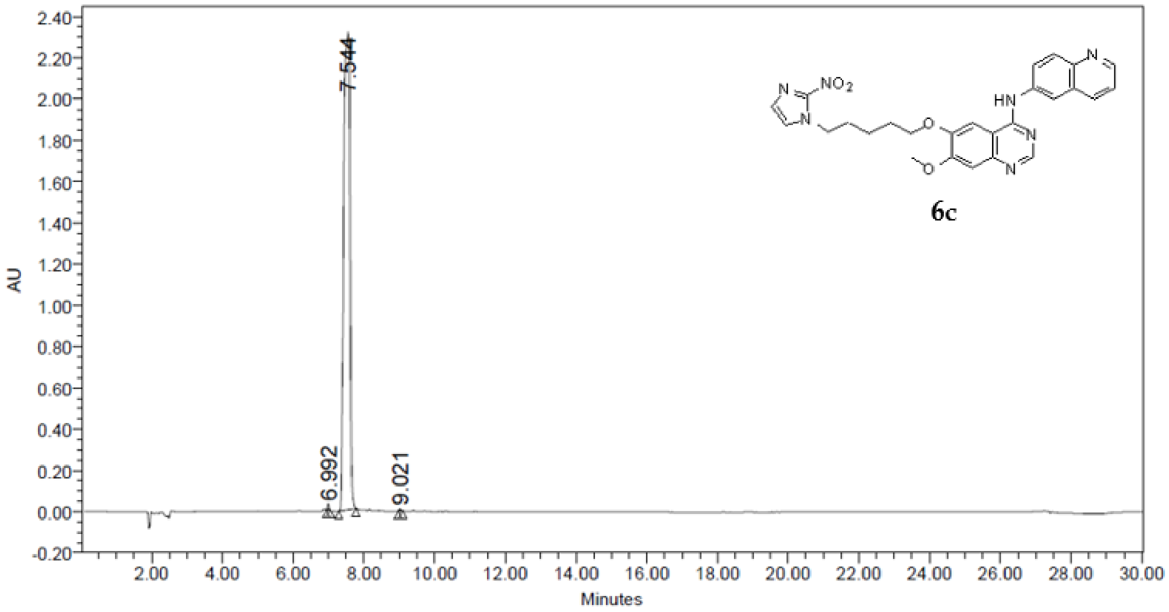

	RT	Area	% Area	Height
1	6.992	90562	0.33	26831
2	7.544	27469220	99.55	2350293
3	9.021	32992	0.12	8194

Figure S9. HPLC purity chart of compound **6c**

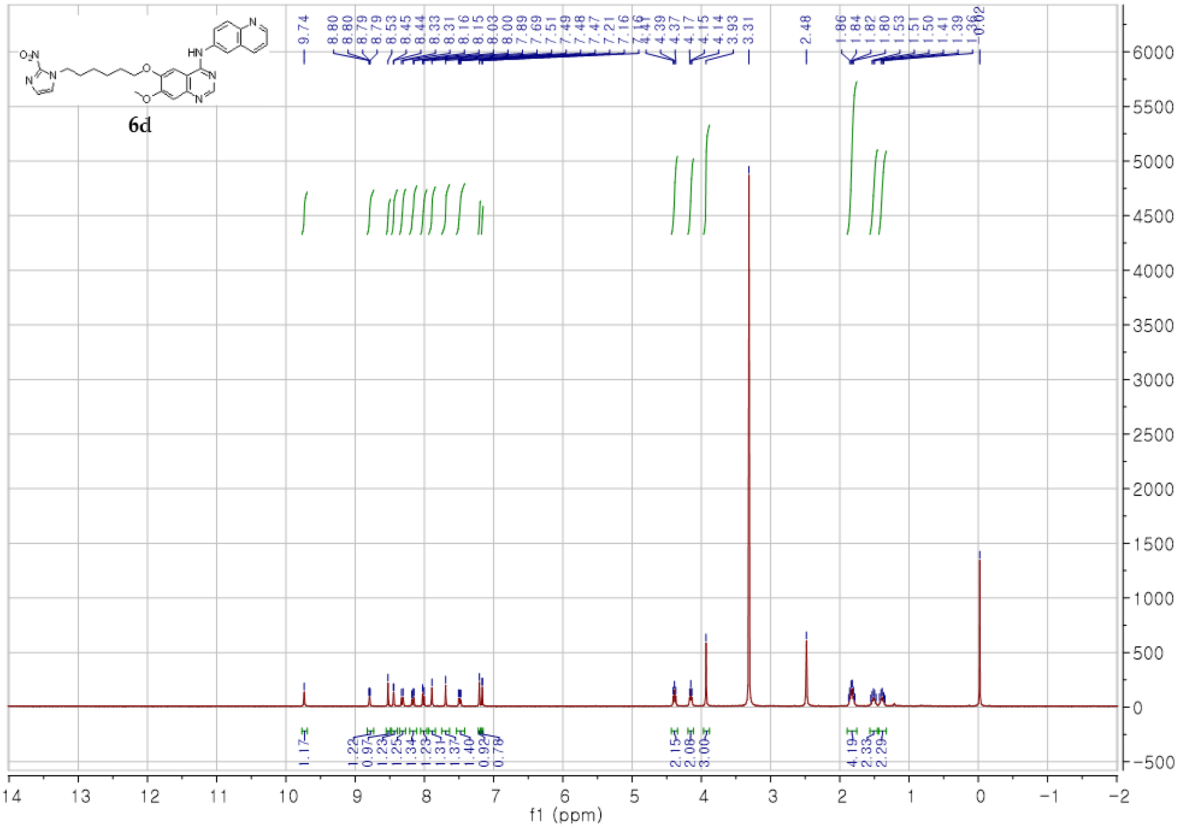

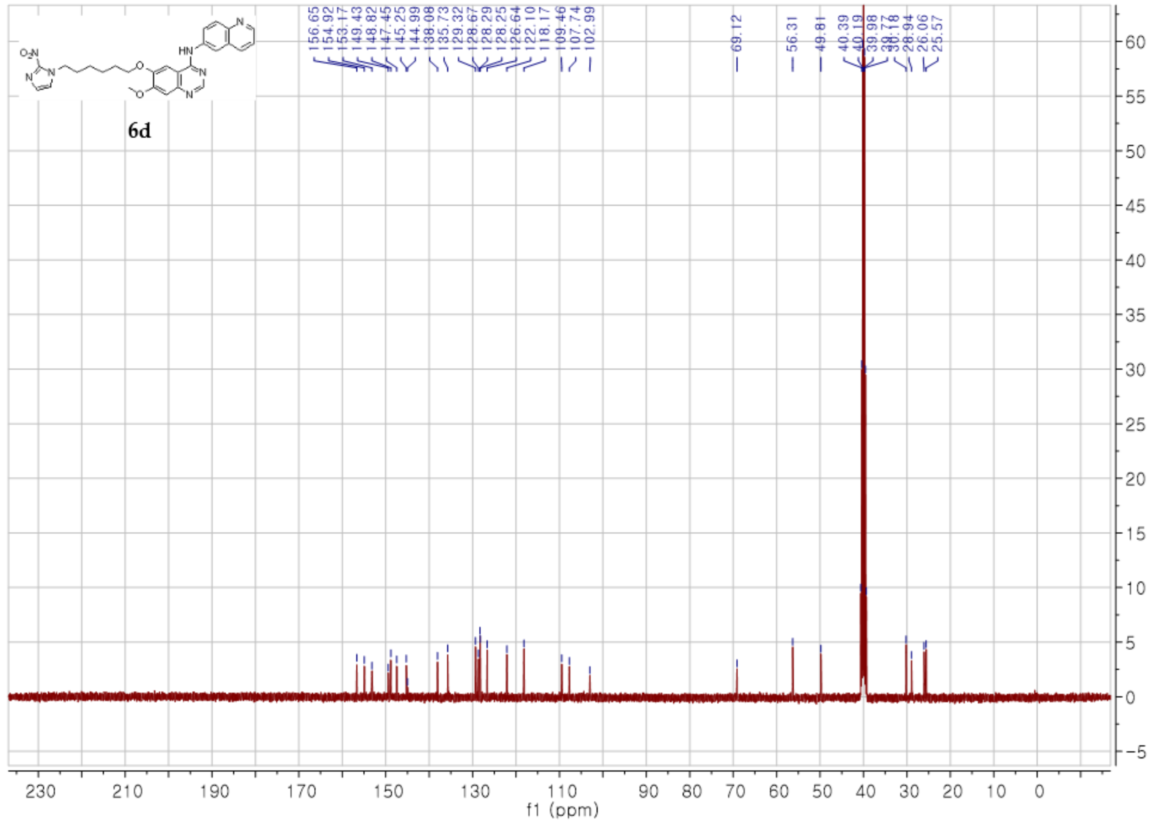

Figure S10. ^{1}H NMR and ^{13}C NMR spectrum of compound **6d**

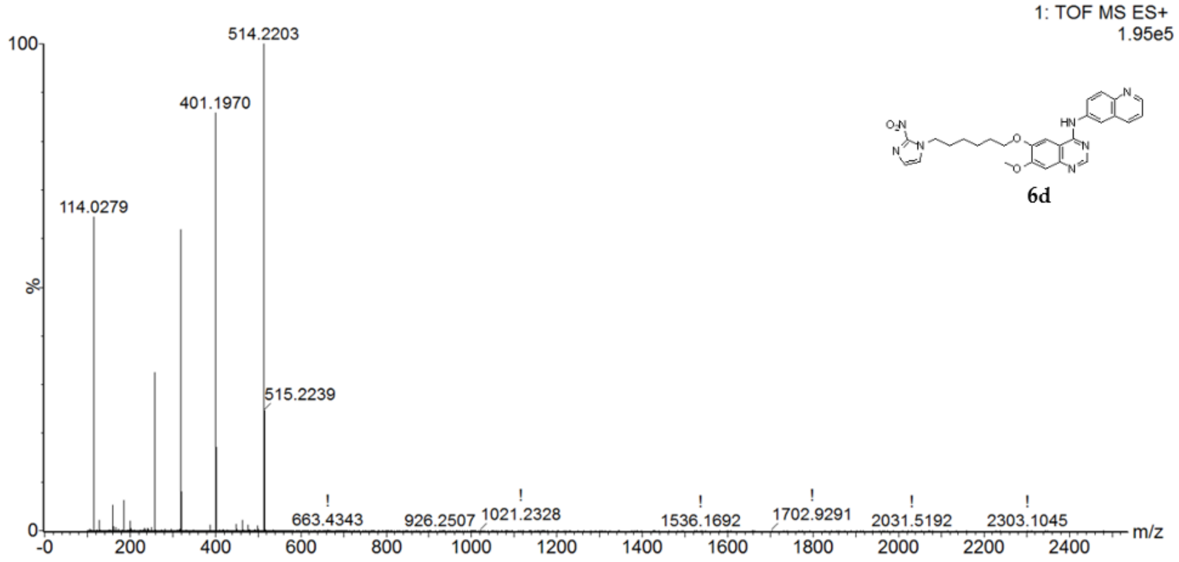

Figure S11. HRMS chart of compound **6d**

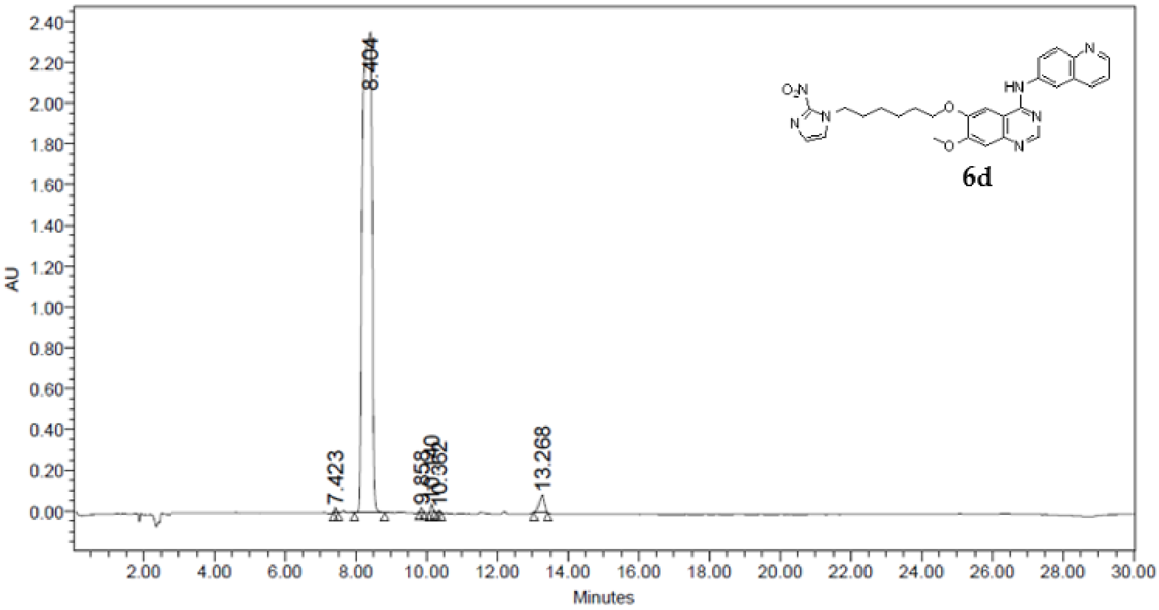

	RT	Area	% Area	Height
1	7.423	80968	0.18	19564
2	8.404	42960318	97.14	2406479
3	9.858	92248	0.21	17951
4	10.140	171871	0.39	37151
5	10.362	43906	0.10	11372
6	13.268	877109	1.98	84331

Figure S12. HPLC purity chart of compound **6d**

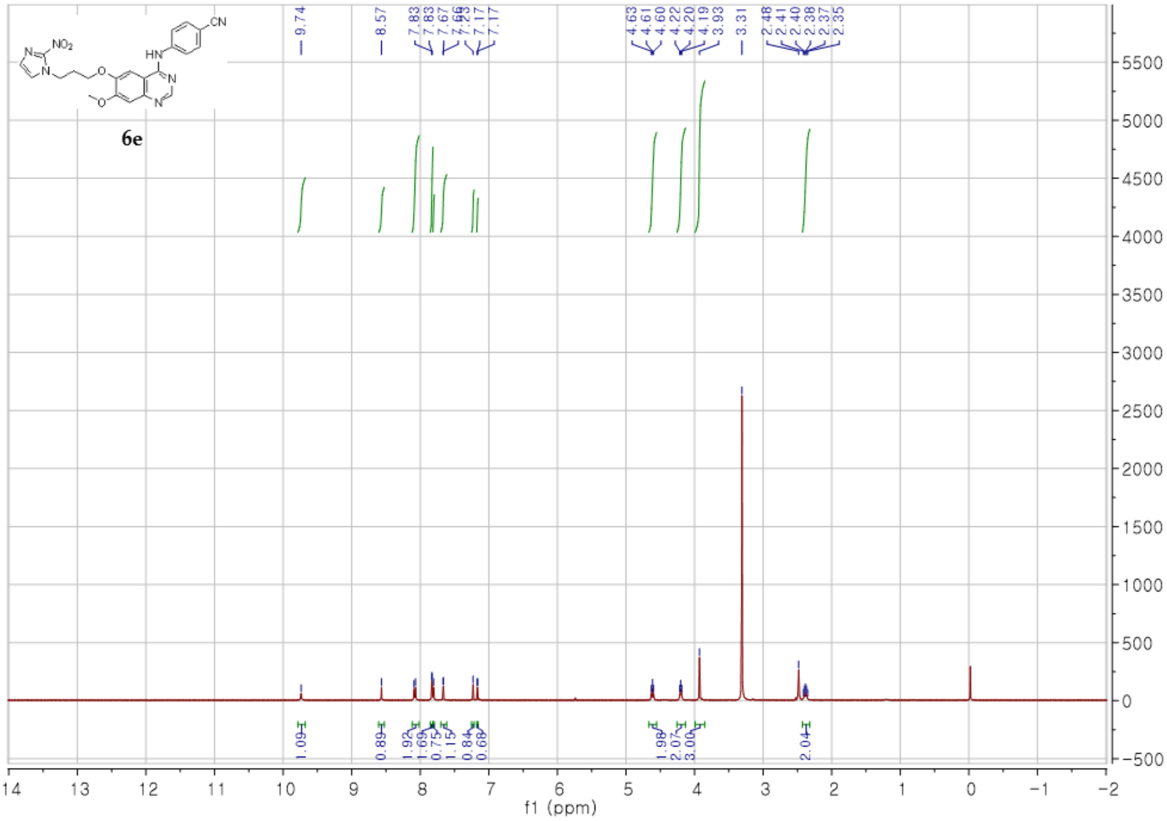
6e
9.74
8.57
7.83
7.83
7.67
7.66
7.23
7.17
7.17
4.63
4.61
4.60
4.22
4.20
4.19
3.93
3.31
2.48
2.41
2.40
2.38
2.37
2.35
1.09
0.89
1.92
1.69
0.75
1.15
0.84
0.68
1.98
2.07
3.00
2.04
f1 (ppm)

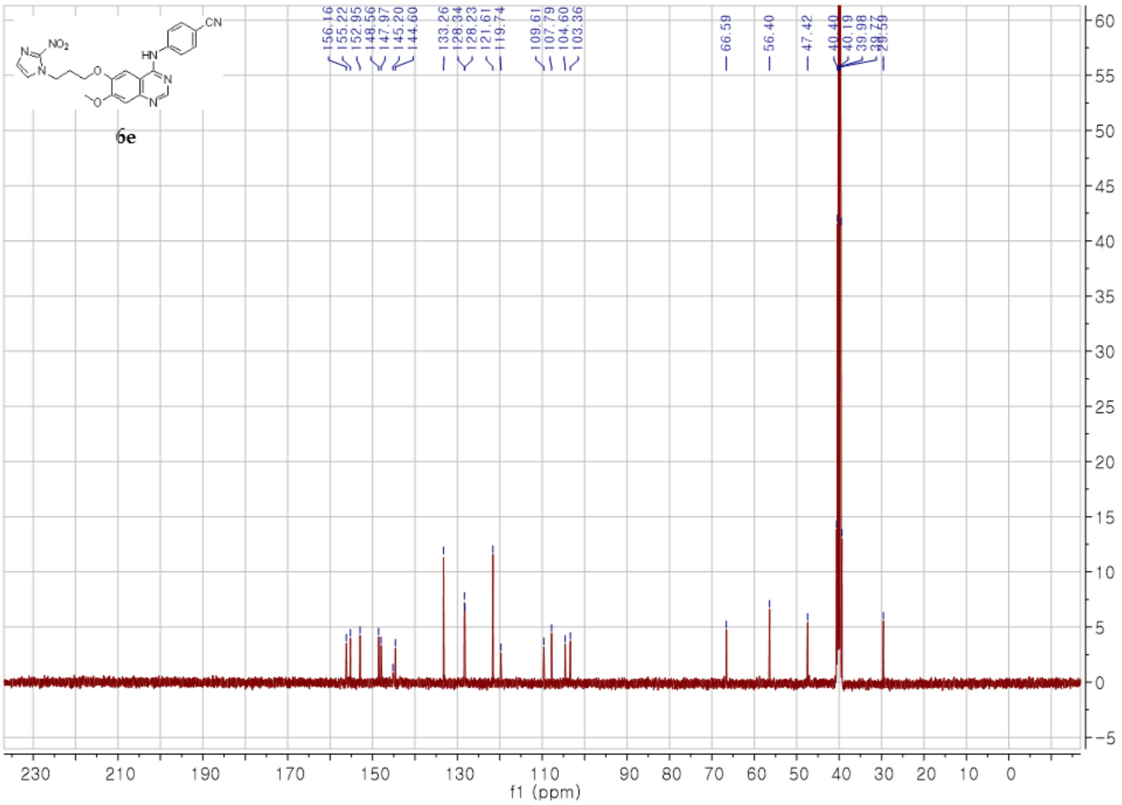
6e
156.16
155.22
152.95
148.56
147.97
145.20
144.60
133.26
128.34
128.23
121.61
119.74
109.61
107.79
104.60
103.36
66.59
56.40
47.42
40.40
40.19
39.98
39.77
29.59
f1 (ppm)

Figure S13. ^{1}H NMR and ^{13}C NMR spectrum of compound **6e**

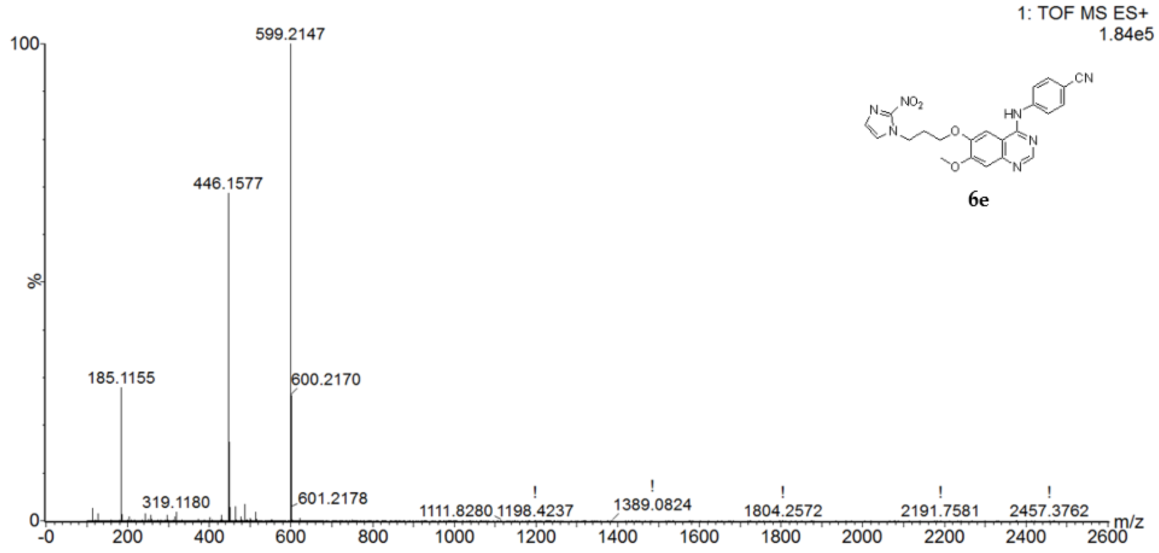

Figure S14. HRMS chart of compound **6e**

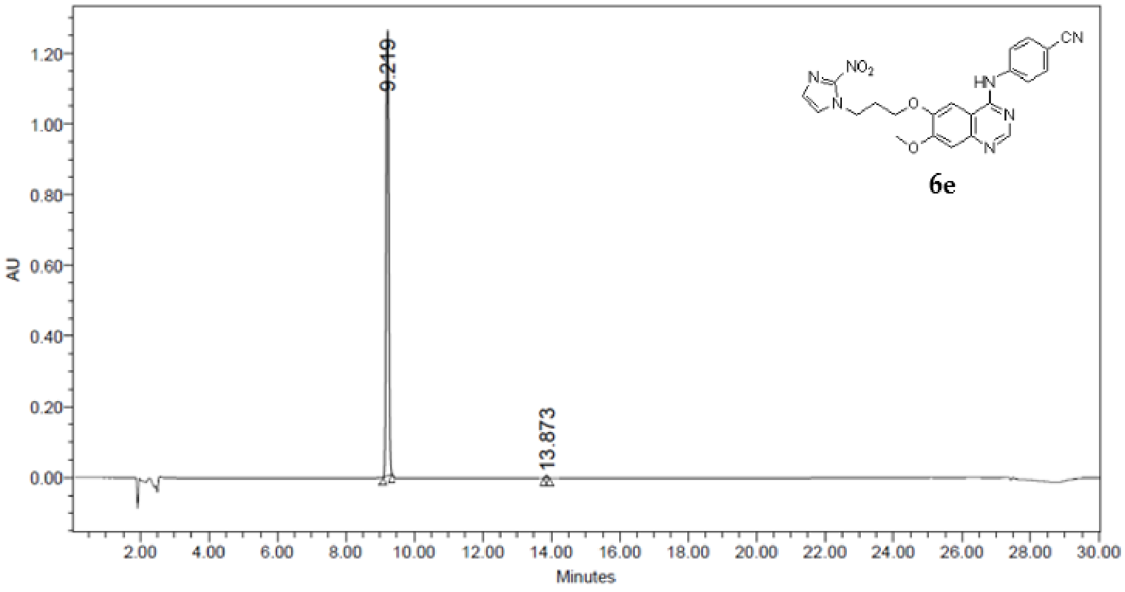

	RT	Area	% Area	Height
1	9.219	5899073	99.52	1248369
2	13.873	28452	0.48	100228

Figure S15. HPLC purity chart of compound **6e**

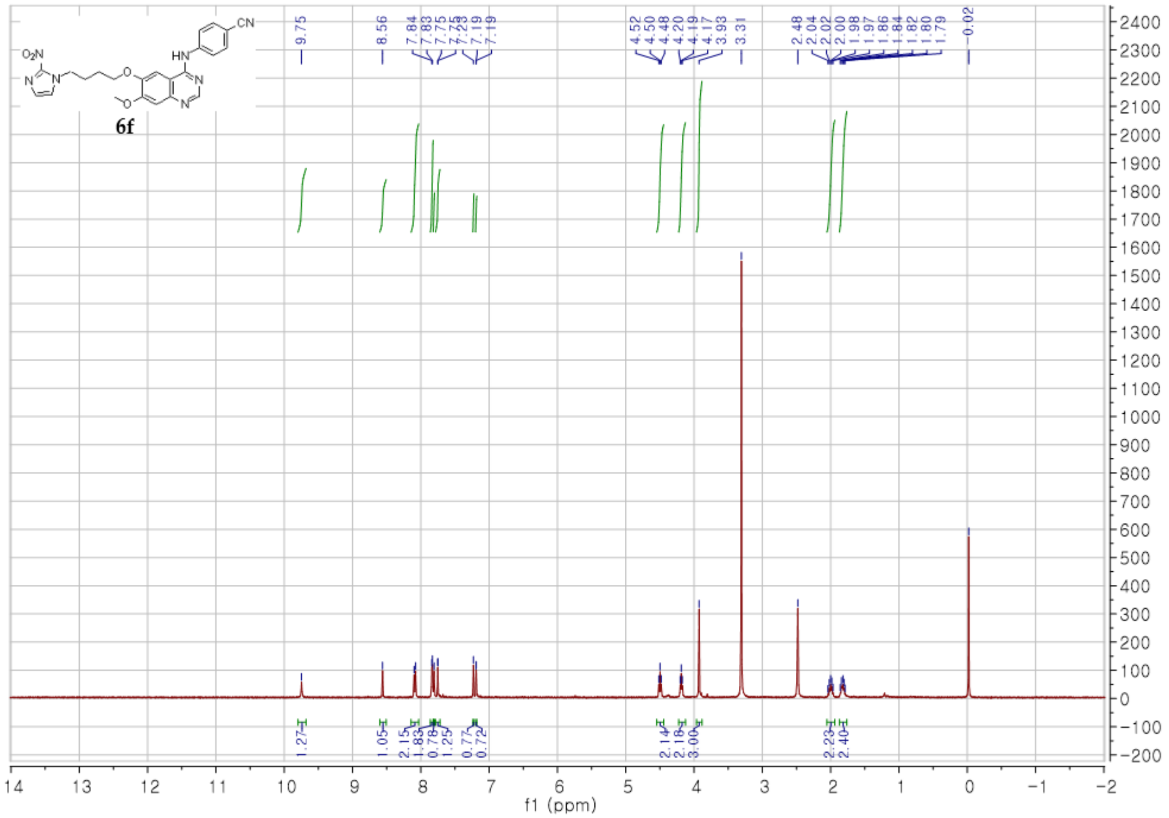

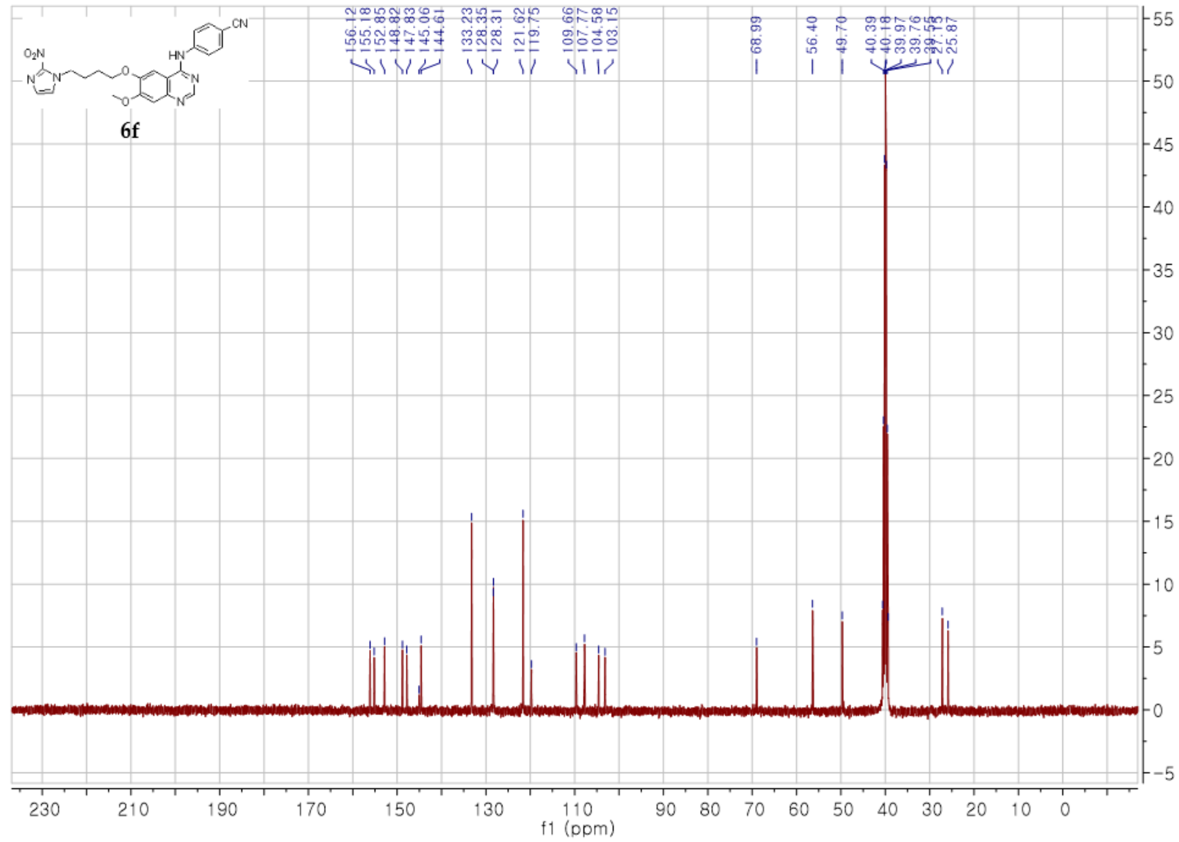

Figure S16. ^{1}H NMR and ^{13}C NMR spectrum of compound **6f**

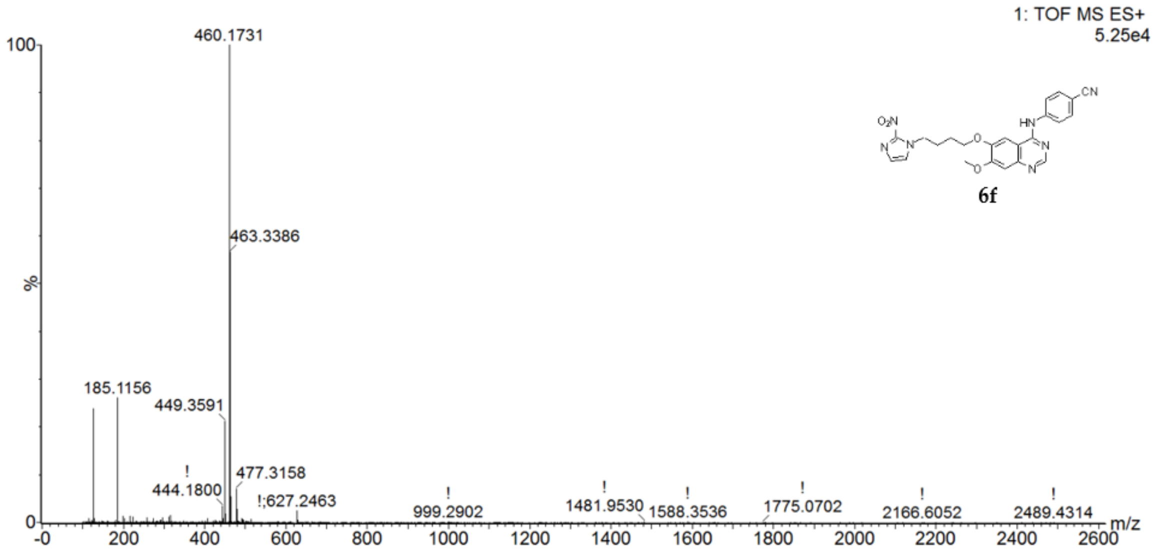

Figure S17. HRMS chart of compound **6f**

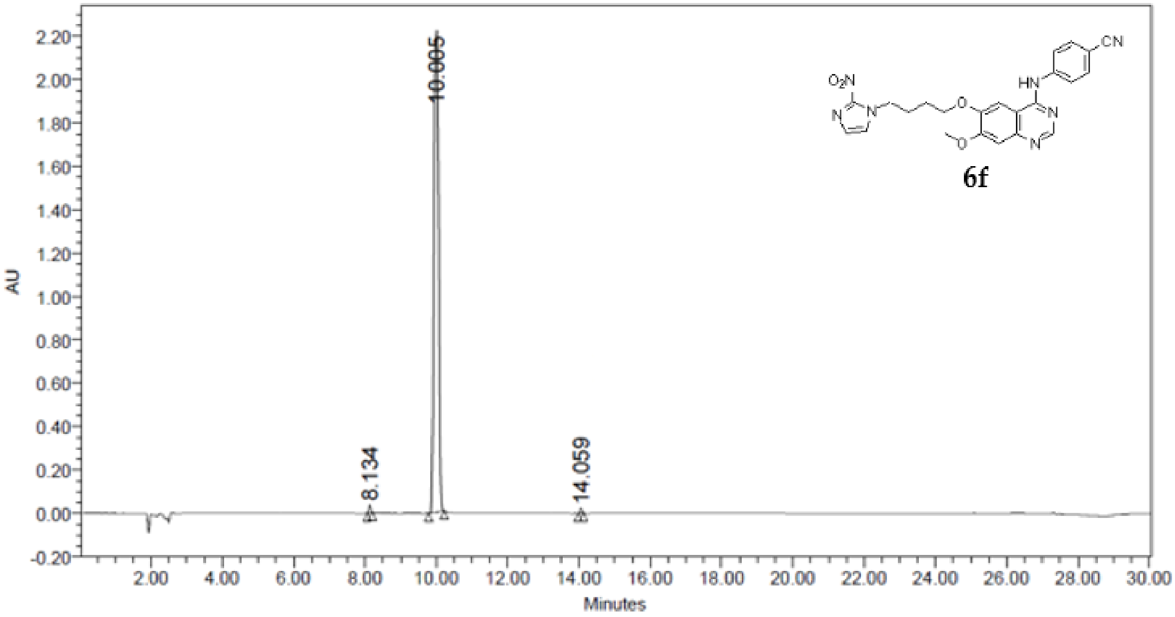

	RT	Area	% Area	Height
1	8.134	138984	0.57	35281
2	10.005	24276141	99.00	2251496
3	14.059	105874	0.43	22939

Figure S18. HPLC purity chart of compound **6f**

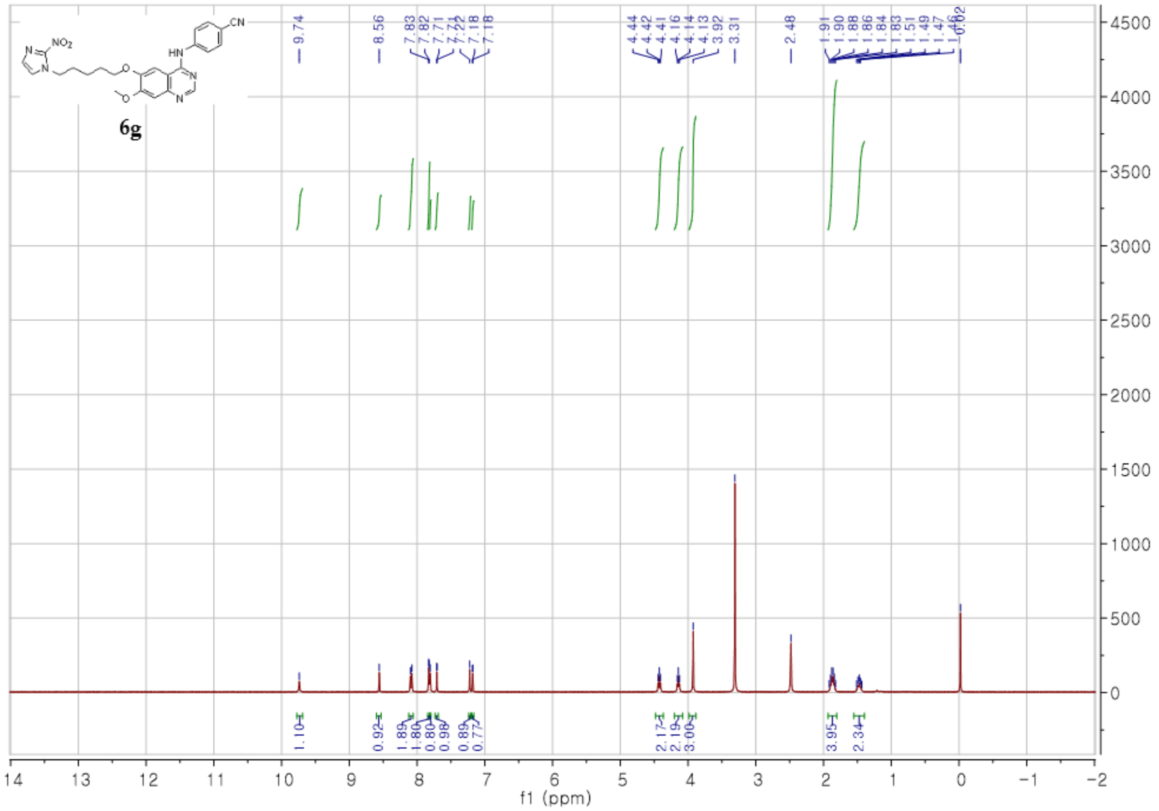

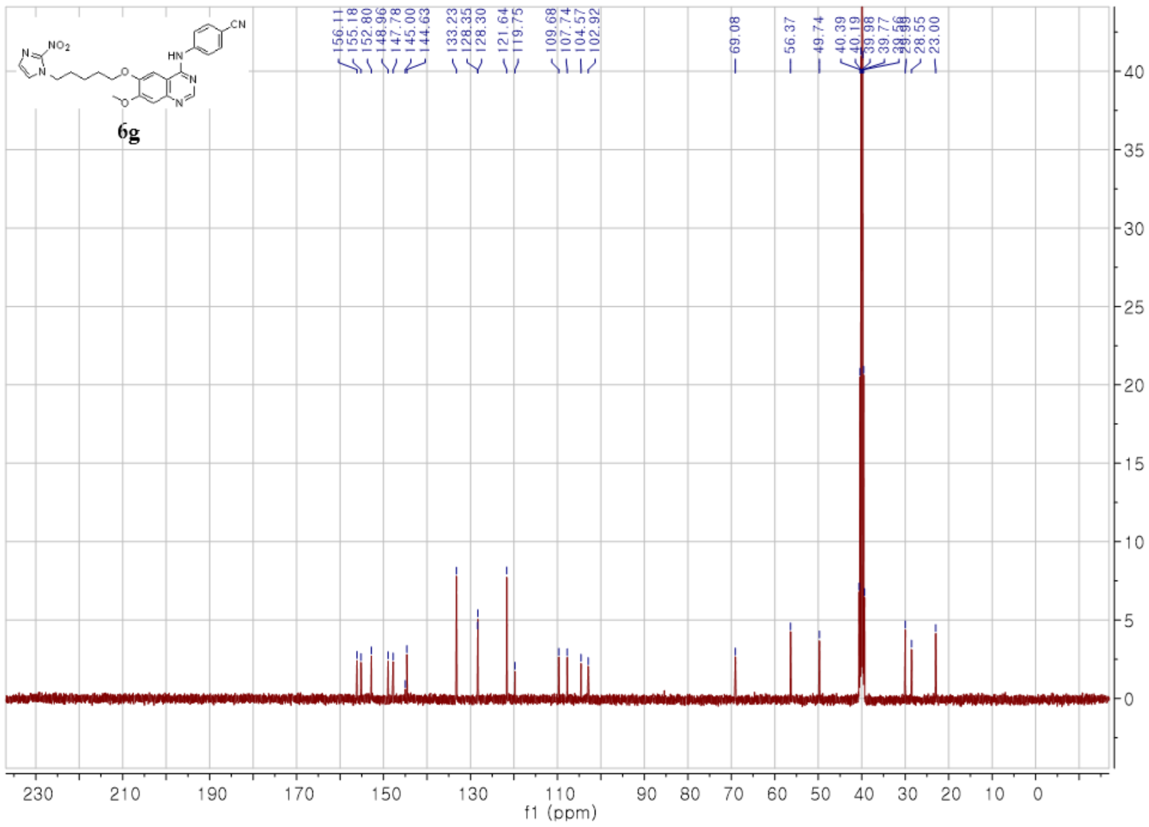

Figure S19. ^{1}H NMR and ^{13}C NMR spectrum of compound **6g**

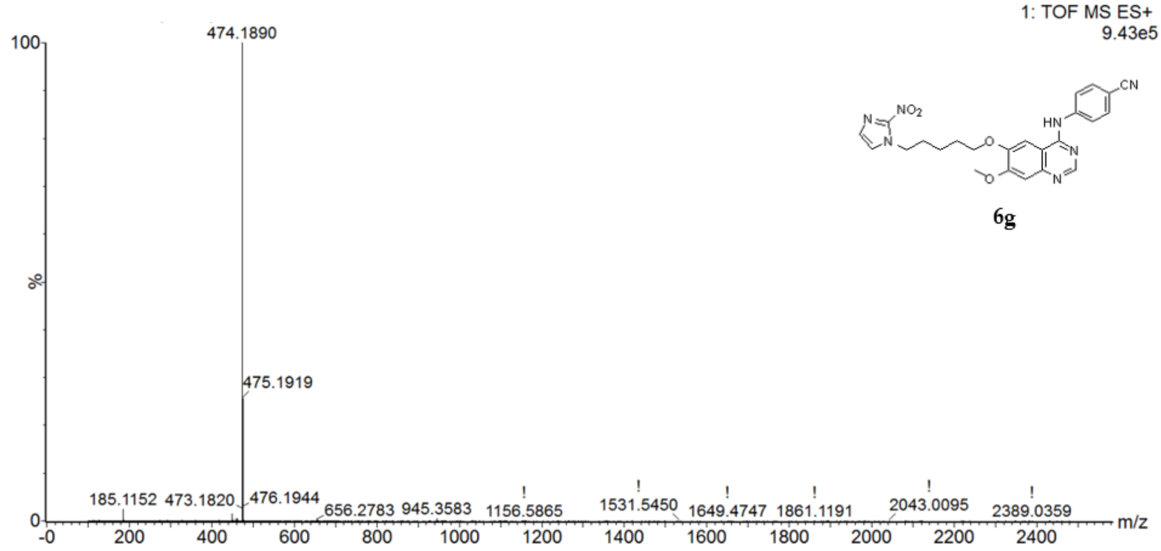

Figure S20. HRMS chart of compound **6g**

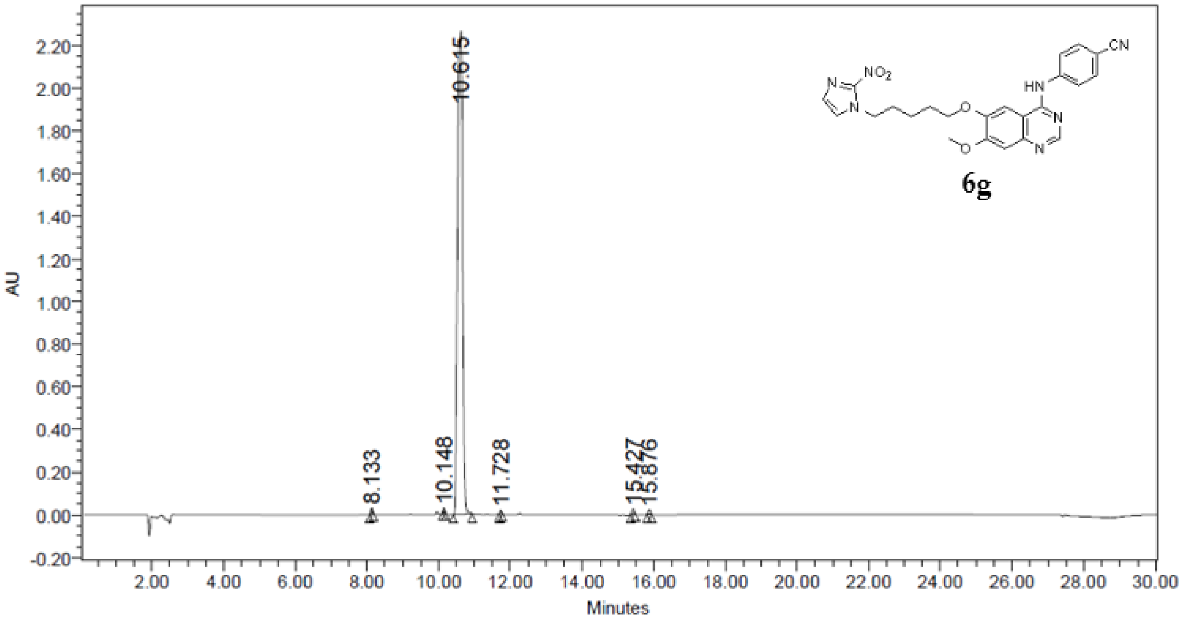

	RT	Area	% Area	Height
1	8.133	81329	0.36	23735
2	10.148	60083	0.27	19816
3	10.615	22019938	98.40	2303353
4	11.728	46571	0.21	13334
5	15.427	83948	0.38	21227
6	15.876	85521	0.38	19315

FigureS 21. HPLC purity chart of compound **6g**

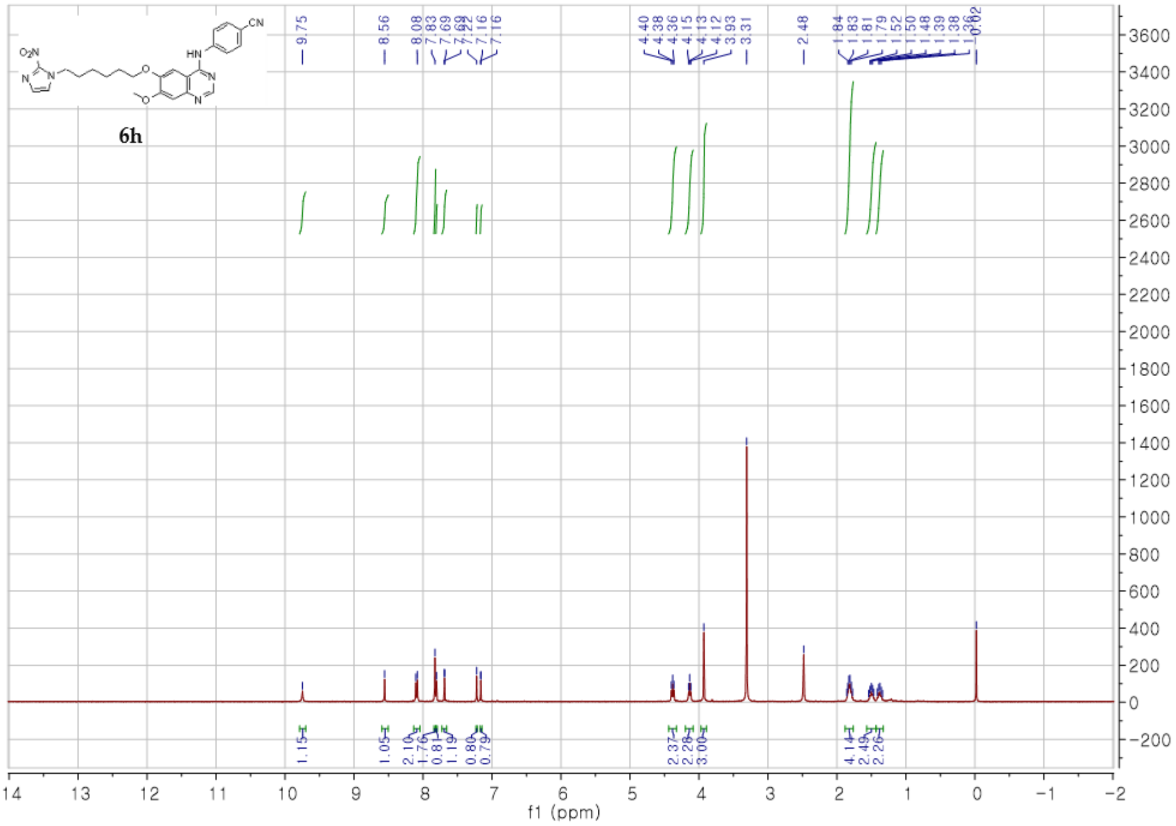

6h
f1 (ppm)

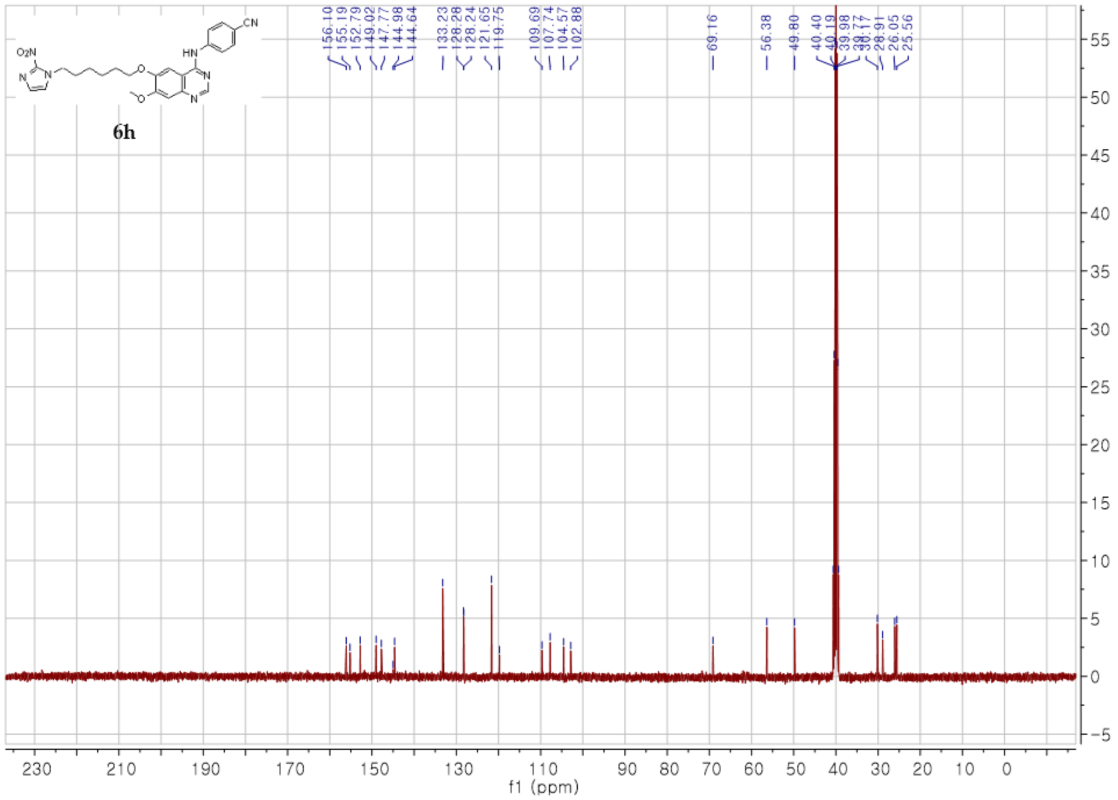

6h
f1 (ppm)

Figure S22. ^{1}H NMR and ^{13}C NMR spectrum of compound **6h**

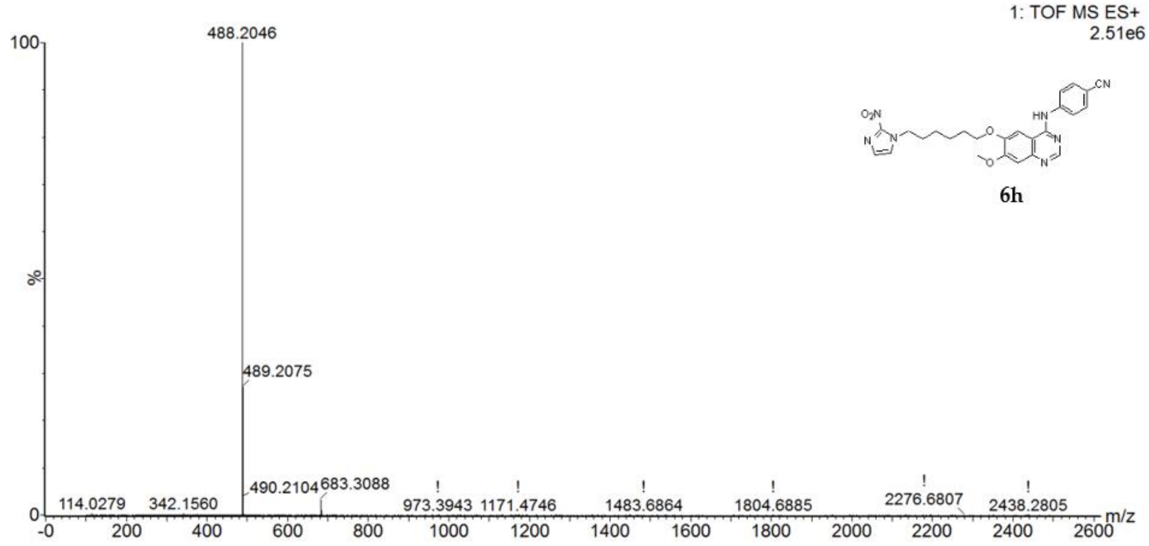

Figure S23. HRMS chart of compound **6h**

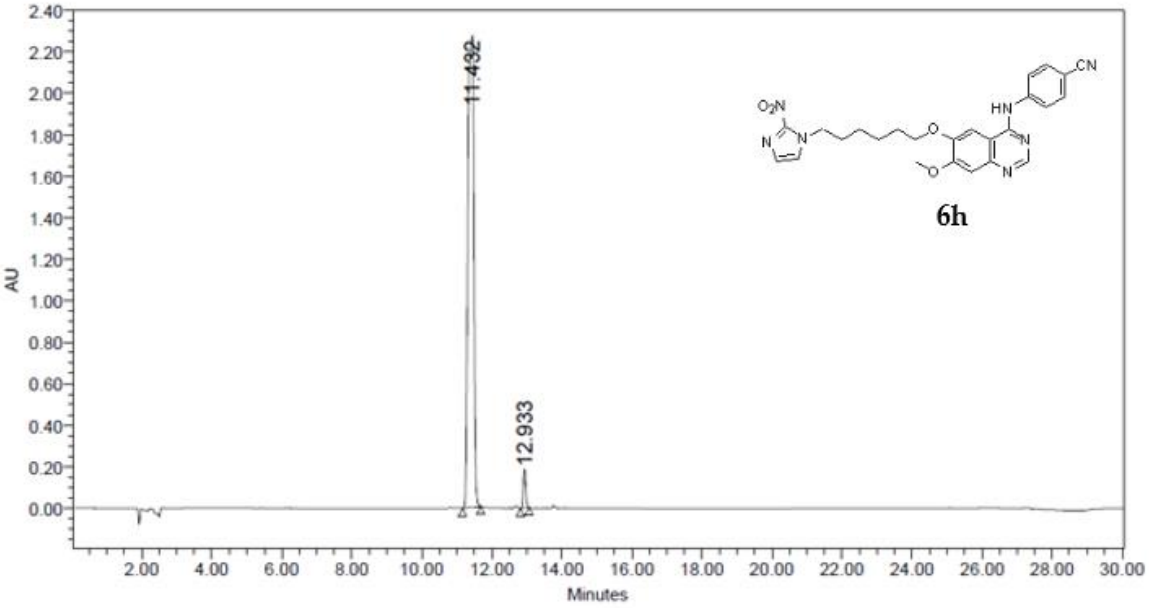

	RT	Area	% Area	Height
1	11.432	29133488	97.08	2318641
2	12.933	876167	2.92	185494

Figure S24. HPLC purity chart of compound **6h**

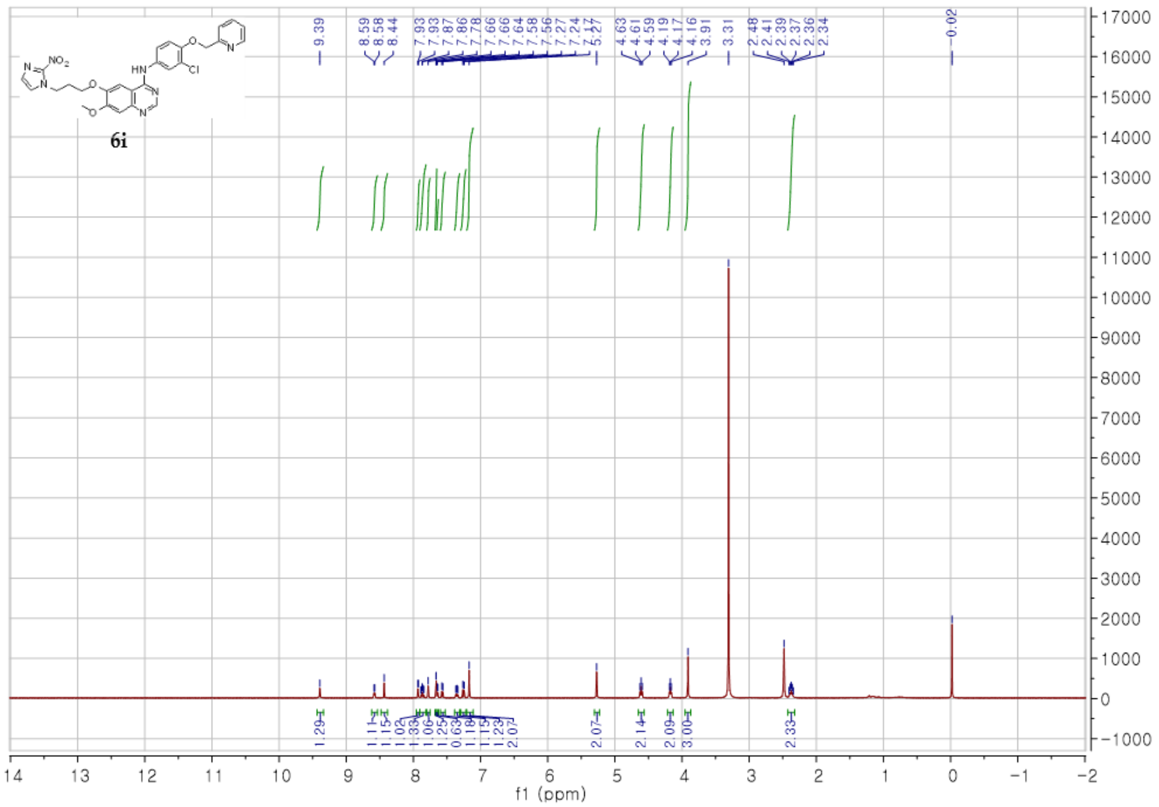

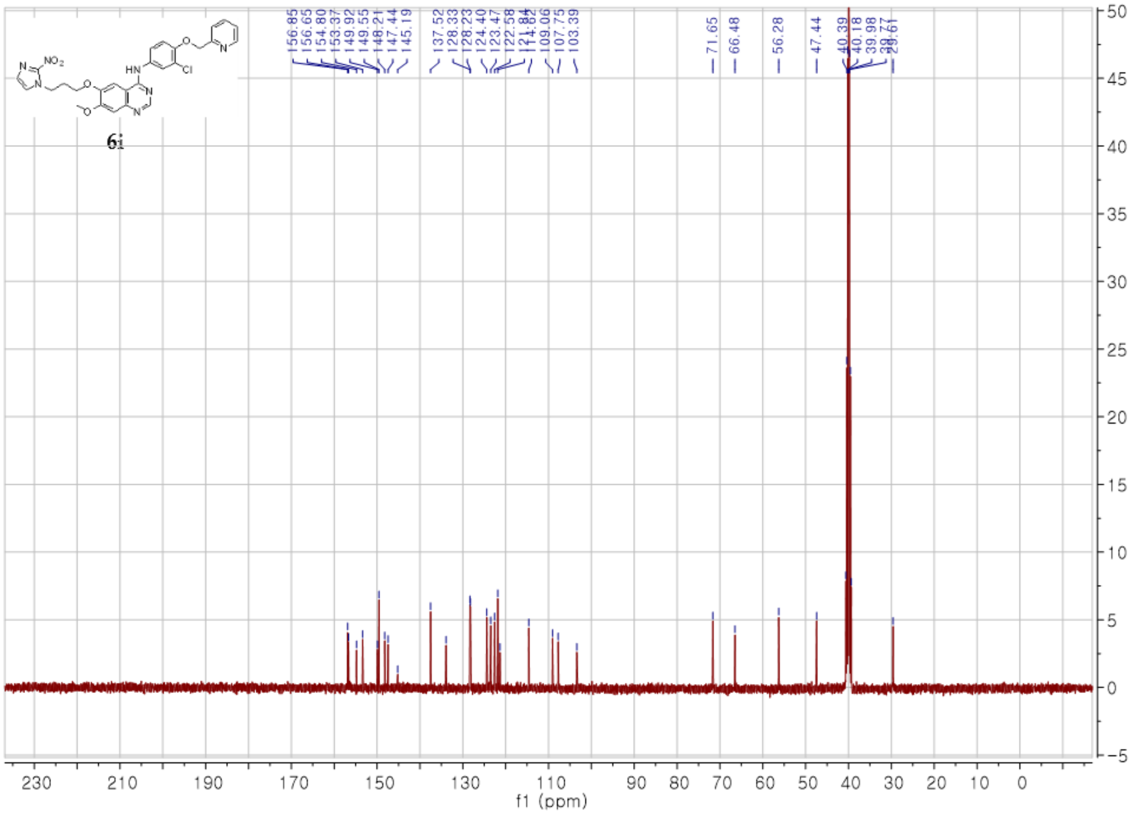

Figure S25. ^{1}H NMR and ^{13}C NMR spectrum of compound **6i**

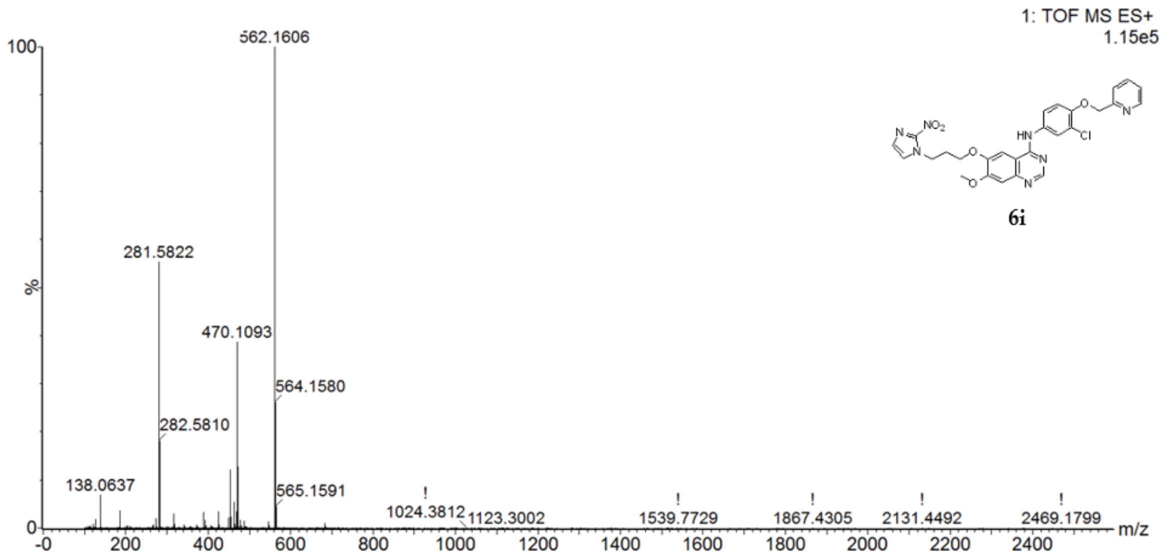

Figure S26. HRMS chart of compound **6i**

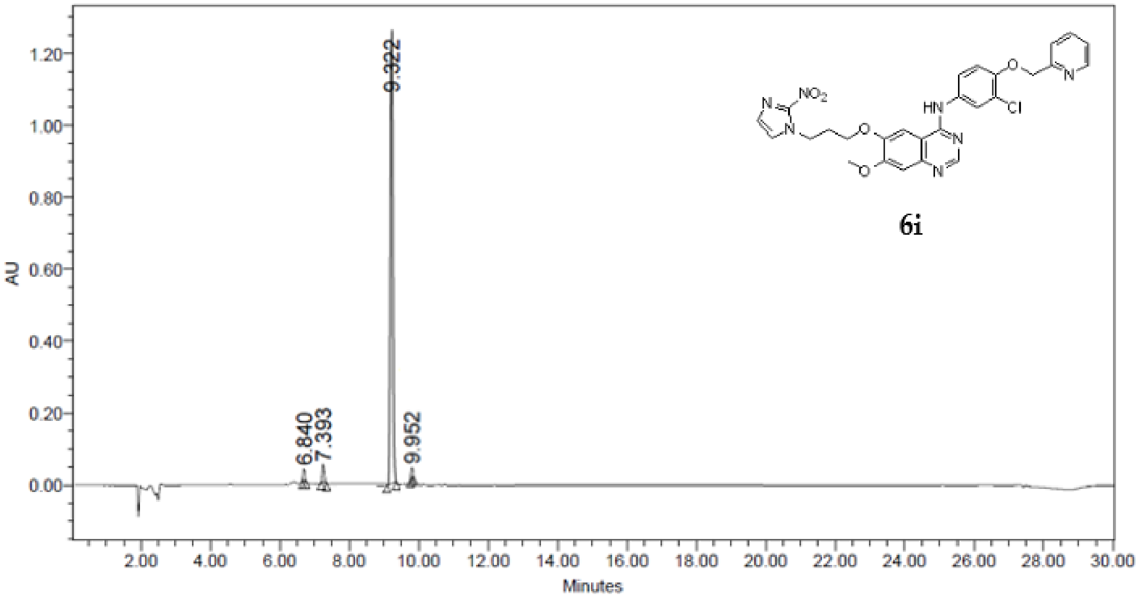

	RT	Area	% Area	Height
1	6.840	75915	1.22	49572
2	7.393	129429	2.08	51983
3	9.322	5938203	95.43	1262012
4	9.952	79026	1.27	50241

Figure S27. HPLC purity chart of compound **6i**

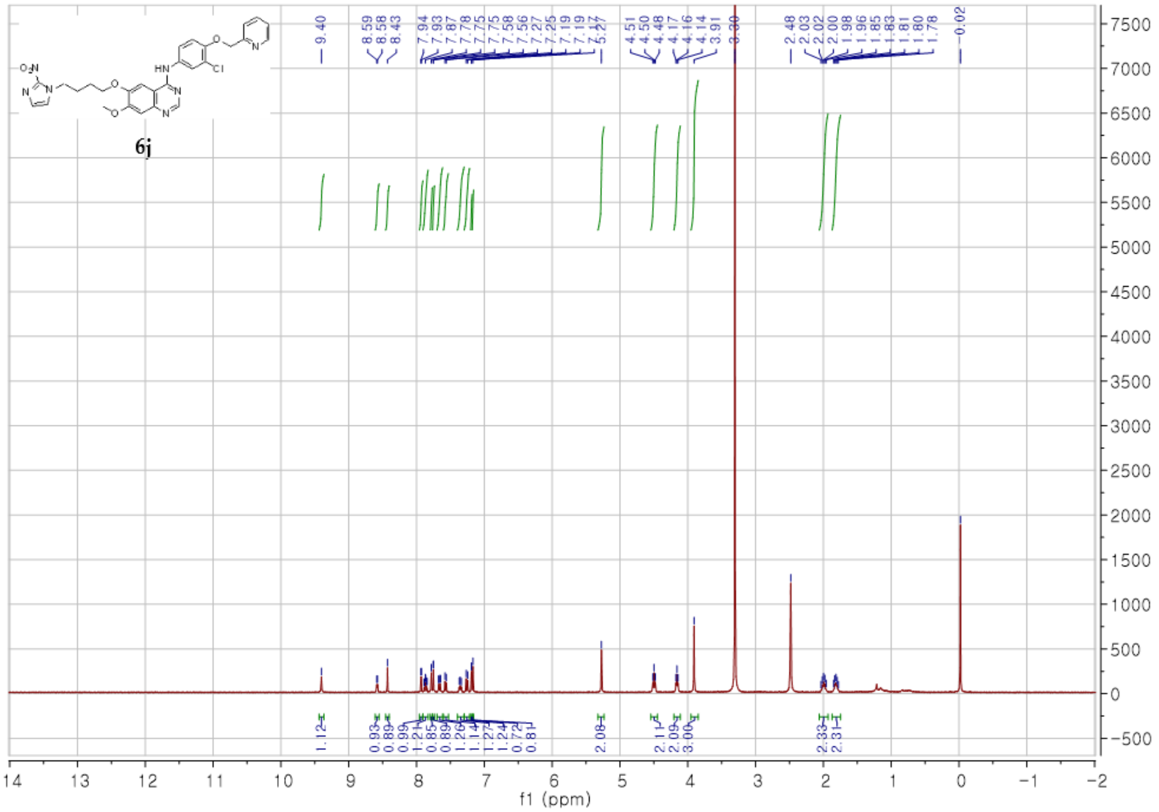

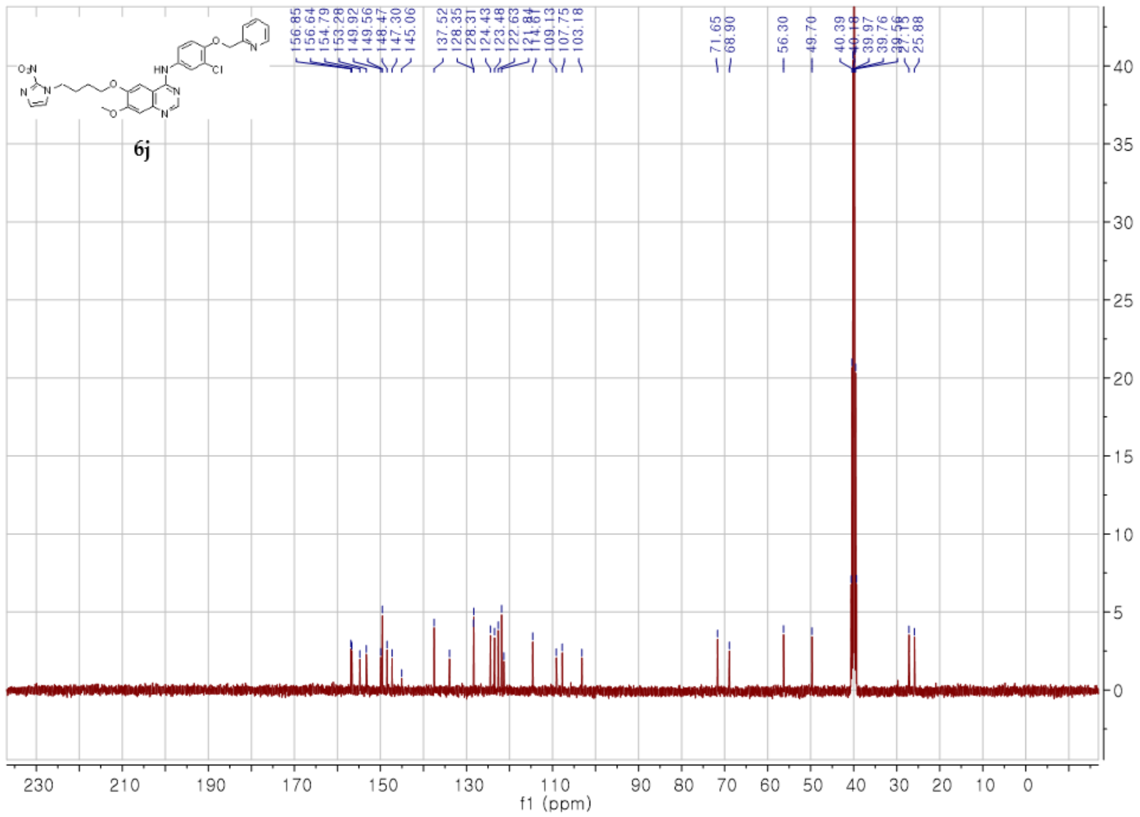

Figure S28. ^{1}H NMR and ^{13}C NMR spectrum of compound **6j**

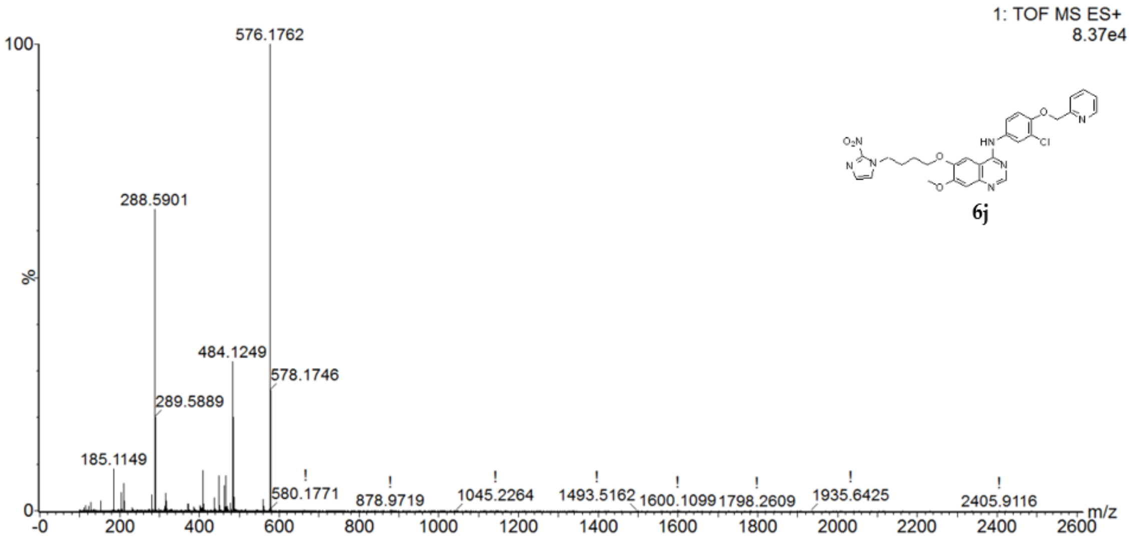

Figure S29. HRMS chart of compound **6j**

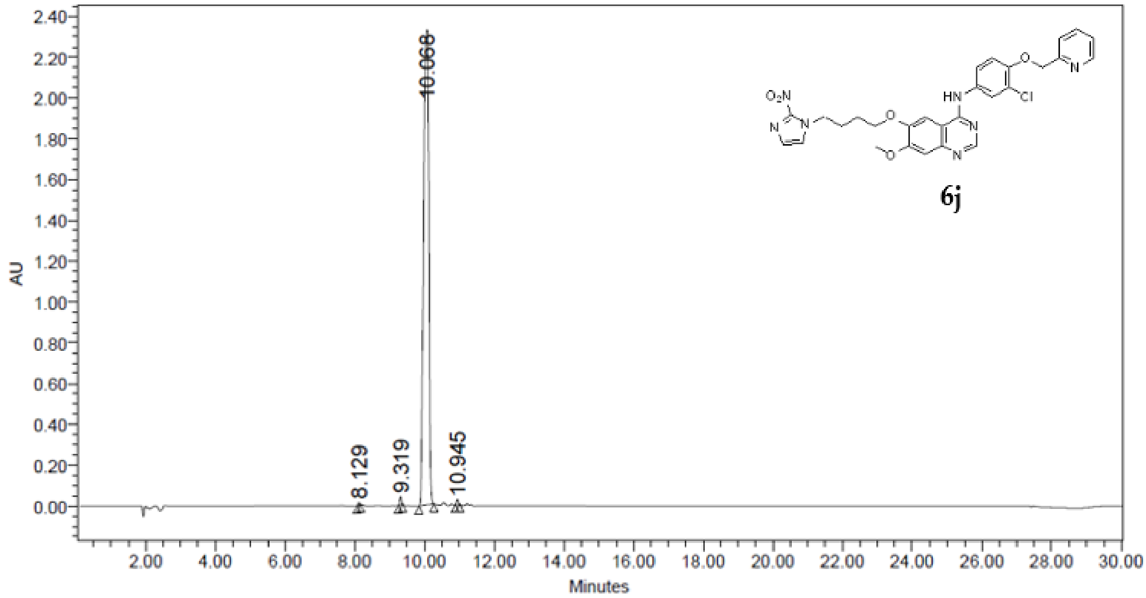

	RT	Area	% Area	Height
1	8.129	42877	0.17	12549
2	9.319	150730	0.61	39397
3	10.068	24464479	98.85	2345532
4	10.945	91981	0.37	24191

Figure S30. HPLC purity chart of compound **6j**

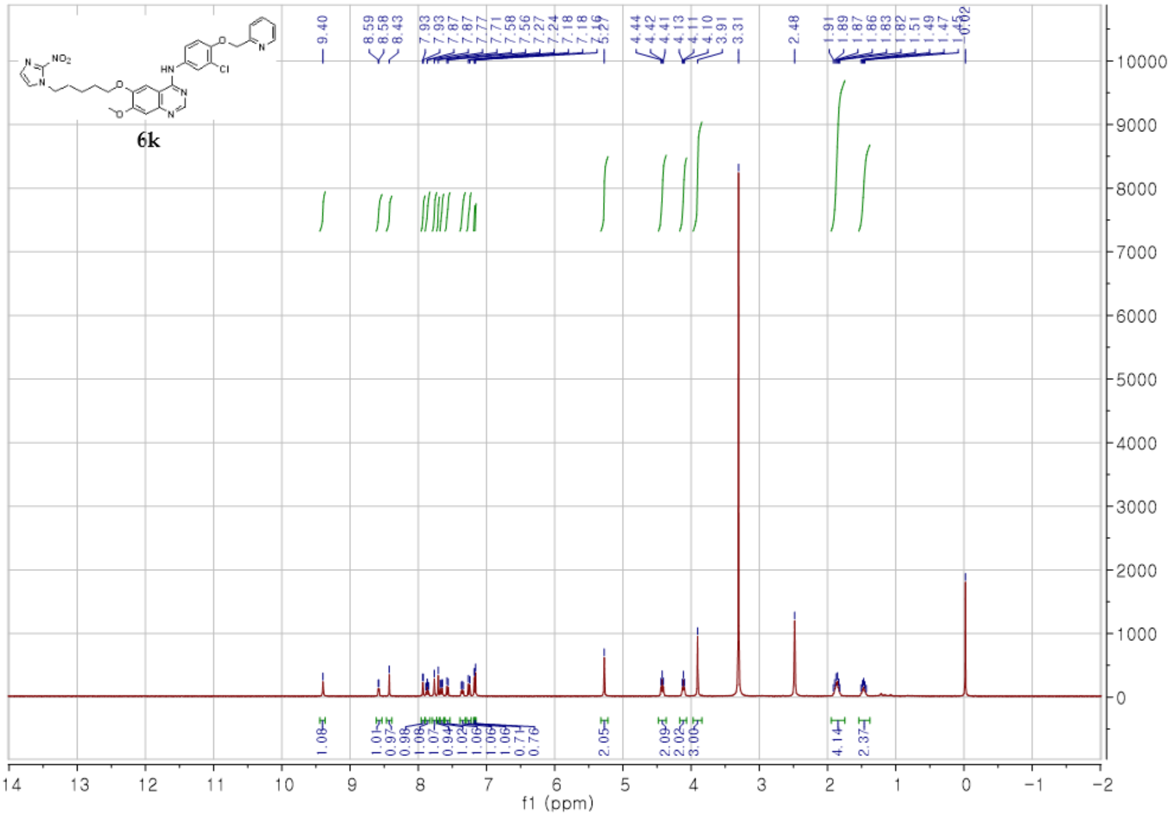

6k

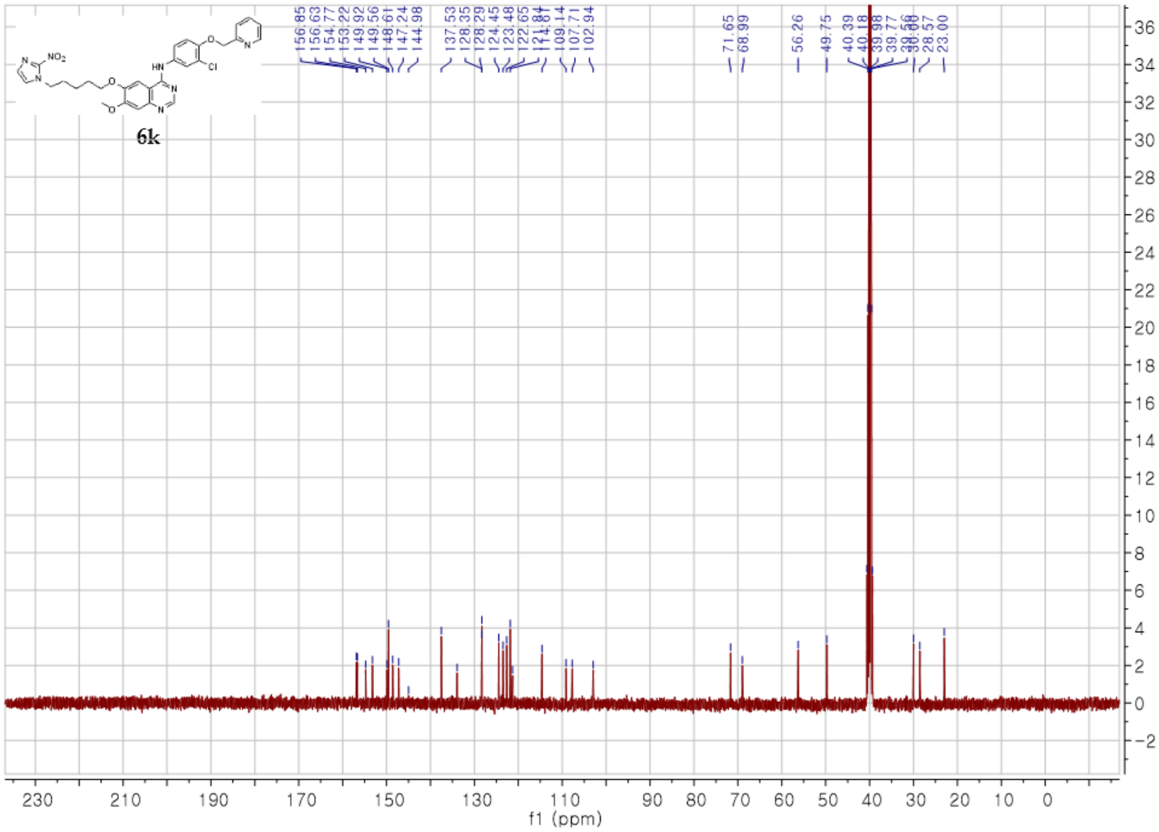

6k

Figure S31. ^{1}H NMR and ^{13}C NMR spectrum of compound **6k**

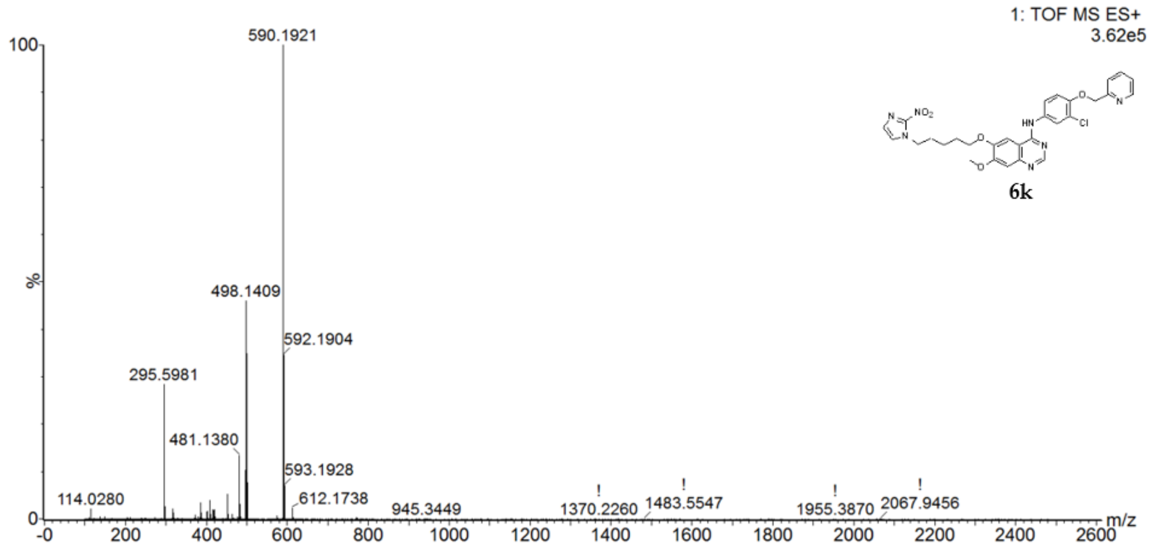

Figure S32. HRMS chart of compound **6k**

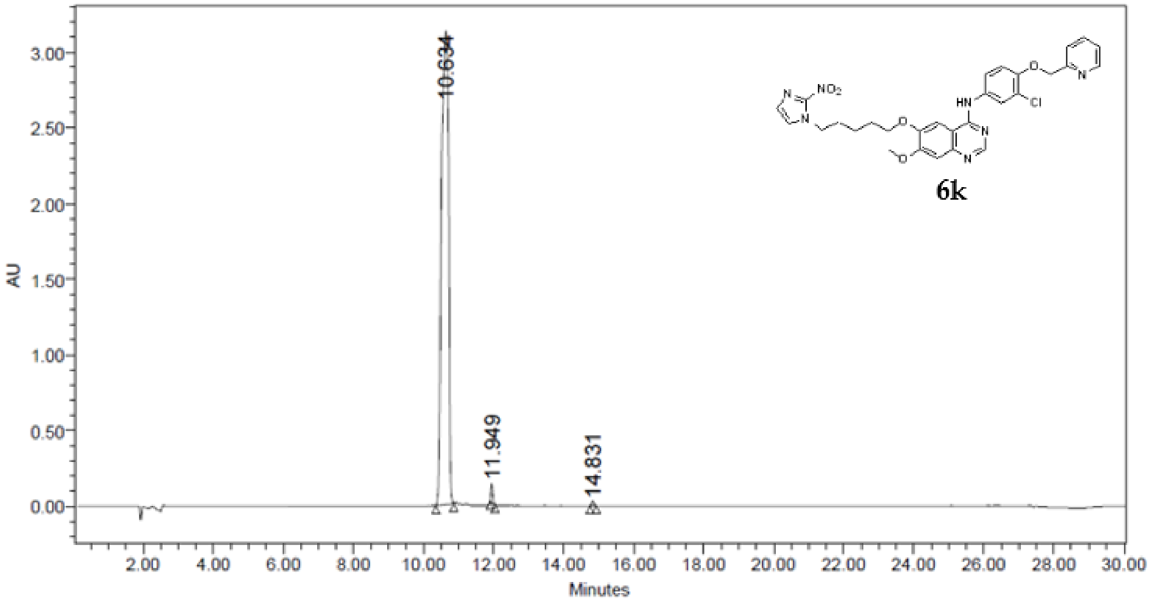

	RT	Area	% Area	Height
1	10.634	43565936	98.51	3120473
2	11.949	502402	1.14	124776
3	14.831	155269	0.35	29530

Figure S33. HPLC purity chart of compound **6k**

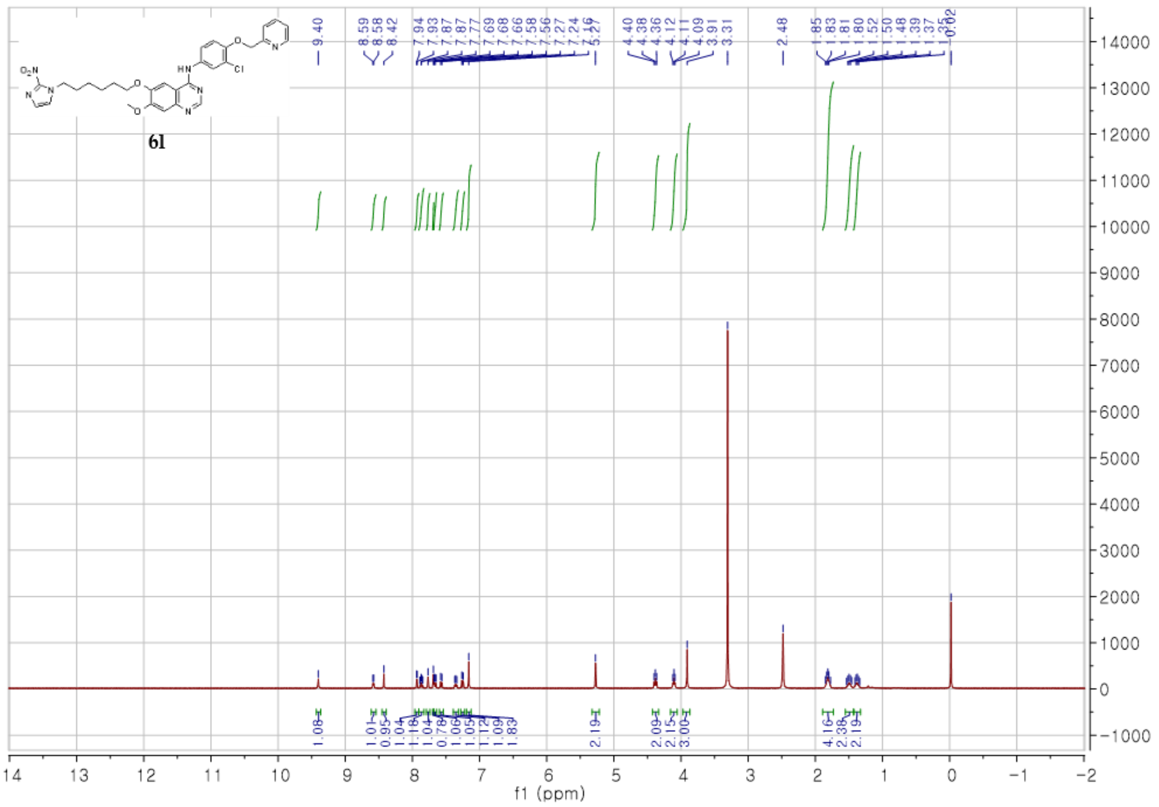

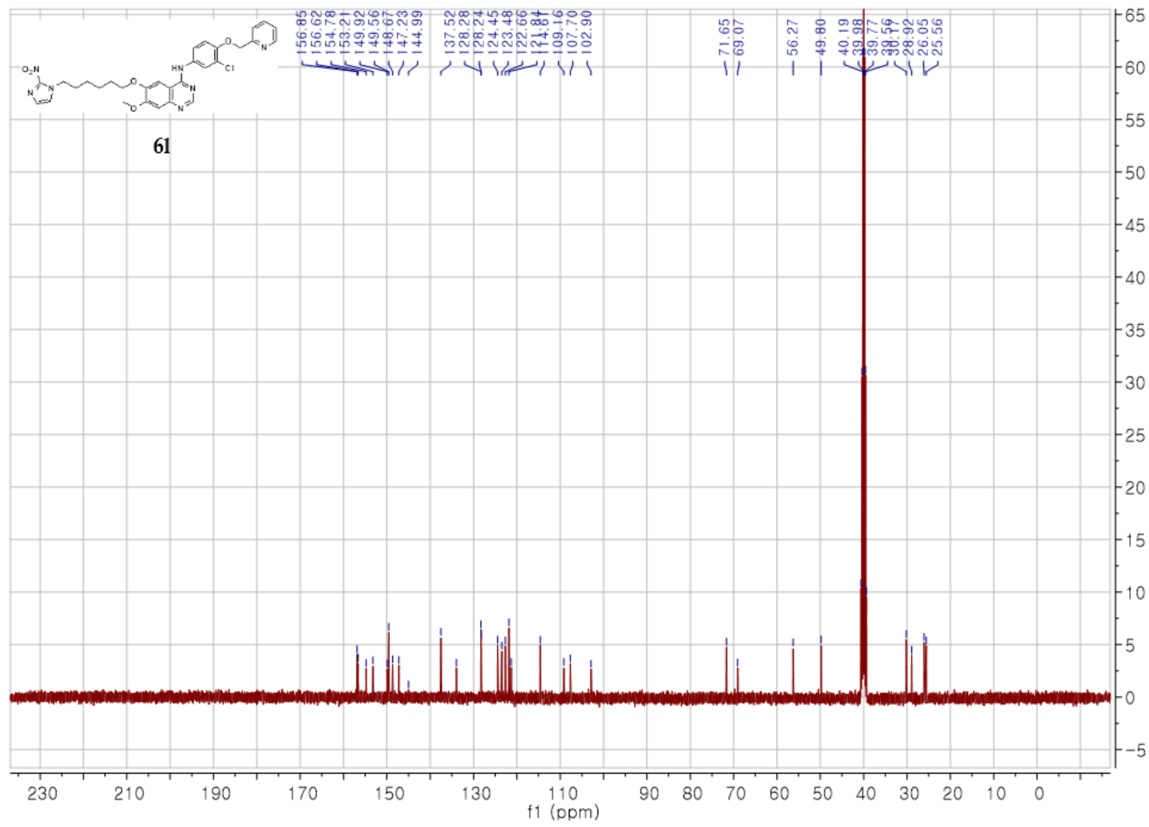

Figure S34. ^{1}H NMR and ^{13}C NMR spectrum of compound **6l**

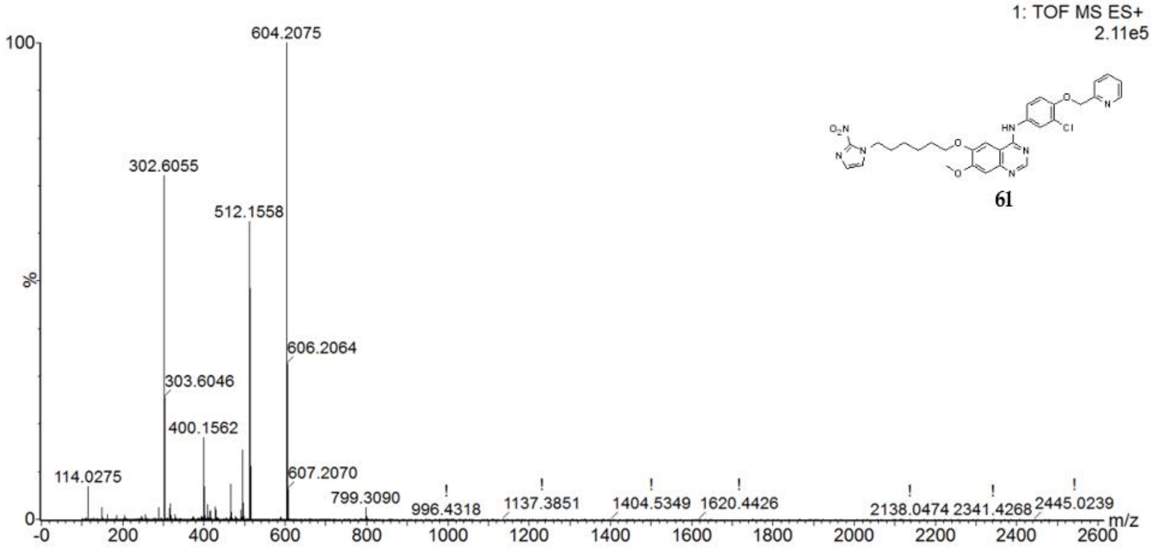

Figure S35. HRMS chart of compound **6l**

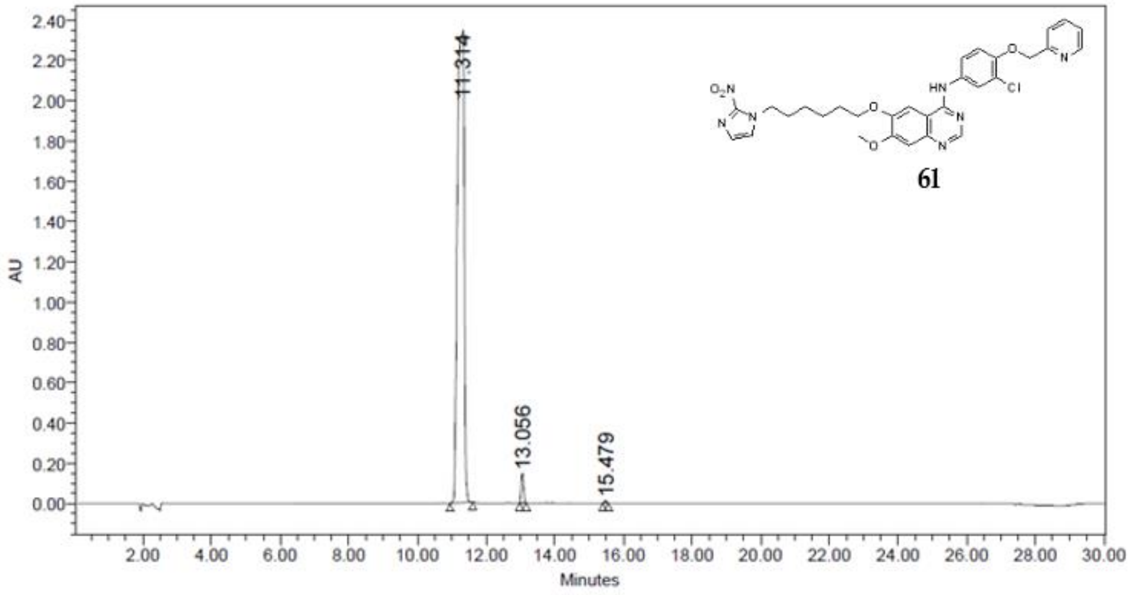

	RT	Area	% Area	Height
1	11.314	32519891	97.79	2402393
2	13.056	666792	2.01	142434
3	15.479	68408	0.21	13392

Figure S36. HPLC purity chart of compound **6l**

References

1. Release, S., *3: Desmond molecular dynamics system, DE Shaw research, New York, NY, 2017.* Maestro-Desmond Interoperability Tools, Schrödinger, New York, NY, 2017.
2. Harder, E., et al., *OPLS3: a force field providing broad coverage of drug-like small molecules and proteins.* Journal of chemical theory and computation, 2016. **12**(1): p. 281-296.
3. Jorgensen, W.L., et al., *Comparison of simple potential functions for simulating liquid water.* The Journal of chemical physics, 1983. **79**(2): p. 926-935.
4. Neria, E., S. Fischer, and M. Karplus, *Simulation of activation free energies in molecular systems.* The Journal of chemical physics, 1996. **105**(5): p. 1902-1921.
5. Manual, D.U., *Desmond2. 2.* 2009.
6. Martyna, G.J., M.L. Klein, and M. Tuckerman, *Nosé–Hoover chains: The canonical ensemble via continuous dynamics.* The Journal of chemical physics, 1992. **97**(4): p. 2635-2643.
7. Martyna, G.J., D.J. Tobias, and M.L. Klein, *Constant pressure molecular dynamics algorithms.* The Journal of chemical physics, 1994. **101**(5): p. 4177-4189.
